# Emergence of transferable daptomycin resistance in Gram-positive bacteria

**DOI:** 10.1038/s44259-025-00109-z

**Published:** 2025-04-26

**Authors:** Tessa Marciniak, Lukas Kirchner, Silver A. Wolf, Birgit Walther, Thorsten Bischler, Justin Nyasinga, Revathi Gunturu, Torsten Semmler, Tom Gräfenhan, Andrew Whitelaw, Oliver Scherf-Clavel, Ulrike Holzgrabe, Wilma Ziebuhr

**Affiliations:** 1 Institute of Molecular Infection Biology, University of Wurzburg, Wurzburg, Germany; 2 Institute for Pharmacy and Food Chemistry, University of Wurzburg, Wurzburg, Germany; 3 Genome Competence Centre (MF1), Robert Koch Institute, Berlin, Germany; 4 Microbiological Risks (II 1.4), German Environment Agency, Berlin, Germany; 5 Core Unit Systems Medicine, University of Wurzburg, Wurzburg, Germany; 6 The Aga Khan University Hospital, Nairobi, Kenya; 7 Division of Medical Microbiology, Stellenbosch University, Cape Town and National Health Laboratory Service, Tygerberg Hospital, Cape Town, South Africa; 8 Department of Pharmacy, Clinical Pharmacy and Pharmacotherapy, Ludwig-Maximilians-University, Munich, Germany

**Keywords:** Microbiology, Antimicrobials, Antimicrobial resistance

## Abstract

Daptomycin (DAP) is a last-resort antibiotic to treat infections by multiresistant Gram-positive pathogens, including methicillin-resistant *Staphylococcus aureus* (MRSA) and vancomycin-resistant enterococci. DAP resistance and clinical treatment failure has been associated with adaptive chromosomal mutations, but so far not with transmissible resistance traits. Here we report for the first time an acquired DAP-R determinant (named *drc*) that we detected in a livestock-associated *Mammaliicoccus sciuri* isolate*. drc* consists of a two-gene operon (*drcAB)* that is controlled by an adjacent two-component system (*drcRS*). The DrcAB proteins, which mediate DAP inactivation, are similar to BceAB-like antimicrobial peptide transporters of Gram-positives, but are distinct from currently known systems. The mobile *drc* locus is functional in various bacterial backgrounds, including MRSA. It circulates primarily among Gram-positives in the environment, but also in commensal staphylococci and enterococci, suggesting a risk of transmission into pathogens and emphasizing the importance of low and apathogenic microorganisms as resistance gene reservoirs.

## Introduction

Daptomycin (DAP) is a cyclic lipopeptide antibiotic with activity against Gram-positive bacteria, including high priority pathogens such as methicillin-resistant *Staphylococcus aureus* (MRSA) and vancomycin resistant enterococci (VRE)^1^. According to the World Health Organization’s (WHO) Access, Watch, Reserve (AWaRe) classification for antibiotics, DAP is currently listed as a reserve compound and approved for the treatment of complicated skin and soft tissue infections as well as for treating *S. aureus* bacteraemia^2^. DAP represents a natural compound that was originally isolated from the soil bacterium *Streptomyces roseosporus*. It consists of a polypeptide ring of ten amino acids (DAP core) and a short stretch of another three amino acids to which a lipophilic fatty acid tail is attached (DAP chain)^1^. The precise mode of action of DAP is not fully understood, but the compound acts at the bacterial cytoplasmic membrane and requires complex formation with Ca^2+^ ions to exert its bactericidal effect^1^. Recent work suggests that DAP-Ca^2+^ binds both membrane phospholipids – with phosphatidylglycerol representing an important DAP target – and lipid-coupled cell wall synthesis precursors during cell division, resulting in the interruption of peptidoglycan synthesis, followed by membrane disintegration and cell death^3^.

The overall daptomycin resistance rates (DAP-R) are still low and are usually less than 1% for *S. aureus* and 0.5 to 5% for enterococci^4,5^. Nevertheless, clinical treatment failure has been widely reported in *S. aureus* and enterococcal infections, with reduced susceptibility of isolates often developing gradually under DAP therapy^6,7^. The underlying mechanism of DAP-R is not uniform and is instead attributed to the accumulation of point mutations within a number of genes involved in membrane phospholipid metabolism and cell wall homeostasis, resulting in aberrations of key cell membrane functions and cell wall composition^8^. Specifically, mutations in *mprF* and *dltABCD* are proposed to confer DAP-R in staphylococci through modulation of surface charge and impaired DAP binding^9–11^. Other mutations typically affect two-component regulatory systems (TCS) and ABC transporters involved in antimicrobial peptide (AMP)-sensing and cell wall stress response control^12–14^ as well as aberrations in membrane lipid metabolism^15,16^. None of these reported DAP-R mechanisms are transmissible, suggesting that DAP-R is developing within individual strains upon DAP exposure, but not by acquisition of specific resistance genes through horizontal gene transfer (HGT).

As part of studies on antimicrobial resistance (AMR) in livestock, we previously isolated a number of multiresistant *Staphylococcus sciuri* strains from pig farms^17^. *S. sciuri* is a versatile coagulase-negative staphylococcal species that mainly occurs as an animal commensal but is also regularly recovered from soil environments^18,19^. *S. sciuri* was recently assigned to a new genus (*Mammaliicoccus*) within the Staphylococcaceae family, with *Mammaliicoccus sciuri* being the type species^20^. *M. sciuri* is known to readily acquire AMR genes and is considered as a reservoir for the evolution and spread of emerging resistance genes in staphylococci^19^. Among the *M. sciuri* isolates from conventional pig farms, strain *M. sciuri* TS92 attracted our attention by its DAP minimum inhibitory concentration (MIC) of 64 µg/mL. With a DAP breakpoint for staphylococci of 1 µg/mL (according to EUCAST guidelines), we classified *M. sciuri* TS92 as high-level DAP resistant. In two parallel studies, we recently demonstrated that the DAP-R phenotype of TS92 is associated with chemical modification of the antibiotic^21,22^. In the report presented here, we describe the identification and genetic organization of the underlying resistance genes, which we named *drc* (for daptomycin resistance cluster). The *drc* locus, which encodes a BceAB-like antimicrobial peptide transporter system, was found to be fully functional in various Gram-positive bacterial backgrounds, including *S. aureus* and clinically relevant MRSA. We further show that the *drc* locus is mobile and detectable in commensal staphylococci and enterococci from animal origin as well as in a wide range of Gram-positive bacteria from the environment. In the light of these findings, we discuss the importance of low-pathogenic and environmental microorganisms as potent sources of resistance determinants for clinically important pathogens.

## Results

### DAP induces transcription of a *bce*-like gene cluster in *M. sciuri* TS92

**Fig. 1:**
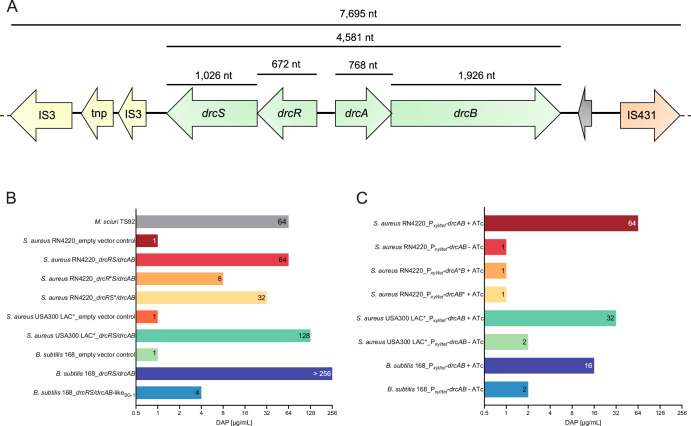
Detection of a novel gene cluster (*drc)* mediating DAP-R. **A** Organisation of the daptomycin resistance cluster (*drc*) detected within the *M. sciuri* TS92 genome. *drcAB* genes form an operon and encode a Bce-like ABC membrane transporter homologue. Upstream of *drcAB*, a two-component regulatory system (*drcRS*) is located on the opposite strand. Grey arrow depict putative ORF of unknown function. The locus is flanked by insertion sequence elements (IS) and transposase (*tnp*) genes. The drawing is not to scale. Respective gene sizes are indicated on top. **B** DAP MICs of bacteria carrying different *drc* variants under control of the native two component system *drcRS* on a plasmid vector. **C** DAP MICs of bacteria carrying different *drc* variants under control of an ATc-inducible promoter P_*xyl/tet*_ on a plasmid vector. *M*.–*Mammaliicoccus*, *S*.–*Staphylococcus*, *B*. –*Bacillus*, ATc – anhydrotetracycline, SG-1 – *Novibacillus thermophilus* strain SG-1. Asterisks indicate inactivated genes carrying an early stop codon. Results are from three biological replicates.

The *M. sciuri* TS92 isolate was originally recovered from a dust sample collected at a pig farm. TS92 repeatedly displayed high daptomycin MIC values of 64 µg/mL (Fig. 1B). Whole genome sequencing (WGS) of the isolate and subsequent single nucleotide polymorphism (SNP) analysis of *mprF* and *dltABCD* did not reveal mutations previously associated with DAP-R. For identifying the underlying DAP-R mechanism, TS92 was briefly exposed to high DAP concentrations, and the transcription profiles of DAP-treated and -untreated TS92 bacteria were determined by RNA-Seq. Two genes (TS92_00079, TS92_00080) were found to be significantly up-regulated upon DAP exposure in comparison to the untreated control (*i.e.*, 128-fold change, Table S3). We termed these genes *drcA* and *drcB* (for daptomycin resistance cluster). The encoded proteins show homologies to Bce-type ABC export and permease proteins, which in turn belong to a large class of AMP membrane-associated ABC transporters^23^. Such systems are widespread across Gram-positive bacteria, in which they are controlled via neighbouring two-component regulatory systems (TCS) and are usually involved in AMP sensing and resistance to cell envelope-targeting antibiotics^23^. In TS92, the *drcA* and *drcB* genes are chromosomally encoded, with both open reading frames (ORFs) overlapping by 14 base pairs, suggesting organisation as an operon (Fig. 1A). Adjacent ORFs (TS92_0078, TS92_0077 on the opposite strand) harbour genes encoding a putative response regulator (named *drcR*) and a putative sensor histidine kinase (named *drcS*) of a two-component system (TCS) (Fig. 1A).

### *drcAB* mediates high-level DAP resistance in various Gram-positive backgrounds

For further analysis, the entire *drc* locus (consisting of *drcRS* and *drcAB*) was cloned onto a plasmid vector and transformed into the DAP-sensitive *S. aureus* RN4220 strain. Upon *drc* acquisition, *S. aureus* RN4220 displayed the same DAP MIC of 64 µg/mL as *M. sciuri* TS92, while the DAP MICs of the *S. aureus* RN4220 empty vector control was 1 µg/mL (Fig. 1B). The data suggest that *drc* is indeed the cause of DAP-R in TS92. To investigate if all four *drc* genes are required to mediate the DAP-R phenotype, we cloned only the *drcAB* genes onto the plasmid and placed the operon under the control of an inducible promoter (*P*_*xyl/tet*_) that can be activated by adding anhydrotetracycline (ATc) to the growth medium. Upon induction of *drcAB* expression, *S. aureus* RN4220 displayed a DAP MIC of 64 µg/mL, demonstrating that the two genes are sufficient to mediate high-level DAP-R in the *S. aureus* RN4220 background (Fig. 1C). Further, we introduced early stop codons into the coding sequence of each of the *drc* genes on the vector and once again determined DAP MICs. Complete loss of the DAP-R phenotype was recorded when either *drcA* or *drcB* were inactivated, whereas manipulation of *drcR* or *drcS* reduced DAP MICs but did not reach the susceptibility levels of the control (Fig. 1B, C). This suggests that *drcAB* is mediating the DAP-R phenotype, while *drcRS* has rather a regulatory function.

### The *drc* locus is functional in MRSA

*S. aureus* RN4220 is an antibiotic susceptible, restriction modification-deficient reference strain which is widely used as cloning host in staphylococcal research. To test if the *drc* locus would also be expressed in a clinically relevant MRSA background, we transformed the MRSA strain *S. aureus* USA300 LAC* with the plasmid vector harbouring the entire wildtype *drc* locus from TS92. While the empty vector control had a DAP MIC of 1 µg/mL, acquisition of *drc* by *S. aureus* USA300 LAC* resulted in a DAP MIC of 128 µg/mL, which is higher than in the original *M. sciuri* TS92 strain and also in the *S. aureus* RN4220 background, both of which achieving DAP MICs of 64 µg/mL (Fig. 1B). ATc-mediated induction of *drcAB* expression on a plasmid led to a DAP MIC of 32 µg/mL in *S. aureus* USA300 LAC* (Fig. 1C). Also, when transforming the same plasmid into *B. subtilis* 168 (DAP MIC 2 µg/mL), induction of *drcAB* expression resulted in an increase of the DAP MIC (16 µg/mL) (Fig. 1C). However, when *B. subtilis* 168 was provided with the entire *drc* locus of TS92 on a plasmid, the DAP-MIC increased to a value of >256 µg/mL (Fig. 1B). We conclude from these experiments that the entire wildtype *drc* locus of TS92 is expressed upon transfer to other Gram-positive bacteria and mediates high DAP-R levels in clinically relevant MRSA as well as in *B. subtilis*.

### DrcAB inactivates DAP

**Fig. 2:**
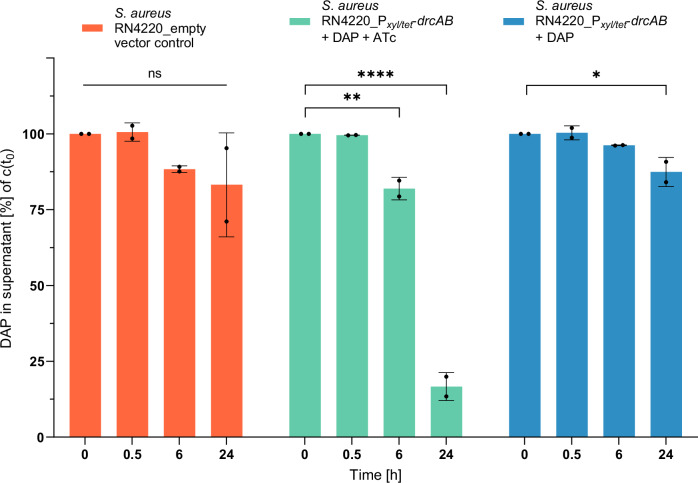
Fate of daptomycin upon *drcAB*-expression. Time course of DAP concentrations in supernatants of *drcAB*-expressing and non-expressing *S. aureus* RN4220 cultures as determined by LC-MS/MS. *drcAB* (under control of the ATc-inducible P_xyl/tet_ promoter) was provided on a plasmid vector to *S. aureus* RN4220. Orange bars: empty vector control; Green bars: ATc-induced *drcAB* expression; Blue bars: non-induced (basal) *drcAB* expression without ATc. Mean with SD from biological replicates (*n* = 2 with technical replicates (*n* = 5) each) are shown. One-Way ANOVA with Dunnett multiple comparisons test was performed for statistical analysis. ns not significant; *P < 0.05; **P < 0.01; ****P < 0.001.

Next, we were interested in understanding how *drcAB* causes DAP-R. The general principles that bacteria commonly employ to protect themselves against antibiotics comprise (i) modification of the antibiotic target in the bacterial cell, (ii) interference with antibiotic transport, and finally, (iii) inactivation of the hazardous compound by degradation or chemical modification. To test for putative DAP inactivation, we employed LC-MS/MS to monitor the fate of the antibiotic in supernatants of *drcAB-*expressing and non-expressing *S. aureus*. For this purpose, liquid cultures of *S. aureus* RN4220 carrying ATc-inducible *drcAB* genes on a plasmid were spiked with defined concentrations of DAP, and supernatants were collected at different time points during growth to determine DAP concentrations by LC-MS/MS. After 24 h of growth, ATc-induced *drcAB* expression caused a highly significant loss (83.33%; *P* < 0.001) of the initial DAP concentration (Fig. 2; Fig. S1). The decrease was less pronounced (12.57%; *P* = 0.03) when *drcAB* was not induced, and DAP concentrations remained unaffected in *S. aureus* RN4220 harbouring the empty vector (Fig. 2; Fig. S1). These findings suggest that *drcAB* mediates the high-level DAP-R phenotype by inactivation of the antibiotic. The exact mechanism of DAP inactivation was further addressed in a recent separate study^21^.

### The *drc* locus is mobile and was acquired from another unknown species

**Fig. 3:**
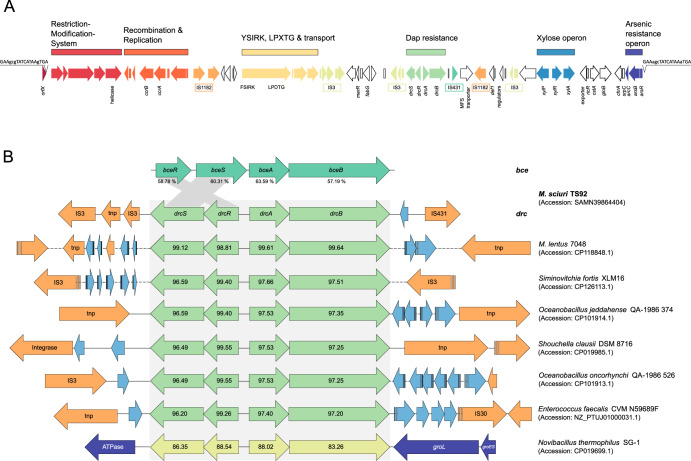
*drc* detection in bacterial genomes. **A** On the *M. sciuri* TS92 chromosome, the *drc* locus is associated with an SCC element displaying typical features of these genomic islands such as insertion into *orfX/rlmH*, presence of a pattern-1 recombination and replication locus, a restriction modification system, numerous insertion sequences (IS) as well as various resistance (*drc, ars*), metabolic (*xyl*) and adherence (LPXTG adhesin) genes as potential cargo. Imperfect tandem repeats are flanking the element. The drawing is not to scale. Total length is 67,813 bp. **B** Nucleotide BLAST searches at NCBI detect the *drc* locus in various Gram-positive species. Only selected hits are displayed. *drcRS*/*drcAB*-like genes are shown in green with nucleotide sequence percentage identities indicated. Blue arrows mark genes of general function and orange arrows highlight mobility-associated genes and elements. Except for dashed lines and arrows, the drawing is to scale.

We next inquired about the genetic origin of the *drc* genes in *M. sciuri* TS92. Sequencing of the *M. sciuri* TS92 genome revealed that the gene cluster is located in the immediate vicinity of an SCC element on the *M. sciuri* TS92 chromosome (Fig. 3A). Analysis of 211 representative *M. sciuri* genomes revealed that the locus was absent in all isolates, suggesting that the *drc* genes are not intrinsic to the *M. sciuri* species but have been acquired by strain TS92 through HGT from another organism. The flanking of the *drc* locus by mobile genetic elements (MGEs) such as insertion sequences (IS) on the *M. sciuri* TS92 chromosome further supports the idea of mobility of the gene cluster (Fig. 1A), raising the question of its presence in other bacteria as well as the microorganism from which these genes originate. The *drc* genes resemble a *bce* locus (encoding a putative bacitracin-sensing and ABC transporter system homologue) which is present in the core genomes of all *M. sciuri* isolates analysed to date (Fig. 3B, top). However, *drc* and *bce* are only ~60% identical at the nucleotide sequence level and differ in the orientation of the *drcRS/bceRS* regulatory genes, demonstrating that the two loci are only distantly related to each other (Fig. 3B, top). BLAST queries of the NCBI database, using the *drcB*_*TS92*_ nucleotide and/or DrcB_TS92_ protein sequences as inputs, identified the *drcRS/drcAB* locus in a number of Gram-positive bacteria (Table S4). Figure 3B illustrates organisation of the *drc* loci in representative species, with nucleotide sequence identities for *drcB* ranging from 97.20% to 99.64% (Fig. 3B, middle). *drc*-carrying isolates represented mainly bacterial species from soil and the environment (Table S4) but also included another *M. sciuri* strain and two *Mammaliicoccus lentus* isolates (formerly *Staphylococcus lentus*) from the Staphylococcaceae family (Table 1). In addition to staphylococci, we also found complete *drc* loci in three different *Enterococcus faecalis* isolates, including a strain that had been tested for DAP susceptibility and was found to be DAP-resistant^24^ (Table 1). The *drc*-carrying staphylococci and enterococci were of animal origin and were collected during resistance surveillance studies (Table 1). The *drc* loci in the various strains and species are flanked by different IS, suggesting mobility and acquisition via HGT (Table S4). Accordingly, these species are unlikely to represent the genetic origin of the *drc* locus. An exception is *Novibacillus thermophilus* strain SG-1 (*drcB* nucleotide sequence identity 83.26%; Fig. 3B, bottom). Here, the flanking MGEs are missing, and the *drc*-like gene cluster appears to be embedded within the core genome, which made this soil bacterium a promising candidate as the origin species of the *drc* locus. However, subcloning of the *drcAB-*like_SG-1_ genes onto a vector and expression in *B. subtilis* did not result in high-level DAP resistance (Fig. 1B), suggesting that the gene cluster present in *N. thermophilus* SG-1 is not the immediate genetic origin of the *drc* locus. However, more research is required to elucidate the evolutionary relationships between the various *bce/drc*-like loci as well as the genetic source of *drc*. Together, the data strongly suggest that the high-level DAP-R-mediating *drc* locus is mobile and is currently circulating in Gram-positive bacteria from soil and the environment, but also in commensal staphylococcal and enterococcal species from warm-blooded hosts such as *M. sciuri*, *M. lentus* as well as *E. faecalis*.

**Table 1:** Detection of the *drc* locus (*drcRS/drcAB*) in staphylococci and enterococci

Species Strain	Isolate source; Geographic region; Collection date	Accession BioProject BioSample	DAP MIC (µg/mL)	References	Alignments with *drcRS/drcAB*_TS92%_ nucleotide identity (Gaps)	MGE association of *drc*
*Mammaliicoccus sciuri* TS92	Pig farm, surface dust; Germany; 2013	PP236779 PRJNA1074542 SAMN39864404	64	This study	-	IS3 - *drc* - IS*431mec* (SCC element)
*Mammaliicoccus sciuri* SNUC 5594	Dairy cow, sublinical mastitis; Canada, Quebec; 2007	PZGV01000123.1 PRJNA342349 SAMN06173142	n.d.	^63^	99.98% (0)	IS3 - *drc* - IS*431mec*
*Mammaliicoccus lentus* *7048*	*Camelus dromedarius*, nose; Algeria, M’sila; 2021	NZ_CP118848.1 PRJNA937407 SAMN33407027	n.d.	^64^	99.64% (0)	n.d. - *drc* - ISL3 (family)
*Mammaliicoccus lentus* C9	Not specified; United Kingdom; 2016	NZ_JAAQRX010000032 PRJNA610046 SAMN14273045	n.d.	Direct submission	99.28% (0)	n.d. - *drc* - IS*431mec*
*Enterococcus faecalis* CVM N59689F	Market swine; USA, Iowa; 2014	PTUJ01000031.1 PRJNA292669 SAMN07982045	>16	^24^	97.40% (0)	Tn3 family recombinase - *drc*
*Enterococcus faecalis* FSIS 12032216	Dairy cow; USA, Texas; 2020	AAXCZN010000013.1 PRJNA292669 SAMN15856852	n.d.	Direct submission	97.23% (0)	Tn3 family recombinase - *drc* (on plasmid?)
*Enterococcus faecalis* FSIS 11808951	Cattle (steer); USA, Pennsylvania; 2018	AAXEJZ010000069.1 PRJNA292669 SAMN10482714	n.d.	Direct submission	97.21% (0)	Tn3 family recombinase - *drc*

*drcRS/drcAB* loci were identified by performing BLAST queries of the NCBI database (https://blast.ncbi.nlm.nih.gov/Blast.cgi), using the *drcB*_*TS92*_ nucleotide and/or DrcB_TS92_ protein sequences as inputs. The complete hit list is shown in Table S4. n.d. not determined, MGE mobile genetic element, IS insertion sequence, SCC staphylococcal cassette chromosome, Tn transposon.

## Discussion

Since the introduction of penicillin into clinical practice almost a century ago, there has been an ongoing arms race between antibiotic discovery and the emergence of bacterial resistance. One lesson learned over the years is that bacteria will develop resistance to any antibacterial agent sooner or later, and the finding of a novel DAP-R mechanism reported in this study is yet another powerful confirmation of this fact. Bacteria develop resistance either through adaptive mutations or via the uptake of foreign DNA by HGT. The chromosomal mutations previously shown to be associated with DAP-R are unlikely to spread to other bacteria, while the transmissible DAP-R mechanism identified here bears the risk of entry into pathogenic species and subsequent clonal spread of high-level DAP-R strains (including MRSA and VRE), which would seriously jeopardise the clinical applicability of DAP as a reserve antibiotic. But how likely is such a scenario? *drc* is probably arising from an environmental microorganism and to understand how an AMR determinant can gradually gain access to pathogens, it is worth considering the origins of AMR and the factors that drive its evolution.

In fact, most of our currently used antibiotics (including DAP) are derivatives of natural compounds. Particularly, soil microorganisms are a rich source of both antibiotics and AMR genes, with the evolution of AMR dating back long before humans began to exploit antibiotics for treating infections^25–28^. Hence, we can expect the presence of AMR genes in nature, and the existence of resistance determinants to DAP does not seem to be an exception to this rule. For example, phenotypic resistance to DAP and other reserve antibiotics was previously found in bacteria of the genera *Streptomyces, Paenibacillus* and *Actinomyces* from (prehistoric) soil samples^28,29^. These strains showed various DAP-inactivating enzymatic activities (including DAP hydrolysis), which, however, could not be associated with genetic elements at that time^28,30^. The authors of these studies postulated the possibility of transfer of DAP-inactivating enzymes to sensitive species^30^, an assumption that is now corroborated by our report.

Whether a resistance determinant from the soil resistome will become clinically relevant depends on multiple factors, including the mobilisation of the AMR genes from the ancestral genome, contact and access to host-associated bacteria, the expression of the AMR genes in the new host, and finally the selection of AMR-carrying genotypes^26^. The *drc* locus is the first known genetic element associated with transmissible DAP-R, and its detection in commensal staphylococci and enterococci suggest that *drc* has successfully overcome most of these evolutionary bottlenecks.

Mobilisation of AMR genes from an ancestral species is often achieved via accumulation of IS elements which not only enable movement of genetic material within the host genome, but also promote the transfer to other bacteria once the AMR genes become part of an autonomous mobile genetic element (*e.g*. plasmid, phage, genomic island, etc.)^31^. Flanking of the *drc* locus by IS in different bacterial species, as shown in this study, indicates that the gene cluster has successfully attained mobility and is circulating among Gram-positive bacteria, suggesting that the first hurdle in AMR evolution has been taken (Fig. 3, Table S4).

Another bottleneck in clinical AMR development is that AMR genes from environmental microorganisms must gain access to pathogenic species. This requires donors and recipients to come into close physical contact, which is often challenging as pathogenic and environmental species rarely share the same ecological niche. In this respect, *M. sciuri* may play a crucial role in *drc* acquisition and spread. Due to its dual lifestyle as a soil bacterium and animal commensal, *M. sciuri* comes into contact with both the soil resistome and commensal bacteria associated with warm-blooded hosts, making *M. sciuri* a gateway species through which novel AMR genes can enter commensals^19^. *M. sciuri* effectively exchanges genetic material with other staphylococci, including *S. aureus*, and was shown in the past to substantially contribute to staphylococcal AMR development^19^. For example, *M. sciuri* represents the genetic origin of the methicillin resistance-mediating *mecA* gene, which was horizontally transferred into *S. aureus* and led to the emergence of MRSA^32^. More recently, the species was described as the donor of a transferable trimethoprim resistance gene^33^, and the ancestors of the mobile SCC elements (on which *mec* genes are located on) also originally evolved within *M. sciuri* before successfully spreading to other staphylococci^34^. SCCs are mobile replicative chromosomal elements that represent hotspots of recombination prone to integrate newly acquired genetic material^35–37^. It is tempting to speculate that the *drc* locus inserted next to SCC_TS92_ might be mobilised together with the element, which would bear the risk of a *drc* transfer into other staphylococci, including *S. aureus*. Our experiments show that the *drc* locus is fully functional in various *S. aureus* backgrounds, including *S. aureus* USA300 LAC* which belongs to the highly pathogenic community-acquired MRSA lineage USA300^38,39^. The high levels of DAP-R displayed in this isolate indicate that *drc* acquisition by MRSA represents a potential danger that would limit clinical treatment options of MRSA infections (Fig. 1B). Our database searches show that *drc* has already found its way from environmental bacteria to commensals (Table S4). For example, the presence of *drc* in two *M. sciuri* and two *M. lentus* strains indicates that the resistance determinant is beginning to spread successfully within the Staphylococcaceae family (Table 1). The association of *drc* with IS*431mec* (Table 1), an IS which is known to integrate into SCC elements, may facilitate the mobility of *drc* and increase the risk of future transmission to other *Staphylococcus* species, including *S. aureus* and MRSA. Also, the detection of *drc* in three *E. faecalis* isolates is an alarming signal (Table 1). *E. faecalis* is a common opportunistic pathogen characterised by pronounced intrinsic and acquired AMR, with DAP playing an important role as a last-line therapy^40^. In the three *E. faecalis* genomes, *drc* is associated with a Tn3-like recombinase, and in isolate *E. faecalis* FSIS 12032216 the resistance determinant appears likely to be located on an (integrated) plasmid, indicating the potential for further *drc* dissemination among enterococci. Remarkably, *E. faecalis* CVM N59689F, which was recovered in an antimicrobial resistance monitoring study, was tested for DAP susceptibility in this report^24^. The isolate showed a DAP MIC of >16 µg/mL, which at that time could not be assigned to a known genetic AMR mechanism^24^. In the light of our findings, it is reasonable to suggest that the DAP-R phenotype in this isolate is caused by *drc*. Finally, the high level of DAP-R displayed upon *drc* transfer into *B. subtilis* suggests that the gene cluster is functional in various Gram-positive host bacteria.

Our study shows that the *drc* locus has obviously arrived in staphylococci and enterococci. However, whether or not such isolates will prevail and spread further depends on their selection which is another critical step in AMR development. The *drc*-bearing staphylococci and enterococci identified in this study were all of animal origin and collected during AMR surveillance studies (Table 1). DAP is only rarely used in veterinary medicine and not approved for food-producing animals which might explain why *drc* detection is still rare. However, the situation could well change as soon as *drc*-carrying staphylococci and enterococci are transmitted to humans. Staphylococci and enterococci are known to be readily exchanged among animals and humans^41,42^, and in human medicine, DAP consumption is currently steadily growing^43^. The increasing DAP selection pressure could then favour *drc*-bearing genotypes and promote the clonal spread of high-level DAP-R strains. Such predictions are not particularly bold, as we have already seen similar scenarios in other AMR bacteria. Worrisome examples of the recent past are carbapenem- and colistin-resistant Gram-negative bacteria, whose global spread has been accelerated by the emergence of mobile resistance determinants and the increasing use of these drugs as reserve antibiotics^44,45^. It is noteworthy that many of these AMR determinants were initially also detected in livestock, suggesting that this is an important environment for AMR emergence^26^. Therefore, the detection of a mobile DAP-R mechanism in livestock-associated commensal bacteria is alarming and could mark the development of DAP-R on a larger scale. A key factor in avoiding this will be the reduction of DAP selection pressure through the rational and highly restrictive use of the antibiotic.

Finally, with the discovery of the *drc* locus we are expanding the large class of known BceAB-type transporters by a new member with striking properties. Bce modules are membrane-associated protein complexes that Gram-positive bacteria have evolved to resist attacks by antimicrobial peptides and cell-wall-targeting antibiotics, with the bacitracin resistance-mediating Bce module from *B. subtilis* representing the prototype system^23^. Bce modules consist of an ATP-binding cassette (ABC) transporter and a two-component system which coevolved and form together a protein complex to sense and detoxify substrates^46^. Recent structural and functional data suggest that the bacitracin Bce module from *B. subtilis* does not transport substrates across membranes^47,48^, but rather mediates protection of cell wall synthesis by freeing bacitracin targets (*i.e.*, lipid II cycle intermediates) from the antibiotic^49^. Remarkably, this mechanism does not involve inactivation of bacitracin. Association of a Bce transporter with substrate-inactivating activity, as shown for DrcAB in our report, is indeed an unexpected and surprising finding. Using LC-MS/MS, we demonstrate that DAP levels decrease after 24 h when DrcAB is expressed (Fig. 2, Fig. S1). The fate of DAP and the exact chemical nature of its inactivation was recently addressed in a parallel separate study, in which we employed a high resolution mass spectrometry (HRMS) approach to obtain structure information on DrcAB-generated DAP products^21^. The analyses revealed that DAP is structurally altered by a two-step process catalysing first the transfer of dehydroalanine (DHA) to the kynurenine residue present in the DAP polypeptide ring, followed by ring opening of the DAP core through hydrolysis of the ester bond between kynurenine and threonine, indicating that DrcAB has both transferase and hydrolase activity to chemically modify and inactivate DAP^21^. Hydrolysis of the DAP core, including cleavage between kynurenine and threonine, has been reported before in soil-borne microorganisms^30^. However, the DrcAB-mediated transfer of DHA to kynurenine prior to hydrolysis, is an interesting new finding that warrants further in-depth functional and structural studies^21^.

Together, this is the first report of a horizontally acquired daptomycin resistance mechanism. The cross-sectoral detection of *drc* in soil-borne microorganisms as well as in animal and human commensals advocates to include low and apathogenic bacteria from these habitats in existing AMR monitoring systems. By pursuing such an One Health approach, novel AMR determinants against critically important antibiotics could be identified at an early stage, before they become a clinical problem.

## Methods

### Strains

Bacterial strains and growth conditions are listed in Table S1. Bacteria were grown in Muller-Hinton broth (MH) (Carl Roth GmbH + Co. KG, Karlsruhe, Germany) and over-night cultures supplemented with antibiotics when required. For DAP (Cayman Chemical, Michigan, USA) MIC determination, a serial dilution from 256 µg/mL onwards was prepared in MH supplemented with 50 µg/mL CaCl_2_. For transcription profiling experiments, bacteria were exposed to 128 µg/mL DAP for 30 min. All strains, except bacilli (30 °C), were incubated at 37 °C.

### Recombinant plasmid and strain generation

Recombinant plasmids (Table S1) were generated by in vitro *E. coli* cloning method^50^, employing shuttle vector pCG248 as backbone and *E. coli* DC10B as host. Utilised primers are listed in Table S2. After recovery from *E. coli*, recombinant plasmids were transformed into *S. aureus* or *B. subtilis* via electroporation.

### Whole genome sequencing of *M. sciuri* TS92

Genomic DNA was extracted from isolate TS92 and subjected to next generation sequencing using an Illumina MiSeq platform (Illumina, San Diego, USA). Paired-end sequences (2 × 250 bp) were generated and processed using an in-house pipeline for quality control, including removal of adapter sequences and minor contaminants. Because short-read sequencing was insufficient to completely reconstruct the full genome of TS92, additional long-read sequencing was therefore performed on the Nanopore platform (Oxford Nanopore Technologies Ltd, Oxford, UK) and combined with the Illumina data for genomic assembly.

### Genomic reconstruction and annotation

Both, short- and long-read sequences were utilised for the hybrid assembly of TS92 using the unicycler pipeline (v0.4.4), resulting in full genomic reconstruction of the bacterial chromosomal sequence^51^. Completeness was visually assessed via the Bandage software (v0.8.1)^52^. Species-specific annotation was performed using Prokka (v1.14.6)^53^. Additional elements of interest were further assessed via the ABRicate software (v1.0.1) (https://github.com/tseemann/abricate). This included profiling of AMR genes via the MEGARes database^54^ and IS elements through the ISfinder sequences^55^. Genomic comparisons of the annotated SCC region were performed via Easyfig (v2.2.2)^56^.

### Sample preparation for transcription profiling and RNA extraction

An overnight culture of *M. sciuri* TS92 was diluted in fresh MH to an optical density at 600 nm (OD_600_) of 0.05 and grown to OD_600_ 0.5. The culture was split into 10 mL each aliquots and incubated in the absence and presence of 128 µg/mL DAP for 30 min. Subsequently, total RNA extraction was performed as described previously^57^.

### Transcription profiling

RNA was isolated from three biological replicates. RNA quality was checked using a 2100 Bioanalyzer with the RNA 6000 Nano Kit (Agilent Technologies, Santa Clara, USA). cDNA libraries suitable for sequencing were prepared from 1000 ng of total RNA after DNase I treatment (Thermo Fisher, Darmstadt, Germany). Except for one control sample, all RNA samples were rRNA depleted by RiboPOOLs (siTOOLs Biotech GmbH, Planegg/Martinsried, Germany) and subsequently fragmented by incubation with Mg^2+^ ions for 2 min 45 s using the NEBNext® Magnesium RNA fragmentation module (New England Biolabs, Frankfurt am Main, Germany). RNA samples were then treated with T4 polynucleotide kinase for phosphorylation/dephosphorylation as well as with RppH for decapping, followed by library preparation using the NEBNext® Multiplex Small RNA Library Prep Set (New England Biolabs). The number of PCR cycles was determined at 14 by qPCR and the elongation time was set to 15 s. Libraries were quantified by Qubit^TM^ dsDNA HS Assay Kit and Qubit^TM^ 3.0 Fluorometer (ThermoFisher), and quality was checked using 2100 Bioanalyzer with the High Sensitivity DNA Kit (Agilent Technologies) before pooling. Sequencing of pooled libraries, spiked with 5% PhiX control library, was performed at ~11–15 million reads per sample in single-end mode with 75 nucleotide read length on the NextSeq 500 platform (Illumina, San Diego, CA, USA). Demultiplexed FASTQ files were generated with bcl2fastq2 v2.20.0.422 (Illumina). Illumina reads were quality- and adapter-trimmed via Cutadapt v2.1^58^ using a cut-off Phred score of 20 in NextSeq mode, and reads without any remaining bases were discarded (command line parameters: --nextseq-trim=20 -m 1 -a AGATCGGAAGAGCACACGTCTGAACTCCAGTCAC). Afterwards, the pipeline READemption v0.4.5^59^ was applied to align all reads longer than 11 nt (-l 12) to our de novo assembled *M. sciuri* TS92 genome using segemehl v0.2.0^60^ with an accuracy cut-off of 95% (-a 95). READemption gene_quanti was used to quantify aligned reads overlapping genomic features by at least 10 nts (-o 10) on the sense strand (-a). Based on these counts, differential expression analysis comparing DAP-exposed samples to untreated controls was conducted via DESeq2^61^ version 1.24.0 with fold-change shrinkage applied by setting the parameter betaPrior to TRUE. Results of the analyses are shown in Table S3.

### MIC testing

To determine the minimal inhibitory concentration (MIC), a standard micro-broth dilution assay^62^ in MH medium supplemented with 50 µg/mL CaCl_2_ was performed, with DAP testing concentrations ranging from 0.25 µg/mL to 256 µg/mL. The cultures were adjusted to 5 × 10^5^ CFU/mL and transferred to a 96-well flat bottom microtiter plate (Greiner Bio-One GmbH, Frickenhausen, Germany). 100 ng/mL anhydrotetracycline (ATc) (Carl Roth GmbH + Co. KG, Karlsruhe, Germany) was added if required; ATc-containing plates were stored in the dark. Plates were incubated for 18 h at 37 °C (24 h at 30 °C for bacilli) under static conditions. Afterwards, the OD_595_ was measured to determine the MIC according to EUCAST guidelines.

### Daptomycin detection

To analyse the fate of DAP, bacterial cultures in MH broth were spiked with DAP equivalent to one quarter of the MIC of the respective strain tested, i.e. 0.25 μg/mL for *S. aureus* RN4220_empty vector control and 16 μg/mL for *S. aureus* RN4220_*P*_*xyl/tet*_*-drcAB*. *drcAB* expression was induced by adding 100 ng/mL ATc. The cultures were then incubated at 200 rpm and 37 °C, and supernatants were collected after 0, 0.5, 6, and 24 h. The samples were centrifuged, sterile-filtered, and stored at −80 °C until further analysis. DAP concentrations in the supernatants were determined by liquid chromatography–tandem mass spectrometry/ mass spectrometry (LC-MS/MS) as recently described^22^.

### Figures and statistics

Figures and statistics were created using GraphPad Prism 10 (Version 10.1.1). Supplement figures were created using Microsoft Excel. For statistical analysis One-Way ANOVA with Dunnett multiple comparisons test was performed (alpha level: 0.05) using biological replicates (*n* = 2) with technical replicates (*n* = 5) each. ANOVA was used because the mean values of more than two groups were tested for their variances. Samples were always compared to control sample (*t* = 0) thus Dunnett comparisons test was chosen for multiple comparison.

## Supplementary information

Supplementary Information

## Data Availability

Genome and RNA-Seq data are deposited at NCBI and are available under BioProject PRJNA1054011 (WGS), PRJNA1074542; SAMN39864404 (M. sciuri TS92 genome) and GEO GSE255509 (RNA-Seq). The nucleotide sequence of the drc locus is accessible under GenBank accession number PP236779.
