# Supplementary material for: Emergence of transferable daptomycin resistance in Gram-positive bacteria
Source: NPJ Antimicrob Resist. 2025 Apr 26;3:33. doi: 10.1038/s44259-025-00109-z (PMC12033364; doi:10.1038/s44259-025-00109-z)
Supplement: Supplementary file 1 — Supplementary Information [file 44259_2025_109_MOESM1_ESM.pdf]

Supplementary Appendix

Emergence of transferable daptomycin resistance in Gram-positive bacteria

Tessa Marciniak[1+], Lukas Kirchner[2+], Silver A. Wolf[3], Birgit Walther[4], Thorsten Bischler[5], Justin Nyasinga[6], Revathi Gunturu[6], Torsten Semmler[3], Tom Grafenhan[5], Andrew Whitelaw[7], Oliver Scherf-Clavel[8], Ulrike Holzgrabe[2], Wilma Ziebuhr[1*]

[+] Authors contributed equally to this work

[*] Corresponding author: Wilma Ziebuhr; wilma.ziebuhr@uni-wuerzburg.de; +49 931 3182154

[1]Institute of Molecular Infection Biology, University of Wurzburg, Wurzburg, Germany

[2]Institute for Pharmacy and Food Chemistry, University of Wurzburg, Wurzburg, Germany

[3]Genome Competence Centre (MF1), Robert Koch Institute, Berlin, Germany

[4]Microbiological Risks (II 1.4), German Environment Agency, Berlin, Germany

[5]Core Unit Systems Medicine, University of Wurzburg, Wurzburg, Germany

[6]The Aga Khan University Hospital, Nairobi, Kenya

[7]Division of Medical Microbiology, Stellenbosch University, Cape Town and National Health Laboratory Service, Tygerberg Hospital, Cape Town, South Africa

[8]Department of Pharmacy, Clinical Pharmacy and Pharmacotherapy, Ludwig-Maximilians-University, Munich, Germany

Table of Content

Table S1.	Bacterial strains and plasmids used in this study.	p. 1
Table S2.	List of oligonucleotides used in this study.	p. 3
Table S3.	Differentially expressed genes in *Mammaliicoccus sciuri* TS92 upon daptomycin exposure (128 µg/ml).	p. 4
Figure S1.	Representative normalized multiple-reaction-monitoring (MRM) chromatograms obtained from LC-MS/MS measurements of DAP in supernatant.	p. 8
Table S4.	Detection of the drc locus (drcRS/drcAB) in various bacterial strains and species.	p. 9
Supplementary References		p. 13

Table S1. Bacterial strains and plasmids used in this study. Bacteria were grown in Muller-Hinton broth (MH) (Carl Roth GmbH + Co. KG, Karlsruhe, Germany) supplemented with antibiotics when required. For DAP application (Cayman Chemical, Michigan, USA), MH was supplemented with 50 µg/mL $CaCl_2$. All strains, except bacilli (30 °C), were incubated at 37 °C. *M.* – *Mammaliicoccus*, *E.* – *Escherichia*, *B.* – *Bacillus*, *S.* – *Staphylococcus*, ATc – Anhydrotetracycline.

**Bacterial strains or plasmids**	**Description**	**Reference**
**Bacterial strains**		
M. sciuri TS92	Isolated from a dust sample collected at a pig farm in Germany; daptomycin resistant	[1]
E. coli DC10B	*E. coli* DH10B Δ*dcm* (cytosine methylase deficient). Cloning host for shuttle plasmids enabling direct transformation of recombinant vectors into *S. aureus.*	[2]
B. subtilis 168	DSM 402, Wildtype strain	[3]
B. subtilis 168_empty vector control (TM016)	*B. subtilis* 168 carrying plasmid pCG248	This study
B. subtilis 168_$P_{xyl/tet}$-*drcAB* (TM018)	*B. subtilis* 168 carrying plasmid pTM04	This study
B. subtilis 168_*drcRS*/*drcAB*	*B. subtilis* 168 carrying plasmid pTM08	This study
S. aureus RN4220	Restriction-deficient derivative of 8325-4, cloning host	[4]
S. aureus USA300 LAC*	Community-associated methicillin-resistant *S. aureus* USA300-0114 cured from plasmid pUSA03. Erythromycin sensitive.	[5,6]
S. aureus RN4220_empty vector control	*S. aureus* RN4220 carrying plasmid pCG248	This study
S. aureus RN4220_$P_{xyl/tet}$-*drcAB* (TM008)	*S. aureus* RN4220 carrying plasmid pTM04	This study
S. aureus RN4220 $P_{xyl/tet}$-*drcA*B* (TM012)	*S. aureus* RN4220 carrying plasmid pTM05	This study
S. aureus RN4220_$P_{xyl/tet}$-*drcAB** (TM014)	*S. aureus* RN4220 carrying plasmid pTM06	This study
S. aureus RN4220_*drcRS*/*drcAB* (TM021)	*S. aureus* RN4220 carrying plasmid pTM08	This study
S. aureus RN4220_*drcRS**/*drcAB* (TM027)	*S. aureus* RN4220 carrying plasmid pTM12	This study

**Bacterial strains or plasmids**	**Description**	**Reference**
**Bacterial strains**		
S. aureus RN4220_*drcR*S/drcAB* (TM031)	*S. aureus* RN4220 carrying plasmid pTM13	This study
S. aureus USA300 LAC*_empty vector control (TM180)	*S. aureus* USA300 LAC* carrying plasmid pCG248	This study
S. aureus USA300 LAC*_$P_{xyl/tet}$-*drcAB* (TM178)	*S. aureus* USA300 LAC* carrying plasmid pTM04	This study
S. aureus USA300 LAC*_*drcRS*/*drcAB* (TM179)	*S. aureus* USA300 LAC* carrying plasmid pTM08	This study
**Plasmids**		
pCG248	Gram(-)/ Gram(+) shuttle plasmid harbouring the $P_{xyl/tet}$ promoter for ATc-controlled gene expression; empty control vector	[7]
pTM04	pCG248 with *drcAB* from *M. sciuri* TS92 under control of ATc-inducible $P_{xyl/tet}$ promotor	This study
pTM05	pTM04 with point mutation (T86A) in *drcA* causing an early stop codon	This study
pTM06	pTM04 with point mutation (A127T) in *drcB* causing an early stop codon	This study
pTM08	pCG248 backbone (lacking $P_{xyl/tet}$) with native *drcRS/drcAB* locus from M. *sciuri* TS92	This study
pTM12	pTM08 with early stop codon mutation in *drcS* (A259T)	This study
pTM13	pTM08 with early stop codon mutation in *drcR* (G167T)	This study

Table S2. List of oligonucleotides used in this study. Nucleotides in small letters indicate changes to create a stop codon in the sequence.

**Plasmid**	**Template**	**Name**	**Sequence**
**Creating new plasmids**			
pTM04	pCG248	GM192	GAATTCAGGCGCGCCTATTC
		GM193	AGATCTGTTAACGGTACCATCA
		GM194	GTACCGTTAACAGATCTGAATGGAGGATTTAAATATGAA
		GM195	GGCGCGCCTGAATTCTTATTTGTTTACAATGCGATTG
pTM05	pTM04	FW107	GGCGAGTTACATGATCCCCCATGTTGT
		FW108	GGGGGATCATGTAACTCGCCTTGAT
		TM006	GGAGGATATAGACTaAACGGTCAT
		TM007	GACCGTTtAGTCTATATCCTCCAA
pTM06	pTM04	FW107	
		FW108	
		TM008	GCAATATAGCGAAtAAATTCAGGAAA
		TM009	CCTGAATTTaTTCGCTATATTGCAA
pTM08	pCG248	TM023	CGGGTACCGAGCTCGAATT
		TM029	CCTTTTTCCGTGATGGTAACTT
		TM030	GGAAGTTACCATCACGGAAAAAGGGCGATATTCCCCTTTCAAAA
		TM031	CCTGAATTCGAGCTCGGTACCCGGGAATACATAACAAAACACCT
pTM12	pTM08	FW107	
		FW108	
		TM034	CGCAACTCTGCCTtAACCGCGAAA
		TM035	GCGGTTaAGGCAGAGTTGCGAGAAA
pTM13	pTM08	FW107’	GTTACATGATCCCCCATGTT
		FW108’	GGATCATGTAACTCGCCTTGAT
		TM032’	GCCATaGTTTGACGGATTTTAC
		TM033’	CCGTCAAACtATGGCAAATTGAT
**General PCR**			
		pCG248-MCS_rev	TCTCGAAAATAATAGAGGGAAAATCAG
		pRAB11-MCS_for	GCAGCTCTAATGCGCTGTTAATCAC

Table S3. Differentially expressed genes in *Mammaliicoccus sciuri* TS92 upon daptomycin exposure (128 µg/mL). Comparison: Treated sample (128 µg/mL) versus untreated control (0 µg/mL). Upregulated genes upon daptomycin exposure (log2_fold change >2; *p* <0.05) are highlighted in green. Downregulated genes upon daptomycin exposure (log2_fold change < -2; *p* <0.05) are highlighted in blue.

Genome	Source	Type	Start	End	Strand	Locus_tag	Name	GeneID	Product	Note	TS92_0_replicate_1	TS92_0_replicate_2	TS92_0_replicate_3	TS92_128_replicate_1	TS92_128_replicate_2	TS92_128_replicate_3	TS92_32_replicate_1	TS92_32_replicate_2	TS92_32_replicate_3	TS92_0_replicate_1_normalized	TS92_0_replicate_2_normalized	TS92_0_replicate_3_normalized	TS92_128_replicate_1_normalized	TS92_128_replicate_2_normalized	TS92_128_replicate_3_normalized	TS92_32_replicate_1_normalized	TS92_32_replicate_2_normalized	TS92_32_replicate_3_normalized	baseMean	log2FoldChange	lfcSE	stat	pvalue	padj
TS92_Nanopo	.	CDS	83120	83887	+	TS92_00079	drcA	NA	ABC transporter ATP-binding protein%2C involved%0Ain daptomycin resistance	NA	297	368	563	46796	58096	52889	60097	87373	99761	205.380187	243.50937	364.769593	67001.8235	65983.5189	66690.7149	95993.727	89021.4718	105194.691	54522.1785	7.87352181	0.19374102	40.6394164	0	0
TS92_Nanopo	.	CDS	83874	85799	+	TS92_00080	drcB	NA	ABC transporter permease protein%2C involved in%0Adaptomycin resistance	NA	399	472	764	42252	52418	52553	96756	140096	161240	275.914796	312.327235	494.998169	60495.7913	59534.634	66267.0336	154549.629	142739.2	170022.274	72743.5335	7.35886457	0.20339253	36.1806039	1.229E-286	4.569E-284
TS92_Nanopo	.	CDS	1679679	1679843	-	TS92_01747	TS92_01747	NA	hypothetical protein	NA	181	202	174	8478	17882	10286	15029	22052	24882	125.164356	133.665469	112.735185	12138.6755	20309.7853	12970.196	24006.019	22468.0564	26237.2502	13166.8386	6.86792979	0.20713157	33.1573296	4.441E-241	1.101E-238
TS92_Nanopo	.	CDS	463982	464335	+	TS92_00434	TS92_00434	NA	hypothetical protein	NA	32	47	41	1886	3395	2324	3462	5943	4903	22.128505	31.1003815	26.5640379	2700.34702	3855.92892	2930.46232	5529.89805	6055.12695	5170.05215	2924.62315	6.81483356	0.21266748	32.0445492	2.615E-225	4.86E-223
TS92_Nanopo	.	CDS	2422802	2423458	-	TS92_02528	TS92_02528	NA	hypothetical protein	NA	38	55	29	1769	2821	2148	2521	3834	4046	26.2775996	36.3940634	18.7891975	2532.82814	3203.99867	2708.53402	4026.82639	3906.33631	4266.37385	2302.92869	6.62837078	0.20462429	32.3928831	3.457E-230	7.344E-228
TS92_Nanopo	.	CDS	2378378	2378626	+	TS92_02480	TS92_02480	NA	hypothetical protein	NA	243	229	275	6437	9078	8440	15329	23134	23469	168.038335	151.531646	178.173425	9216.40179	10310.4927	10642.4707	24485.2129	23570.4706	24747.2882	11496.6756	5.90048782	0.12440441	47.429891	0	0
TS92_Nanopo	.	CDS	554936	555334	+	TS92_00534	TS92_00534	NA	hypothetical protein	NA	142	179	165	3942	7220	5016	6229	9437	15466	98.1952407	118.446134	106.904055	5644.09754	8200.23765	6324.95653	9949.66347	9615.04846	16308.388	6262.8819	5.89018024	0.23286711	25.2941699	3.703E-141	2.824E-139
TS92_Nanopo	.	CDS	2451230	2451931	-	TS92_02559	TS92_02559	NA	putative secreted protease inhibitor	NA	469	697	626	14461	24472	17067	19293	35890	34785	324.320901	461.21204	405.587505	20705.0468	27794.4897	21520.7403	30816.9622	36567.1388	36679.6378	19475.0151	5.83719197	0.17744218	32.8963053	2.482E-237	5.678E-235
TS92_Nanopo	.	CDS	2813973	2815604	+	TS92_02936	TS92_02936	NA	hypothetical protein	NA	1323	1553	1918	42777	47519	46482	60994	93738	110486	914.875377	1027.63601	1242.67865	61247.4785	53970.5115	58611.7683	97426.5169	95506.5606	116503.851	54050.2085	5.74148422	0.15195146	37.7849898	0	0
TS92_Nanopo	.	CDS	2008290	2008415	+	TS92_02094	TS92_02094	NA	hypothetical protein	NA	29	54	56	1166	1638	1319	1645	4404	3252	20.0539576	35.7323532	36.2825883	1669.46163	1860.38633	1663.20129	2627.58009	4487.09054	3429.12698	1758.76842	5.72987989	0.24701238	23.1967319	4.913E-119	2.982E-117
TS92_Nanopo	.	CDS	2135996	2137636	-	TS92_02211	oppA	NA	oligopeptide ABC superfamily ATP binding%0Acassette transporter%2C binding protein	NA	804	609	1121	19472	27343	22942	20072	25422	31385	555.978687	402.981539	726.29967	27879.7228	31055.2767	28928.8582	32061.2691	25901.6384	33094.4497	20067.3861	5.65385707	0.2025465	27.9138722	1.811E-171	2.154E-169
TS92_Nanopo	.	CDS	2793787	2794683	-	TS92_02911	TS92_02911	NA	NAD dependent epimerase/dehydratase%0Afamilyprotein	NA	436	393	367	6696	8807	8070	10940	14916	17226	301.50088	260.052126	237.780534	9587.23418	10002.6999	10175.9169	17474.6056	15197.4211	18164.2501	9044.60681	5.19842735	0.13520573	38.4482782	0	0
TS92_Nanopo	.	CDS	541295	541834	+	TS92_00517	TS92_00517	NA	hypothetical protein	NA	294	253	338	4835	5813	5952	6358	9442	9994	203.305639	167.412692	218.991337	6922.68179	6602.2135	7505.21158	10155.7169	9620.1428	10538.3441	5770.4467	5.13508815	0.13578629	37.8174265	0	0
TS92_Nanopo	.	CDS	2105601	2106422	-	TS92_02183	terC	NA	membrane protein TerC	NA	1748	1839	1603	30584	33023	34032	21600	20513	33198	1208.76958	1216.88514	1038.58909	43789.7207	37506.4332	42912.863	34501.9635	20900.02	35006.1985	24231.2714	5.12065826	0.19853708	25.7919495	1.092E-146	9.019E-145
TS92_Nanopo	.	CDS	197387	197878	+	TS92_00192	TS92_00192	NA	Tryptophan-rich transmembrane signaling protein	NA	77	88	129	1731	1694	2046	2615	4432	4638	53.2467151	58.2305015	83.5795338	2478.4203	1923.98928	2579.91648	4176.97383	4515.61882	4890.61837	2306.73265	5.11396462	0.19552812	26.1546255	8.731E-151	7.868E-149
TS92_Nanopo	.	CDS	2134329	2135951	-	TS92_02210	opp3A_1	NA	oligopeptide ABC superfamily ATP binding%0Acassette transporter%2C binding protein%2	NA	1060	911	1365	16892	25243	19764	27090	38805	39472	733.006727	602.818033	884.38809	24185.7168	28670.1661	24921.5393	43271.2126	39537.1363	41621.925	22714.2121	5.10305811	0.15798733	32.300426	6.899E-229	1.368E-226
TS92_Nanopo	.	CDS	2622543	2623325	-	TS92_02731	TS92_02731	NA	alpha/beta hydrolase	NA	280	351	372	4762	7260	6058	8913	13125	15029	193.624418	232.260296	241.020051	6818.16146	8245.66833	7638.87294	14236.8519	13372.6302	15847.5859	7425.18617	5.06519332	0.14467466	35.0109217	1.535E-268	4.564E-266
TS92_Nanopo	.	CDS	344646	345584	+	TS92_00326	TS92_00326	NA	hypothetical protein	NA	267	313	304	4132	6508	5175	6490	11798	10811	184.634713	207.115307	196.962622	5916.13674	7391.57155	6525.44858	10366.5622	12020.5936	11399.8437	6023.20767	5.0523544	0.13890062	36.3738793	1.102E-289	4.682E-287
TS92_Nanopo	.	CDS	1630768	1631751	+	TS92_01700	ypuA	NA	putative protein YpuA	NA	947	1553	814	17510	25006	15860	23488	46094	32998	654.865444	1027.63601	527.393337	25070.5601	28400.9893	19998.7661	37517.6907	46963.6583	34795.3051	21661.8738	4.99216032	0.24026082	20.7780875	6.8327E-96	2.6737E-94
TS92_Nanopo	.	CDS	1588171	1588512	+	TS92_01658	TS92_01658	NA	hypothetical protein	NA	71	96	125	1189	1886	1595	863	1476	1756	49.0976204	63.5241835	80.9879204	1702.39269	2142.05654	2011.22521	1378.48123	1503.84778	1851.64421	1198.13971	4.87163484	0.20269008	24.0348952	1.201E-127	7.765E-126
TS92_Nanopo	.	CDS	2239640	2240056	+	TS92_02318	osmC	NA	osmotically inducible protein OsmC	NA	522	601	676	7375	10779	8968	10752	18902	19777	360.971237	397.687857	437.982673	10559.4164	12242.4324	11308.2556	17174.3107	19258.6252	20854.1957	10288.2086	4.81350264	0.13582384	35.4393067	4.239E-275	1.401E-272
TS92_Nanopo	.	CDS	876546	878234	-	TS92_00883	ilvD	NA	putative dihydroxy-acid dehydratase	NA	50	40	44	670	656	701	544	834	734	34.575789	26.4684098	28.507748	959.296132	745.063144	883.930329	868.938341	849.735129	773.978845	574.499318	4.81113732	0.19213943	25.0398228	2.254E-138	1.676E-136
TS92_Nanopo	.	CDS	2256684	2257061	-	TS92_02338	TS92_02338	NA	hypothetical protein	NA	333	409	365	4704	6973	4895	2552	4629	3754	230.274755	270.63949	236.484727	6735.11792	7919.7032	6172.38083	4076.3431	4716.33563	3958.46946	3812.86101	4.79642112	0.15011281	31.9521109	5.05E-224	8.834E-222
TS92_Nanopo	.	CDS	1678957	1679160	-	TS92_01745	xynA	NA	TM2 domain containing membrane protein+B7201	NA	908	1028	1082	13204	15160	16524	30412	34740	49349	627.896328	680.238131	701.031439	18905.2927	17218.2275	20836.041	48577.4868	35395.4417	52036.8965	21664.2836	4.79626657	0.16690204	28.7370162	1.316E-181	1.779E-179
TS92_Nanopo	.	CDS	541859	542083	+	TS92_00518	TS92_00518	NA	small integral membrane protein	NA	168	148	228	2258	2812	2760	3976	6126	5817	116.174651	97.9331162	147.721967	3232.97114	3193.77677	3480.23924	6350.91699	6241.57962	6133.83507	3221.68317	4.74882118	0.15310683	31.01639	3.241E-211	5.355E-209
TS92_Nanopo	.	CDS	542145	542657	+	TS92_00519	TS92_00519	NA	Alkaline shock protein 23	NA	1028	863	1245	13732	16366	14517	22326	36320	32860	710.878222	571.055941	806.639687	19661.2753	18587.9625	18305.3018	35661.6129	37005.2517	34649.7886	18439.9741	4.73837805	0.14190075	33.3921982	1.779E-244	4.811E-242
TS92_Nanopo	.	CDS	2270144	2270392	+	TS92_02357	TS92_02357	NA	hypothetical protein	NA	682	725	754	7584	14976	9316	9388	14504	20556	471.613762	479.739927	488.519136	10858.6595	17009.2464	11747.0684	14995.5756	14777.6479	21675.6255	10278.1885	4.73729023	0.20864964	22.7045217	4.042E-114	2.147E-112
TS92_Nanopo	.	CDS	874327	874800	-	TS92_00881	ilvH	NA	Acetolactate synthase small subunit	NA	32	24	34	430	444	415	398	698	530	22.128505	15.8810459	22.0287143	615.667667	504.280542	523.296842	635.730624	711.169209	558.867558	401.005634	4.71715106	0.21886122	21.5531605	4.945E-103	2.132E-101
TS92_Nanopo	.	CDS	874800	876533	-	TS92_00882	ilvB	NA	acetolactate synthase large subunit	NA	88	64	90	1108	1049	1160	1002	1512	1256	60.8533886	42.3494556	58.3113027	1586.41808	1191.41957	1462.70925	1600.50775	1540.527	1324.41067	985.278496	4.67559484	0.19007657	24.5984803	1.311E-133	8.864E-132
TS92_Nanopo	.	CDS	2212806	2213168	-	TS92_02285	TS92_02285	NA	hypothetical protein	NA	26	29	22	295	437	296	253	839	496	17.9794103	19.1895971	14.253874	422.376655	496.330174	373.243049	404.120221	854.829465	523.015677	347.259791	4.55204701	0.2998909	15.1790101	4.8708E-52	7.0662E-51
TS92_Nanopo	.	CDS	993275	993790	+	TS92_01008	pfpI	NA	intracellular protease	NA	3894	3980	4020	40323	54846	46846	81093	119554	139412	2692.76245	2633.60677	2604.57152	57733.8775	62292.2762	59070.7563	129530.913	121809.633	147005.366	65041.5292	4.48530879	0.11118605	40.3405699	0	0
TS92_Nanopo	.	CDS	1301555	1301998	+	TS92_01354	TS92_01354	NA	hypothetical protein	NA	195	291	173	2358	3036	2265	2592	4033	4204	134.845577	192.557681	112.087282	3376.14967	3448.18857	2856.0659	4140.23562	4109.09086	4432.97965	2533.57787	4.42579327	0.19035708	23.2499541	1.424E-119	8.823E-118
TS92_Nanopo	.	CDS	2096849	2097064	+	TS92_02175	TS92_02175	NA	hypothetical protein	NA	695	593	924	6981	9292	8574	14959	25616	27980	480.603467	392.394175	598.662707	9995.29298	10553.5468	10811.4389	23894.2071	26099.2987	29503.9892	12481.0482	4.38648442	0.16764514	26.1652944	6.602E-151	6.136E-149
TS92_Nanopo	.	CDS	2791261	2792331	-	TS92_02909	TS92_02909	NA	NAD/NADP octopine/nopaline dehydrogenase family%0Aprotein	NA	390	556	480	4359	5713	5830	10969	15668	17793	269.691154	367.910896	310.993614	6241.152	6488.6368	7351.37492	17520.9277	15963.6091	18762.133	8141.82546	4.38175058	0.15054047	29.106795	2.945E-186	4.379E-184
TS92_Nanopo	.	CDS	994986	996719	-	TS92_01010	cidC	NA	thiamine pyrophosphate enzyme	NA	1264	1228	1530	12059	17184	15447	11302	14838	18292	874.075946	812.58018	991.292145	17265.8986	19517.0199	19477.9911	18052.833	15117.9495	19288.312	12377.5503	4.37397658	0.14211427	30.7778847	5.181E-208	8.109E-206
TS92_Nanopo	.	CDS	2270630	2270728	-	TS92_02358	TS92_02358	NA	hypothetical protein	NA	99	123	140	879	1573	1143	1864	3396	3223	68.4600622	81.3903601	90.7064708	1258.53925	1786.56147	1441.27299	2977.39167	3460.07254	3398.54743	1618.10469	4.18840575	0.18231863	22.9729993	8.681E-117	5.062E-115
TS92_Nanopo	.	CDS	1168219	1168626	+	TS92_01235	rot	NA	repressor of toxins%2C Rot	NA	895	946	1160	7853	11710	9757	10204	17572	19711	618.906623	625.977891	751.567901	11243.8097	13299.8314	12303.1501	16298.9831	17903.532	20784.6008	10425.5955	4.18645025	0.14693818	28.4912345	1.504E-178	1.945E-176
TS92_Nanopo	.	CDS	2562299	2563468	+	TS92_02665	TS92_02665	NA	hypothetical protein	NA	27	18	20	236	176	235	144	103	145	18.6709261	11.9107844	12.9580673	337.901324	199.89499	296.324718	230.01309	104.943307	152.897728	151.723882	4.15323555	0.33184897	12.5154389	6.1467E-36	5.4732E-35
TS92_Nanopo	.	CDS	878345	878434	-	TS92_00884	TS92_00884	NA	hypothetical protein	NA	156	83	196	1224	1716	1210	1148	1923	1686	107.876462	54.9219503	126.989059	1752.50517	1948.97615	1525.75706	1833.71547	1959.28136	1777.83152	1231.9838	4.11956602	0.24140472	17.064977	2.705E-65	5.5865E-64
TS92_Nanopo	.	CDS	2558903	2560183	-	TS92_02662	TS92_02662	NA	PTS system lactose/cellobiose-specific IIBC%0Acomponent	NA	476	609	520	3367	6868	4812	1812	2474	2990	329.161511	402.981539	336.909749	4820.82101	7800.44767	6067.72146	2894.33139	2520.67711	3152.8566	3147.32311	4.09530254	0.19244925	21.2799093	1.743E-100	7.404E-99
TS92_Nanopo	.	CDS	1163630	1164100	+	TS92_01229	ribH	NA	6%2C7-dimethyl-8-ribityllumazine synthase	NA	388	1685	470	9912	7098	9836	6677	8088	6910	268.308123	1114.98176	304.514581	14191.8556	8061.67408	12402.7656	10665.2597	8240.5968	7286.3676	6948.48044	4.02041718	0.57537545	6.98746737	2.7989E-12	8.8932E-12
TS92_Nanopo	.	CDS	2558566	2558880	-	TS92_02661	TS92_02661	NA	PTS system lactose/cellobiose-specific IIA%0Acomponent	NA	147	200	186	1137	2099	1454	527	906	860	101.65282	132.342049	120.510026	1627.93985	2383.97491	1833.43038	841.784018	923.093558	906.841698	985.729923	4.01486298	0.18095257	22.1873775	4.548E-109	2.147E-107
TS92_Nanopo	.	CDS	494438	494950	+	TS92_00470	TS92_00470	NA	hypothetical protein	NA	489	483	571	3390	5296	4604	5172	7938	10984	338.151216	319.606048	369.95282	4853.75207	6015.02197	5805.44256	8261.30349	8087.76674	11582.2665	5070.3626	3.99325369	0.17591268	22.7002038	4.459E-114	2.327E-112
TS92_Nanopo	.	CDS	1713371	1713589	+	TS92_01779	TS92_01779	NA	hypothetical protein	NA	488	563	635	4086	6164	3915	4891	9249	6651	337.459701	372.542868	411.418635	5850.27462	7000.86771	4936.64371	7812.4585	9423.50145	7013.26062	4795.38087	3.96043167	0.17701218	22.3737807	7.087E-111	3.399E-109
TS92_Nanopo	.	CDS	2563486	2564703	+	TS92_02666	hutI_2	NA	Imidazolonepropionase	NA	36	35	47	332	329	276	207	203	206	24.8945681	23.1598586	30.4514581	475.35271	373.667339	348.023924	330.643817	206.830014	217.220221	225.582657	3.8721499	0.25099767	15.4270352	1.0771E-53	1.6343E-52
TS92_Nanopo	.	CDS	422549	423061	+	TS92_00398	isaB	NA	immunodominant antigen B	NA	43	36	54	295	372	387	1530	3520	2614	29.7351785	23.8215688	34.9867816	422.376655	422.505319	487.990067	2443.88908	3586.41206	2756.37698	1134.23263	3.86177189	0.22013519	17.5427286	6.7602E-69	1.6214E-67
TS92_Nanopo	.	CDS	1692905	1694581	-	TS92_01763	glpD	NA	DNA-directed DNA polymerase III subunit gamma%0Aand tau	NA	2369	2085	2456	14180	22842	16949	15660	24589	22625	1638.20088	1379.66586	1591.25066	20302.7151	25943.1895	21371.9474	25013.9236	25052.9222	23857.3179	16239.0148	3.85977499	0.13566612	28.4505446	4.798E-178	5.946E-176
TS92_Nanopo	.	CDS	1749988	1750431	-	TS92_01815	TS92_01815	NA	hypothetical protein	NA	241	201	192	1362	1656	1889	4171	4375	6464	166.655303	133.003759	124.397446	1950.09154	1880.83013	2381.94635	6662.39305	4457.5434	6816.07528	2730.32625	3.83772964	0.20427584	18.7869972	9.6487E-79	2.9281E-77
TS92_Nanopo	.	CDS	2560193	2560507	-	TS92_02663	celA	NA	PTS system cellobiose-specific EIIB component	NA	118	184	138	770	1544	1131	298	372	562	81.598862	121.754685	89.4106641	1102.47466	1753.62423	1426.14151	475.999312	379.018547	592.610505	669.181442	3.82451501	0.23613724	16.1961539	5.3681E-59	9.4466E-58
TS92_Nanopo	.	CDS	873285	874292	-	TS92_00880	ilvC	NA	ketol-acid reductoisomerase	NA	196	122	174	1210	1233	1142	1656	2414	1892	135.537093	80.7286498	112.735185	1732.46018	1400.4007	1440.01203	2645.15054	2459.54509	1995.05174	1333.51347	3.76320815	0.20169843	18.657598	1.0955E-77	3.2256E-76
TS92_Nanopo	.	CDS	191911	193254	+	TS92_00185	cimH	NA	Citrate/malate transporter	NA	37	33	35	318	220	208	44	33	44	25.5860839	21.8364381	22.6766177	455.307716	249.868737	262.278899	70.2817776	33.622613	46.396552	131.983937	3.70991958	0.30742207	12.0678375	1.5618E-33	1.2796E-32
TS92_Nanopo	.	CDS	437636	438052	+	TS92_00411	lrgA_2	NA	murein hydrolase regulator	NA	178	154	207	734	1624	1598	157	337	622	123.089809	101.903378	134.115996	1050.93039	1844.48559	2015.00808	250.778161	343.3582	655.878531	724.394237	3.68996156	0.32104412	11.4936277	1.4202E-30	1.0327E-29
TS92_Nanopo	.	CDS	206369	207766	-	TS92_00202	ygcS	NA	Inner membrane metabolite transport protein%0AYgcS	NA	85	92	114	491	686	743	893	1171	2004	58.7788413	60.8773425	73.8609834	703.006568	779.136153	936.890491	1426.40062	1193.09333	2113.15205	816.132931	3.59819231	0.23436353	15.3530386	3.3798E-53	5.0765E-52
TS92_Nanopo	.	CDS	1238232	1238738	-	TS92_01301	uspA_1	NA	universal stress protein	NA	17492	13264	19642	87264	135318	103046	156080	227502	259746	12095.994	8776.92468	12726.1179	124943.31	153689.717	129936.497	249308.633	231794.294	273893.609	133018.344	3.5822344	0.16896245	21.2013634	9.277E-100	3.8859E-98
TS92_Nanopo	.	CDS	2328520	2329092	-	TS92_02429	yfiA	NA	sigma 54 modulation protein/ribosomal protein%0AS30EA	NA	14455	12704	17640	75249	118972	93923	184970	308595	284641	9995.8606	8406.36695	11429.0153	107740.41	135124.47	118432.794	295455.009	314417.281	300144.567	144571.753	3.5814437	0.14856029	24.1076779	2.077E-128	1.372E-126
TS92_Nanopo	.	CDS	2004715	2005251	-	TS92_02091	TS92_02091	NA	hypothetical protein	NA	736	777	920	4354	6084	4996	9214	17576	16830	508.955614	514.14886	596.071094	6233.99307	6910.00635	6299.73741	14717.6432	17907.6075	17746.6811	7937.20491	3.57190081	0.13404996	26.6460415	1.989E-156	2.113E-154
TS92_Nanopo	.	CDS	1162437	1163609	+	TS92_01228	ribA	NA	bifunctional%0A3%2C4-dihydroxy-2-butanone-4-phosphate synthase/GTP%0Acyclohydro	NA	1124	4890	1335	19943	14185	21804	17727	19258	17548	777.263737	3235.7631	864.95099	28554.0937	16110.8547	27493.89	28315.5698	19621.3419	18503.7885	15941.9463	3.57071644	0.58669373	6.08616776	1.1565E-09	3.1467E-09
TS92_Nanopo	.	CDS	2551175	2551762	-	TS92_02655	TS92_02655	NA	hypothetical protein	NA	672	654	677	3372	4676	4672	6992	8888	11646	464.698604	432.7585	438.630577	4827.97993	5310.84643	5891.18758	11168.4134	9055.69044	12280.3237	5541.16991	3.5671725	0.15113456	23.6026262	3.622E-123	2.292E-121
TS92_Nanopo	.	CDS	438049	438750	+	TS92_00412	lrgB_2	NA	antiholin-like protein LrgB	NA	340	364	398	1452	3122	2766	465	862	1662	235.115365	240.862529	257.865538	2078.95221	3545.86453	3487.80498	742.750604	878.263407	1752.52431	1468.88928	3.56600962	0.29594425	12.0495991	1.949E-33	1.5793E-32
TS92_Nanopo	.	CDS	2339618	2339938	-	TS92_02442	TS92_02442	NA	hypothetical protein	NA	510	701	493	2910	3970	3640	2598	3784	4303	352.673048	463.858881	319.416358	4166.49514	4508.99494	4589.88074	4149.8195	3855.39296	4537.37189	2993.76705	3.52643987	0.15944529	22.1169275	2.172E-108	1.009E-106
TS92_Nanopo	.	CDS	1158510	1160024	+	TS92_01225	TS92_01225	NA	pyridine nucleotide-disulfide oxidoreductase	NA	282	373	384	1740	2458	2155	3255	5047	6102	195.00745	246.817921	248.794891	2491.30637	2791.71526	2717.36071	5199.25423	5142.22206	6434.35819	2829.64856	3.51513511	0.15442646	22.7625178	1.079E-114	5.941E-113
TS92_Nanopo	.	CDS	1696216	1697034	-	TS92_01765	glpF	NA	MIP family major intrinsic protein channel%0Aprotein	NA	2461	2376	2882	12508	18315	16156	11996	15307	16728	1701.82033	1572.22354	1867.25749	17908.7702	20801.5724	20372.0091	19161.3683	15595.7981	17639.1255	12957.7717	3.50920783	0.13353553	26.279207	3.315E-152	3.181E-150
TS92_Nanopo	.	CDS	583792	584448	+	TS92_00564	TS92_00564	NA	(S)-2-haloacid dehalogenase	NA	29	38	27	148	243	160	409	795	652	20.0539576	25.1449893	17.4933908	211.90422	275.991378	201.752999	653.301069	809.999314	687.512543	322.572651	3.41423978	0.23267747	14.6737017	9.5017E-49	1.2559E-47
TS92_Nanopo	.	CDS	587623	588567	-	TS92_00569	czrB	NA	cation diffusion facilitator family transporter	NA	2277	2362	2566	10628	14948	14202	17089	26566	29246	1574.58143	1562.9596	1662.52003	15217.0139	16977.4449	17908.1006	27296.484	27067.2224	30838.9445	15567.2524	3.37401146	0.11642736	28.9795404	1.191E-184	1.687E-182
TS92_Nanopo	.	CDS	1749146	1749919	-	TS92_01814	TS92_01814	NA	phosphosugar-binding transcriptional regulator%0ARpiR family protein	NA	270	243	292	1182	1816	1479	5627	7207	8075	186.709261	160.795589	189.187782	1692.37019	2062.55285	1864.95429	8988.08096	7342.97491	8514.82176	3444.7164	3.37204295	0.14822048	22.7501831	1.429E-114	7.727E-113
TS92_Nanopo	.	CDS	1679315	1679524	-	TS92_01746	TS92_01746	NA	hypothetical protein	NA	566	756	601	2877	4346	3358	5408	8192	8951	391.397931	500.252945	389.389921	4119.24622	4936.04333	4234.29108	8638.26939	8346.55897	9438.53493	4554.88719	3.35914765	0.14739458	22.7901701	5.739E-115	3.282E-113
TS92_Nanopo	.	CDS	2460596	2462026	-	TS92_02569	bglA_3	NA	glycoside hydrolase	NA	266	396	427	1600	2546	1858	596	759	853	183.943197	262.037257	276.654736	2290.85643	2891.66275	2342.85671	951.998624	773.3201	899.460429	1208.0878	3.35555019	0.18464371	18.1731089	8.4285E-74	2.2381E-72
TS92_Nanopo	.	CDS	2796545	2797336	-	TS92_02916	TS92_02916	NA	hypothetical protein	NA	560	662	587	2810	3938	3012	4502	6804	6457	387.248837	438.052182	380.319274	4023.31661	4472.6504	3798.00021	7191.1037	6932.37149	6808.69401	3825.75075	3.3395675	0.12093926	27.6135925	7.642E-168	8.741E-166
TS92_Nanopo	.	CDS	2557187	2558569	-	TS92_02660	lacG_2	NA	6-phospho-beta-galactosidase	NA	1414	1806	1580	6434	10875	8453	3686	5214	6402	977.803313	1195.0487	1023.68731	9212.10643	12351.466	10658.8632	5887.69619	5312.37286	6750.69832	5929.97136	3.31538394	0.1602491	20.6889393	4.3569E-95	1.6612E-93
TS92_Nanopo	.	CDS	1519419	1521071	-	TS92_01590	qoxB_1	NA	Cytochrome aa3 quinol oxidase%2C subunit I	NA	884	836	542	2888	2832	6840	1430	1380	2705	611.299949	553.189764	351.163623	4134.99586	3216.49211	8624.94073	2284.15777	1406.03655	2852.33348	2670.5122	3.31073603	0.34445759	9.6114476	7.1536E-22	3.6681E-21
TS92_Nanopo	.	CDS	976793	977008	+	TS92_00986	TS92_00986	NA	hypothetical protein	NA	249	298	283	1401	1725	1176	1464	2823	1984	172.187429	197.189653	183.356652	2005.93116	1959.19805	1482.88455	2338.46642	2876.26172	2092.06271	1478.61537	3.27776282	0.17889027	18.3227558	5.4483E-75	1.5432E-73
TS92_Nanopo	.	CDS	2026146	2027051	+	TS92_02112	TS92_02112	NA	Pesticidal crystal protein Cry22Aa	NA	2942	4211	2778	16826	19432	14996	13194	23448	18380	2034.43942	2786.46184	1799.87554	24091.219	22070.2241	18909.2999	21074.9494	23890.3949	19381.1051	15115.3299	3.27291411	0.1850203	17.6894869	5.0533E-70	1.2524E-68
TS92_Nanopo	.	CDS	1670143	1671207	-	TS92_01736	thrC	NA	threonine synthase	NA	1394	856	1464	5927	6659	6040	2061	2686	2984	963.972997	566.423969	948.530523	8486.1913	7563.07237	7616.17573	3292.06235	2736.67693	3146.5298	3924.404	3.22763733	0.19902718	16.2170678	3.82E-59	6.7623E-58
TS92_Nanopo	.	CDS	2460260	2460577	-	TS92_02568	TS92_02568	NA	hypothetical protein	NA	30	41	52	155	272	187	64	87	101	20.7454734	27.13012	33.6909749	221.926717	308.928621	235.798818	102.22804	88.6414344	106.501176	127.287931	3.19213591	0.23859344	13.3789759	8.0245E-41	8.6155E-40
TS92_Nanopo	.	CDS	1160742	1161788	+	TS92_01226	ribD	NA	bifunctional pyrimidine reductase / riboflavin%0Abiosynthesis protein	NA	566	2238	638	6601	5028	7388	7446	9745	7228	391.397931	1480.90753	413.362346	9451.21457	5710.63641	9315.94475	11893.5935	9928.85952	7621.68813	6245.28941	3.17069517	0.55901413	5.67194106	1.4119E-08	3.5675E-08
TS92_Nanopo	.	CDS	871723	873255	-	TS92_00879	leuA	NA	2-isopropylmalate synthase	NA	267	180	282	1119	1184	1067	1920	2898	2320	184.634713	119.107844	182.708748	1602.16772	1344.74811	1345.44032	3066.8412	2952.67674	2446.36365	1471.63212	3.11640628	0.1897505	16.4237051	1.2942E-60	2.4515E-59
TS92_Nanopo	.	CDS	1161793	1162425	+	TS92_01227	ribB	NA	riboflavin synthase subunit alpha	NA	606	2759	721	7497	5561	9116	8798	10088	9072	419.058563	1825.65856	467.138325	10734.0942	6316.00022	11494.8771	14053.1609	10278.3309	9566.12545	7239.38269	3.10873202	0.59645664	5.21200005	1.8682E-07	4.4025E-07
TS92_Nanopo	.	CDS	2779918	2784411	-	TS92_02901	gltB	NA	glutamate synthase (NADPH)%2C large subunit	NA	1098	946	1084	4766	4944	4379	1834	2175	2250	759.284326	625.977891	702.327245	6823.8886	5615.23199	5521.7274	2929.47227	2216.03586	2372.55095	3062.94406	3.08832312	0.15877121	19.4514055	2.8355E-84	9.0676E-83
TS92_Nanopo	.	CDS	1694696	1696189	-	TS92_01764	glpK	NA	glycerol kinase	NA	16305	15074	19056	62770	94198	70794	73620	102047	100762	11275.1648	9974.62023	12346.4465	89873.1615	106986.979	89268.1365	117594.192	103972.327	106250.213	71949.0267	3.07835333	0.13516003	22.7756185	8E-115	4.489E-113
TS92_Nanopo	.	CDS	464455	465036	+	TS92_00435	TS92_00435	NA	IS256 family transposase ISBsu2	NA	82	77	99	320	397	445	792	1029	1111	56.7042939	50.9516888	64.1424329	458.171287	450.899494	561.12553	1265.072	1048.41421	1171.51294	569.665985	3.07361445	0.17700194	17.3648627	1.5226E-67	3.4567E-66
TS92_Nanopo	.	CDS	2360304	2361818	-	TS92_02463	TS92_02463	NA	ABC-type proline/glycine betaine transport%0Asystems%2C permease component	NA	1392	1564	1614	5343	8165	7126	8540	11642	16210	962.589966	1034.91482	1045.71603	7650.0287	9273.53745	8985.57421	13641.0541	11861.6503	17092.9115	7949.77524	3.07283269	0.16054018	19.1405836	1.1596E-81	3.6303E-80
TS92_Nanopo	.	CDS	2540375	2541700	-	TS92_02646	malH	NA	Maltose-6'-phosphate glucosidase MalH	NA	4431	7186	5026	19715	38856	18027	5043	8627	5040	3064.10642	4755.04982	3256.3623	28227.6466	44131.3621	22731.2583	8055.2501	8789.76614	5314.51414	14258.3684	3.05354871	0.2704966	11.2886768	1.4927E-29	1.0421E-28
TS92_Nanopo	.	CDS	2616427	2617938	+	TS92_02726	mtlA_1	NA	PTS system mannitol-specific IIBC component	NA	887	1169	1144	3618	5697	4509	2615	2923	4261	613.374497	773.539276	741.201447	5180.19911	6470.46453	5685.65172	4176.97383	2978.14842	4493.08428	3456.95968	3.0044517	0.18352136	16.3711283	3.0746E-60	5.7149E-59
TS92_Nanopo	.	CDS	360769	361527	+	TS92_00343	oxaI_1	NA	OxaI/YidC membrane insertion protein	NA	1958	1843	2276	7142	9477	9022	8880	13299	13798	1353.9879	1219.53198	1474.62805	10225.8104	10763.6637	11376.3473	14184.1406	13549.9131	14549.5369	8744.17332	2.9906292	0.11543392	25.9077153	5.452E-148	4.769E-146
TS92_Nanopo	.	CDS	2544249	2545205	+	TS92_02649	TS92_02649	NA	D-isomer specific 2-hydroxyacid dehydrogenase%0Afamily protein	NA	1196	1154	1470	4207	5850	6015	5469	6466	11331	827.052873	763.613622	952.417944	6023.52064	6644.23688	7584.65182	8735.70549	6587.99442	11948.1666	5563.04003	2.96494958	0.21087962	14.0599149	6.6967E-45	8.0959E-44
TS92_Nanopo	.	CDS	2361815	2362777	-	TS92_02464	TS92_02464	NA	glycine/betaine/carnitine/choline ABC%0Asuperfamily ATP binding cassette transporter%2	NA	1463	1562	1516	5186	7082	6617	5966	7666	10976	1011.68759	1033.5914	982.221498	7425.23842	8043.50181	8343.74748	9529.57011	7810.6349	11573.8308	6194.89155	2.96003929	0.1565656	18.9060647	1.0167E-79	3.1495E-78
TS92_Nanopo	.	CDS	193282	194463	+	TS92_00186	maeB_1	NA	malate dehydrogenase%0A(oxaloacetate-decarboxylating) (NADP(+))	NA	70	82	82	410	312	252	84	65	118	48.4061046	54.26024	53.1280758	587.031961	354.3593	317.760974	134.174303	66.226359	124.427117	193.30827	2.95866392	0.2904724	10.1856971	2.2971E-24	1.3062E-23
TS92_Nanopo	.	CDS	517731	517946	-	TS92_00492	TS92_00492	NA	hypothetical protein	NA	0	0	0	2	6	1	2	6	0	0	0	0	2.86357054	6.81460192	1.26095625	3.19462625	6.11320237	0	2.24966193	2.93262746	1.04141095	2.81601367	0.00486236	0.00762693
TS92_Nanopo	.	CDS	406373	407269	-	TS92_00382	TS92_00382	NA	hypothetical protein	NA	355	442	425	1286	2035	1696	1585	2102	3306	245.488102	292.475928	275.358929	1841.27586	2311.28582	2138.58179	2531.74131	2141.65856	3486.0682	1695.99272	2.9272755	0.19518541	14.9974096	7.6341E-51	1.0811E-49
TS92_Nanopo	.	CDS	2094002	2094241	-	TS92_02169	TS92_02169	NA	hypothetical protein	NA	153	94	176	452	674	616	668	1197	1464	105.801914	62.200763	114.030992	647.166943	765.50695	776.749048	1067.00517	1219.58387	1543.73982	700.198386	2.92311293	0.22806421	12.8170609	1.3159E-37	1.2544E-36
TS92_Nanopo	.	CDS	1676245	1677045	+	TS92_01742	TS92_01742	NA	hypothetical protein	NA	580	698	797	2068	3440	2848	3886	6954	8015	401.079152	461.873751	516.37898	2960.93194	3907.03844	3591.20339	6207.15881	7085.20155	8451.55373	3731.37997	2.90380789	0.17340757	16.7455656	6.1008E-63	1.2096E-61
TS92_Nanopo	.	CDS	1999233	2000579	-	TS92_02085	TS92_02085	NA	cytochrome ubiquinol oxidase	NA	1141	1154	1102	3662	4369	5530	7985	9002	13036	789.019505	763.613622	713.989506	5243.19766	4962.16597	6973.08804	12754.5453	9171.84129	13746.033	6124.16599	2.89947894	0.18973294	15.2818953	1.0096E-52	1.4864E-51
TS92_Nanopo	.	CDS	2778445	2779902	-	TS92_02900	gltD	NA	glutamate synthase (NADPH) small subunit	NA	254	262	302	966	1233	1007	584	536	730	175.645008	173.368084	195.666816	1383.10457	1400.4007	1269.78294	932.830866	546.112745	769.760976	760.741411	2.87235867	0.19311624	14.8737295	4.8816E-50	6.7526E-49
TS92_Nanopo	.	CDS	1998217	1999233	-	TS92_02084	cydB	NA	Cytochrome bd-type quinol oxidase subunit%0A2-like protein	NA	746	755	671	2124	2232	4239	5628	5913	8707	515.870772	499.591235	434.743157	3041.11192	2535.03192	5345.19353	8989.67828	6024.56094	9181.24496	4063.00297	2.86936635	0.26257195	10.9279243	8.4765E-28	5.5283E-27
TS92_Nanopo	.	CDS	2240114	2241514	-	TS92_02319	proP_1	NA	MFS family major facilitator transporter%2C%0Aproline/betaine:cation symporter	NA	102	107	109	400	413	413	369	490	638	70.5346095	70.8029962	70.6214666	572.714108	469.071766	520.77493	589.408544	499.24486	672.750004	392.880365	2.8605713	0.17613233	16.2410344	2.5853E-59	4.604E-58
TS92_Nanopo	.	CDS	408070	408645	-	TS92_00384	TS92_00384	NA	hypothetical protein	NA	170	198	104	585	737	518	180	145	162	117.557683	131.018628	67.3819497	837.594384	837.06027	653.175336	287.516363	147.735724	170.823669	361.096001	2.83390803	0.27947993	10.1399341	3.6735E-24	2.0459E-23
TS92_Nanopo	.	CDS	21705	22220	+	TS92_00019	yceI	NA	base-induced perplasmic lipid-binding protein	NA	331	355	371	1284	1224	1451	2920	4333	5032	228.891723	234.907137	240.372148	1838.41229	1390.17879	1829.64751	4664.15433	4414.75098	5306.0784	2238.59926	2.8288241	0.15482351	18.2712827	1.4012E-74	3.9312E-73
TS92_Nanopo	.	CDS	2745514	2745846	-	TS92_02864	spoVG	NA	putative septation protein spoVG	NA	5356	5316	5833	18922	22458	20593	36087	52264	66352	3703.75852	3517.65166	3779.22032	27092.2409	25507.055	25966.872	57642.2388	53250.0681	69966.0004	30047.234	2.82612001	0.13010255	21.722249	1.264E-104	5.612E-103
TS92_Nanopo	.	CDS	2403307	2403600	-	TS92_02506	TS92_02506	NA	hypothetical protein	NA	1422	1394	1680	4456	6613	5958	4929	7171	9709	983.335439	922.424081	1088.47765	6380.03517	7510.82709	7512.77732	7873.1564	7306.2957	10237.821	5535.01665	2.82190144	0.16139771	17.4841482	1.8922E-68	4.4662E-67
TS92_Nanopo	.	CDS	2617963	2620005	+	TS92_02727	bglG	NA	BglG family transcriptional antiterminator	NA	3200	3319	3531	9969	16634	11575	8561	10635	13227	2212.8505	2196.2163	2287.74677	14273.4674	18892.3481	14595.5686	13674.5977	10835.6512	13947.4362	10323.987	2.81953068	0.15710046	17.9473102	5.0372E-72	1.3123E-70
TS92_Nanopo	.	CDS	1236328	1236456	+	TS92_01298	TS92_01298	NA	hypothetical protein	NA	22	25	27	92	97	95	109	337	312	15.2133472	16.5427561	17.4933908	131.724245	110.169398	119.790843	174.107131	343.3582	328.993732	139.710338	2.80995891	0.29979197	9.37302931	7.048E-21	3.4083E-20
TS92_Nanopo	.	CDS	1521068	1521547	-	TS92_01591	cbaB	NA	Cytochrome c oxidase subunit 2	NA	266	206	156	526	580	1319	258	270	538	183.943197	136.31231	101.072925	753.119053	658.744853	1663.20129	412.106787	275.094107	567.303295	527.877535	2.79196359	0.35012285	7.97423992	1.5332E-15	5.75E-15
TS92_Nanopo	.	CDS	75522	75878	-	TS92_00069	TS92_00069	NA	MerR family transcriptional regulator	NA	192	210	188	579	940	678	1082	2176	2687	132.77103	138.959151	121.805832	829.003672	1067.62097	854.928335	1728.2928	2217.05473	2833.35307	1102.64329	2.78091987	0.20289917	13.7059205	9.3573E-43	1.0541E-41
TS92_Nanopo	.	CDS	1521560	1521715	-	TS92_01592	TS92_01592	NA	hypothetical protein	NA	132	145	94	300	306	780	129	141	289	91.2800829	95.9479855	60.9029161	429.535581	347.544698	983.545872	206.053393	143.660256	304.740989	295.912419	2.74584806	0.36448493	7.53350235	4.9397E-14	1.7385E-13
TS92_Nanopo	.	CDS	2745903	2746280	-	TS92_02865	TS92_02865	NA	endoribonuclease L-PSP	NA	2235	2204	2486	7205	8462	8842	14117	22895	23563	1545.53777	1458.40938	1610.68776	10316.0129	9610.86025	11149.3751	22549.2694	23326.9614	24846.4081	11823.7247	2.74425851	0.11075643	24.7774194	1.57E-135	1.112E-133
TS92_Nanopo	.	CDS	1378572	1379276	+	TS92_01433	TS92_01433	NA	hypothetical protein	NA	2153	2331	2815	6996	11004	8028	9729	17746	13058	1488.83347	1542.44658	1823.84797	10016.7698	12497.9799	10122.9567	15540.2594	18080.8149	13769.2313	9431.46	2.73415995	0.15915965	17.1787256	3.8324E-66	8.0833E-65
TS92_Nanopo	.	CDS	1690119	1690592	+	TS92_01759	bsaA	NA	putative glutathione peroxidase	NA	1634	1532	1703	4729	6573	5860	10010	15490	15650	1129.93678	1013.74009	1103.37943	6770.91255	7465.39641	7389.2036	15989.1044	15782.2508	16502.41	8127.37045	2.72949893	0.10291846	26.5209847	5.552E-155	5.694E-153
TS92_Nanopo	.	CDS	2354787	2356301	+	TS92_02459	TS92_02459	NA	POT family proton (H+)-dependent oligopeptide%0Atransporter	NA	6260	6276	6957	20830	22735	25622	8055	5146	9366	4328.88878	4152.89349	4507.4637	29824.0872	25821.6625	32308.2209	12866.3572	5243.0899	9876.13878	14325.4225	2.72050923	0.25725072	10.5753221	3.8784E-26	2.3782E-25
TS92_Nanopo	.	CDS	75979	76299	-	TS92_00070	TS92_00070	NA	hypothetical protein	NA	278	372	388	917	1614	1140	1730	4619	2782	192.241387	246.156211	251.386505	1312.94709	1833.12792	1437.49012	2763.35171	4706.14696	2933.52745	1741.81948	2.70101953	0.22957301	11.7654056	5.8843E-32	4.5454E-31

TS92_Nanopo	.	CDS	2457382	2457885	-	TS92_02565	TS92_02565	NA	hypothetical protein	NA	236	314	340	690	1570	824	402	648	592	163.197724	207.777017	220.287143	987.931837	1783.15417	1039.02795	642.119877	660.225856	624.244518	703.107343	2.65810331	0.22995344	11.5593107	6.6238E-31	4.8882E-30
TS92_Nanopo	.	CDS	2267489	2268940	-	TS92_02354	egtB	NA	Hercynine oxygenase	NA	359	306	424	1024	1478	1164	774	1188	1306	248.254165	202.483335	274.711026	1466.14812	1678.66361	1467.75307	1236.32036	1210.41407	1377.13402	1017.98686	2.65584073	0.15086982	17.6035259	2.3146E-69	5.5963E-68
TS92_Nanopo	.	CDS	5264	5995	+	TS92_00006	TS92_00006	NA	hypothetical protein	NA	2368	2538	2536	7215	9122	7868	5922	8050	8116	1637.50937	1679.4206	1643.08293	10330.3307	10360.4665	9921.20375	9459.28834	8201.87985	8558.05491	6865.69299	2.61993667	0.1015281	25.8050396	7.784E-147	6.614E-145
TS92_Nanopo	.	CDS	199110	199277	-	TS92_00195	TS92_00195	NA	hypothetical protein	NA	211	227	301	689	862	842	2647	4285	5235	145.90983	150.208225	195.018912	986.500052	979.031143	1061.72516	4228.08785	4365.84536	5520.13522	1959.16242	2.60617868	0.1689522	15.4255383	1.1023E-53	1.6641E-52
TS92_Nanopo	.	CDS	360470	360739	+	TS92_00342	TS92_00342	NA	hypothetical protein	NA	1868	1858	1978	5010	6358	7000	6530	8734	9879	1291.75148	1229.45763	1281.55285	7173.24421	7221.20651	8826.69373	10430.4547	8898.78492	10417.0804	6307.80294	2.60057694	0.13270915	19.5960638	1.6706E-85	5.5823E-84
TS92_Nanopo	.	CDS	2236731	2237144	-	TS92_02314	uspA_2	NA	universal stress protein	NA	923	1045	962	2578	3775	3026	6214	10526	9544	638.269065	691.487205	623.283035	3691.14243	4287.52038	3815.6536	9925.70377	10724.5947	10063.8339	4940.16534	2.58719332	0.11474116	22.5480841	1.402E-112	6.951E-111
TS92_Nanopo	.	CDS	6011	6424	+	TS92_00007	bdbC	NA	Disulfide bond formation protein C	NA	878	841	793	2459	2780	2740	1936	2586	2762	607.150855	556.498316	513.787367	3520.75998	3157.43223	3455.02012	3092.39821	2634.79022	2912.43811	2272.25282	2.58612276	0.12543939	20.6165117	1.9513E-94	7.254E-93
TS92_Nanopo	.	CDS	720805	721590	-	TS92_00702	fabG_4	NA	3-oxoacyl-ACP reductase	NA	2640	3046	3252	8338	10378	9484	10613	15765	15592	1825.60166	2015.5694	2106.98174	11938.2256	11786.9898	11958.909	16952.2842	16062.4392	16441.2509	10120.9168	2.5791604	0.10001972	25.7865183	1.256E-146	1.01E-144
TS92_Nanopo	.	CDS	2456005	2457360	-	TS92_02564	TS92_02564	NA	PTS system lactose/cellobiose-specific IIBC%0Acomponent	NA	676	754	852	1736	3349	2201	1704	2517	2381	467.464667	498.929524	552.013665	2485.57923	3803.68364	2775.3647	2721.82157	2564.48839	2510.68614	2042.22573	2.56148869	0.17337169	14.7745497	2.1378E-49	2.8769E-48
TS92_Nanopo	.	CDS	2115864	2117426	-	TS92_02193	alsT	NA	sodium/alanine symporter	NA	1612	1384	1892	4318	6306	4710	2013	2765	2702	1114.72344	915.806978	1225.83316	6182.4488	7162.14662	5939.10392	3215.39132	2817.16743	2849.17008	3491.31019	2.55402094	0.14932767	17.1034671	1.3983E-65	2.9286E-64
TS92_Nanopo	.	CDS	1669235	1670146	-	TS92_01735	thrB	NA	homoserine kinase%2C ThrB	NA	1293	828	1292	3125	3432	3926	1873	2240	2663	894.129903	547.896082	837.091145	4474.32897	3897.9523	4950.51422	2991.76749	2282.26222	2808.04586	2631.55424	2.52545172	0.20115186	12.5549507	3.7341E-36	3.3755E-35
TS92_Nanopo	.	CDS	2132176	2132571	-	TS92_02208	spxA	NA	transcriptional regulator Spx	NA	16658	15546	23870	49922	65853	55346	47638	89856	77665	11519.2699	10286.9475	15465.4533	71477.5843	74793.6634	69788.8844	76092.8027	91551.3187	81895.1866	55874.5679	2.52040948	0.16629298	15.1564394	6.8694E-52	9.9172E-51
TS92_Nanopo	.	CDS	2797333	2797893	-	TS92_02917	TS92_02917	NA	hypothetical protein	NA	312	356	342	882	1090	1087	1346	1694	2104	215.752923	235.568847	221.58295	1262.83461	1237.98602	1370.65944	2149.98347	1725.9608	2218.59876	1182.10309	2.51281218	0.14392544	17.459125	2.934E-68	6.8706E-67
TS92_Nanopo	.	CDS	2620446	2621564	+	TS92_02729	mtlD	NA	putative mannitol-1-phosphate 5-dehydrogenase	NA	1575	1710	1833	4438	6394	4690	4060	5766	5668	1089.13735	1131.52452	1187.60686	6354.26303	7262.09412	5913.8848	6485.09129	5874.78748	5976.71947	4586.12321	2.51074808	0.12162331	20.6436419	1.1135E-94	4.1918E-93
TS92_Nanopo	.	CDS	2593546	2594370	+	TS92_02699	TS92_02699	NA	hypothetical protein	NA	88	103	95	252	338	270	570	743	851	60.8533886	68.1561552	61.5508195	360.809888	383.889242	340.458187	910.468482	757.018227	897.351495	426.728432	2.49383193	0.16498729	15.1152976	1.2839E-51	1.8446E-50
TS92_Nanopo	.	CDS	982574	982984	+	TS92_00993	TS92_00993	NA	hypothetical protein	NA	406	627	444	1504	1509	1369	2033	4440	3620	280.755407	414.892323	287.669093	2153.40505	1713.87238	1726.2491	3247.33759	4523.76975	3817.17087	2018.34684	2.486191	0.19867385	12.5139319	6.2645E-36	5.5614E-35
TS92_Nanopo	.	CDS	2093804	2093998	-	TS92_02168	TS92_02168	NA	hypothetical protein	NA	79	84	94	220	282	268	323	547	748	54.6297466	55.5836605	60.9029161	314.99276	320.28629	337.936274	515.93214	557.320283	788.741384	334.036162	2.48348675	0.20439582	12.1503795	5.71E-34	4.7434E-33
TS92_Nanopo	.	CDS	2110773	2111906	+	TS92_02188	ybhS	NA	putative multidrug ABC transporter permease%0AYbhS	NA	136	166	160	382	586	417	742	1358	1550	94.0461461	109.843901	103.664538	546.941974	665.559455	525.818755	1185.20634	1383.62147	1634.42399	694.347397	2.48126769	0.17941045	13.8301179	1.6775E-43	1.9411E-42
TS92_Nanopo	.	CDS	893975	895402	+	TS92_00901	TS92_00901	NA	Polyphosphate:AMP phosphotransferase	NA	1580	947	1816	3434	5309	4153	3615	6310	6397	1092.59493	626.639602	1176.59251	4916.75062	6029.78694	5236.75129	5774.28695	6429.05116	6745.42598	4225.32	2.45770657	0.21641605	11.356397	6.8929E-30	4.8692E-29
TS92_Nanopo	.	CDS	373058	374089	-	TS92_00356	TS92_00356	NA	zinc-binding alcohol dehydrogenase	NA	676	728	602	1460	2636	1804	1138	1586	1410	467.464667	481.725058	390.037824	2090.4065	2993.88178	2274.76507	1817.74234	1615.92316	1486.7986	1513.19389	2.44294408	0.1738669	14.0506565	7.6323E-45	9.1897E-44
TS92_Nanopo	.	CDS	892161	893861	-	TS92_00900	TS92_00900	NA	hypothetical protein	NA	925	714	994	2192	3106	2338	1332	2141	2113	639.652096	472.461115	644.015943	3138.47331	3527.69226	2948.1157	2127.62108	2181.39438	2228.08896	1989.72387	2.44094755	0.1520788	16.0505446	5.6665E-58	9.6298E-57
TS92_Nanopo	.	CDS	870686	871726	-	TS92_00878	leuB_1	NA	3-isopropylmalate dehydrogenase	NA	68	82	88	232	232	220	408	730	463	47.023073	54.26024	57.0154959	332.174183	263.497941	277.410374	651.703756	743.772955	488.218263	323.897365	2.43486649	0.21235484	11.4660275	1.9543E-30	1.4141E-29
TS92_Nanopo	.	CDS	660634	662034	+	TS92_00642	nhaC	NA	putative Na+/H+ antiporter	NA	606	551	683	1552	1813	1918	3439	3926	6291	419.058563	364.602345	442.517997	2222.13074	2059.14555	2418.51408	5493.15984	4000.07208	6633.65247	2672.5393	2.43058673	0.19096851	12.7276833	4.1497E-37	3.8931E-36
TS92_Nanopo	.	CDS	1181985	1182410	+	TS92_01247	TS92_01247	NA	hypothetical protein	NA	4223	4498	4928	12347	14227	11950	13094	21824	26096	2920.27114	2976.37268	3192.86777	17678.2527	16158.5569	15068.4271	20915.2181	22235.7548	27517.3732	14295.8994	2.4175344	0.14027735	17.2339606	1.4768E-66	3.2295E-65
TS92_Nanopo	.	CDS	1923214	1923378	-	TS92_01996	TS92_01996	NA	hypothetical protein	NA	0	4	0	6	8	3	6	10	3	0	2.64684098	0	8.59071163	9.0861359	3.78286874	9.58387876	10.1886706	3.16340127	5.22694532	2.41560107	0.81201227	2.97483323	0.00293148	0.00469985
TS92_Nanopo	.	CDS	2620012	2620446	+	TS92_02728	mtlA_2	NA	phosphoenolpyruvate-dependent sugar%0Aphosphotransferase system%2C EIIA 2%2C Mt	NA	945	968	1096	2406	3332	2750	2156	2959	3161	653.482412	640.535517	710.102086	3444.87536	3784.3756	3467.62968	3443.8071	3014.82764	3333.17047	2499.20065	2.40946747	0.11465035	21.0157891	4.7036E-98	1.9162E-96
TS92_Nanopo	.	CDS	2774145	2774891	+	TS92_02895	TS92_02895	NA	hypothetical protein	NA	659	790	901	1761	2488	2292	3074	4955	4960	455.708899	522.751093	583.76093	2521.37386	2825.78826	2890.11172	4910.14055	5048.48629	5230.15677	2776.47538	2.38955983	0.13110693	18.2260377	3.2073E-74	8.9146E-73
TS92_Nanopo	.	CDS	372858	373016	-	TS92_00355	TS92_00355	NA	hypothetical protein	NA	118	141	124	253	490	342	224	298	284	81.598862	93.3011445	80.340017	362.241674	556.525824	431.247036	357.79814	303.622384	299.468654	285.127082	2.38544597	0.19679985	12.1211777	8.1578E-34	6.7206E-33
TS92_Nanopo	.	CDS	1680305	1680778	-	TS92_01749	TS92_01749	NA	hypothetical protein	NA	67	79	72	178	210	211	492	678	602	46.3315573	52.2751093	46.6490421	254.857778	238.511067	266.061768	785.878058	690.791868	634.789189	335.127271	2.36693771	0.17775663	13.3156084	1.8784E-40	1.9809E-39
TS92_Nanopo	.	CDS	371794	372831	-	TS92_00354	TS92_00354	NA	oxidoreductase%2C zinc-binding dehydrogenase%0Afamily	NA	763	850	724	1725	2780	1919	1389	1874	1913	527.62654	562.453708	469.082035	2469.82959	3157.43223	2419.77504	2218.66793	1909.35687	2017.19555	1750.15772	2.35652893	0.15207029	15.4963139	3.6737E-54	5.6318E-53
TS92_Nanopo	.	CDS	374110	375369	-	TS92_00357	TS92_00357	NA	PTS system galactitol-specific IIC component	NA	958	1075	793	2162	3184	2395	1194	1581	1427	662.472117	711.338513	513.787367	3095.51976	3616.28209	3019.99021	1907.19187	1610.82882	1504.72454	1849.12614	2.35260612	0.16632617	14.1445339	2.0185E-45	2.4602E-44
TS92_Nanopo	.	CDS	1200354	1200905	+	TS92_01265	TS92_01265	NA	general stress protein	NA	8516	8771	9290	21097	27422	22956	23547	33070	33689	5888.94838	5803.86055	6019.02224	30206.3739	31145.0023	28946.5116	37611.9322	33693.9337	35523.9418	23871.0585	2.34547054	0.09534539	24.5997255	1.272E-133	8.796E-132
TS92_Nanopo	.	CDS	2600311	2600643	-	TS92_02710	yflT	NA	General stress protein 17M	NA	17	29	19	61	75	44	40	72	67	11.7557683	19.1895971	12.3101639	87.3389015	85.1825241	55.4820748	63.8925251	73.3584284	70.6492951	53.2399198	2.34516171	0.30352378	7.72645137	1.1059E-14	3.9913E-14
TS92_Nanopo	.	CDS	2348700	2349116	-	TS92_02453	TS92_02453	NA	hypothetical protein	NA	2357	3106	2330	6132	7227	7570	2816	3972	4830	1629.90269	2055.27202	1509.61484	8779.70728	8208.18802	9545.43879	4498.03377	4046.93997	5093.07605	5040.68594	2.34028079	0.15762555	14.8470907	7.2654E-50	9.9573E-49
TS92_Nanopo	.	CDS	1001279	1002253	-	TS92_01016	yfhP	NA	putative protein YfhP	NA	1124	1062	1305	2826	2991	3508	3401	4168	5354	777.263737	702.73628	845.513889	4046.22518	3397.07906	4423.43451	5432.46194	4246.63791	5645.61681	3279.66326	2.33771592	0.16359004	14.2900872	2.5229E-46	3.1394E-45
TS92_Nanopo	.	CDS	528339	529088	-	TS92_00506	TS92_00506	NA	hypothetical protein	NA	343	421	185	868	1040	680	707	1199	937	237.189912	278.580013	119.862122	1242.78962	1181.19767	857.450248	1129.30038	1221.62161	988.035664	806.225248	2.33214761	0.26616504	8.76203591	1.9176E-18	8.3742E-18
TS92_Nanopo	.	CDS	575218	575520	+	TS92_00555	csoR	NA	Copper-sensing transcriptional repressor CsoR	NA	178	218	230	476	748	478	1736	2353	1454	123.089809	144.252833	149.017773	681.529789	849.553707	602.737086	2772.93559	2397.3942	1533.19515	1028.18955	2.33023987	0.22988453	10.1365668	3.8023E-24	2.1136E-23
TS92_Nanopo	.	CDS	2541722	2543299	-	TS92_02647	TS92_02647	NA	PTS system glucose-specific IIBC component	NA	2287	3538	2468	5974	11219	5362	1065	1724	1178	1581.49659	2341.13084	1599.0255	8553.48521	12742.1698	6761.24739	1701.13848	1756.52681	1242.16223	4253.15366	2.31440361	0.24903315	9.29355621	1.4922E-20	7.089E-20
TS92_Nanopo	.	CDS	1377561	1378556	+	TS92_01432	TS92_01432	NA	SigmaW regulon antibacterial protein	NA	5480	5002	6299	12618	15191	15572	16182	20138	22444	3789.50647	3309.87464	4081.14328	18066.2666	17253.4363	19635.6107	25847.721	20517.9449	23666.4594	15129.7737	2.2878825	0.1387905	16.484432	4.7474E-61	9.2279E-60
TS92_Nanopo	.	CDS	2396164	2397000	+	TS92_02497	TS92_02497	NA	hypothetical protein	NA	3334	3549	3632	7507	11470	8418	5636	8934	11450	2305.51361	2348.40966	2353.18501	10748.412	13027.2473	10614.7297	9002.45678	9102.55833	12073.6482	7952.90674	2.28356693	0.15450973	14.7794379	1.9882E-49	2.6999E-48
TS92_Nanopo	.	CDS	552320	552472	-	TS92_00530	TS92_00530	NA	hypothetical protein	NA	680	835	965	1775	3274	1464	2754	6656	4877	470.23073	552.528054	625.226745	2541.41886	3718.50112	1846.03994	4399.00035	6781.57916	5142.636	2897.46233	2.26725293	0.25066471	9.04496266	1.4972E-19	6.8396E-19
TS92_Nanopo	.	CDS	2524797	2526155	-	TS92_02632	ulaA	NA	PTS system ascorbate-specific IIC component	NA	457	450	506	732	2938	570	544	1383	609	316.022711	297.76961	327.839102	1048.06682	3336.88341	718.745061	868.938341	1409.09315	642.170458	996.169851	2.26379138	0.56042406	4.03942573	5.3582E-05	0.00010354
TS92_Nanopo	.	CDS	588587	588886	-	TS92_00570	TS92_00570	NA	transcriptional repressor%2C arsR family	NA	1129	1309	1412	2884	3371	3466	3675	6510	6562	780.721315	866.17871	914.839548	4129.26872	3828.67051	4370.47435	5870.12574	6632.82457	6919.41305	3812.50184	2.25885732	0.12586441	17.9467512	5.0881E-72	1.3123E-70
TS92_Nanopo	.	CDS	375454	375732	-	TS92_00358	TS92_00358	NA	phosphotransferase system galactitol-specific%0Aenzyme II%2C B component	NA	200	195	180	362	689	436	136	197	180	138.303156	129.033498	116.622605	518.306268	782.543454	549.776924	217.234585	200.716811	189.804076	315.815709	2.25367437	0.19234166	11.7170373	1.0425E-31	7.9705E-31
TS92_Nanopo	.	CDS	1045265	1046791	+	TS92_01080	gppA	NA	Guanosine-5'-triphosphate%2C3'-diphosphate%0Apyrophosphatase	NA	2011	2130	2046	3995	6504	5168	4843	6527	6239	1390.63823	1409.44282	1325.61028	5719.98216	7387.02849	6516.62188	7735.78747	6650.14531	6578.82018	4968.23076	2.24126912	0.13611775	16.4656642	6.4748E-61	1.2423E-59
TS92_Nanopo	.	CDS	2397474	2397725	-	TS92_02499	TS92_02499	NA	hypothetical protein	NA	572	643	681	1249	2066	1485	1918	3412	3625	395.547026	425.479687	441.22219	1788.2998	2346.4946	1872.52003	3063.64658	3476.37441	3822.44321	1959.11417	2.23970815	0.15365	14.5766883	3.9523E-48	5.1328E-47
TS92_Nanopo	.	CDS	2238268	2238450	-	TS92_02316	TS92_02316	NA	putative CsbD family general stress response%0Aprotein	NA	90	124	119	228	428	192	290	696	465	62.2364202	82.0520703	77.1005002	326.447042	486.108271	242.103599	463.220807	709.131475	490.327197	326.525265	2.21738932	0.26830312	8.26449315	1.4029E-16	5.5188E-16
TS92_Nanopo	.	CDS	1881431	1882489	+	TS92_01940	ilvE_1	NA	branched-chain amino acid aminotransferase	NA	23	34	18	72	77	41	15	21	23	15.9048629	22.4981483	11.6622605	103.08854	87.454058	51.6992061	23.9596969	21.3962083	24.2527431	40.2128582	2.21664962	0.34212006	6.47915701	9.2236E-11	2.6946E-10
TS92_Nanopo	.	CDS	2145045	2146115	-	TS92_02218	opp3C	NA	oligopeptide ABC superfamily ATP binding%0Acassette transporter%2C membrane protei	NA	12060	7134	14132	23270	33283	26184	10402	12074	16004	8339.68031	4720.64088	9156.17032	33317.6433	37801.7326	33016.8784	16615.2511	12301.8009	16875.6913	19127.2766	2.20336	0.23156828	9.51494749	1.818E-21	9.0719E-21
TS92_Nanopo	.	CDS	597867	599801	+	TS92_00581	vraE_2	NA	ABC transporter permease protein%2C involved in%0Avancomycin resistance	NA	3293	2832	2998	7255	7905	7007	3957	5110	5240	2277.16146	1873.96341	1942.41428	10387.6021	8978.23804	8835.52042	6320.56804	5206.41069	5525.40756	5705.254	2.2013192	0.13938012	15.7936388	3.4414E-56	5.6235E-55
TS92_Nanopo	.	CDS	1606475	1607200	-	TS92_01678	TS92_01678	NA	hypothetical protein	NA	390	529	478	895	1711	891	2636	6040	4138	269.691154	350.044719	309.697807	1281.44782	1943.29732	1123.51202	4210.5174	6153.95705	4363.38482	2222.8389	2.20055903	0.23118139	9.51875515	1.7527E-21	8.7603E-21
TS92_Nanopo	.	CDS	983000	983710	+	TS92_00994	TS92_00994	NA	hypothetical protein	NA	472	654	520	1308	1428	1212	1705	3794	3230	326.395448	432.7585	336.909749	1872.77513	1621.87526	1528.27897	2723.41888	3865.58163	3405.9287	1790.43581	2.18049192	0.17980312	12.1271087	7.5882E-34	6.2687E-33
TS92_Nanopo	.	CDS	259787	259954	-	TS92_00251	TS92_00251	NA	hypothetical protein	NA	103	202	140	408	405	272	868	1682	678	71.2261253	133.665469	90.7064708	584.168391	459.98563	342.980099	1386.46779	1713.7344	714.928688	610.873674	2.17905958	0.31877367	6.83575771	8.1573E-12	2.5376E-11
TS92_Nanopo	.	CDS	2551779	2553371	-	TS92_02656	TS92_02656	NA	Multidrug efflux system permease protein	NA	919	928	931	1712	2588	2373	3764	4926	5656	635.503002	614.067107	603.198031	2451.21638	2939.36496	2992.24917	6012.28661	5018.93915	5964.06587	3025.65448	2.16966795	0.13498809	16.0730327	3.9431E-58	6.7786E-57
TS92_Nanopo	.	CDS	1065033	1065410	-	TS92_01096	gvpP	NA	gas vesicle protein	NA	3421	3300	3647	7086	9561	7979	7845	12595	13518	2365.67548	2183.64381	2362.90356	10145.6304	10859.0682	10061.1699	12530.9215	12832.6306	14254.2861	8621.76996	2.16296889	0.10644914	20.3192703	8.6858E-92	3.1122E-90
TS92_Nanopo	.	CDS	1957730	1958191	-	TS92_02040	TS92_02040	NA	hypothetical protein	NA	2660	2520	2492	5796	6332	5856	1566	1586	2002	1839.43197	1667.50982	1614.57518	8298.62743	7191.67656	7384.15978	2501.39236	1615.92316	2111.04312	3802.70438	2.14607893	0.16883524	12.711084	5.1318E-37	4.7994E-36
TS92_Nanopo	.	CDS	530730	532451	+	TS92_00508	abcA	NA	ABC superfamily ATP binding cassette%0Atransporter%2C ABC protein	NA	2078	2078	2276	4159	5624	5273	5760	9111	9197	1436.96979	1375.03389	1474.62805	5954.79494	6387.55354	6649.02229	9200.52361	9282.8978	9697.93384	5717.70642	2.14302035	0.09939632	21.5603596	4.233E-103	1.851E-101
TS92_Nanopo	.	CDS	1200997	1201659	+	TS92_01266	TS92_01266	NA	hypothetical protein	NA	8612	9470	10000	19322	26330	20068	24837	36024	35641	5955.3339	6266.39602	6479.03363	27664.955	29904.7448	25304.87	39672.4661	36703.667	37582.2616	23948.192	2.14244482	0.1089815	19.6587931	4.8616E-86	1.6619E-84
TS92_Nanopo	.	CDS	375789	376256	-	TS92_00359	TS92_00359	NA	phosphoenolpyruvate-dependent sugar%0Aphosphotransferase system EIIA 2	NA	162	204	132	296	488	404	124	190	120	112.025556	134.98889	85.5232439	423.80844	554.25429	509.426324	198.066828	193.584742	126.536051	259.801596	2.13936991	0.22623915	9.45623227	3.1924E-21	1.5693E-20
TS92_Nanopo	.	CDS	1922405	1922704	-	TS92_01993	TS92_01993	NA	hypothetical protein	NA	5	9	6	19	18	13	19	38	25	3.4575789	5.9553922	3.88742018	27.2039202	20.4438058	16.3924312	30.3489494	38.7169483	26.3616773	19.1964582	2.13612562	0.45858305	4.65809979	3.1914E-06	6.8579E-06
TS92_Nanopo	.	CDS	1654780	1655400	+	TS92_01720	lexA	NA	DNA damage-inducible repressor	NA	1608	1613	1832	3272	4905	3705	2933	4569	6546	1111.95737	1067.33862	1186.95896	4684.80141	5570.93707	4671.84289	4684.9194	4655.2036	6902.54158	3837.38899	2.13444979	0.1775232	12.0234977	2.6741E-33	2.1611E-32
TS92_Nanopo	.	CDS	2705759	2706220	-	TS92_02822	ctsR	NA	class III stress genes transcriptional%0Arepressor	NA	964	1268	839	2021	3046	2239	14135	34594	14140	666.621212	839.04859	543.590921	2893.63803	3459.54624	2823.28104	22578.021	35246.6871	14910.1647	9328.95543	2.1282366	0.27120193	7.84742439	4.2467E-15	1.5611E-14
TS92_Nanopo	.	CDS	983707	984750	+	TS92_00995	vraS	NA	integral membrane sensor signal transduction%0Ahistidine kinase	NA	1360	1632	1264	3093	3968	2847	4832	10535	9663	940.461461	1079.91112	818.949851	4428.51184	4506.72341	3589.94243	7718.21703	10733.7645	10189.3155	4889.53302	2.12700803	0.17640718	12.0573778	1.7734E-33	1.441E-32
TS92_Nanopo	.	CDS	2150947	2153544	-	TS92_02224	clpB	NA	ATPase subunit of an ATP-dependent protease%2C%0AClpB	NA	5338	6786	4512	11738	17444	11194	117270	321101	68782	3691.31123	4490.36572	2923.33997	16806.2955	19812.3193	14115.1442	187316.91	327159.232	72528.3555	72093.6971	2.12455417	0.37012418	5.74011174	9.4614E-09	2.4257E-08
TS92_Nanopo	.	CDS	2550738	2551094	-	TS92_02654	TS92_02654	NA	hypothetical protein	NA	724	993	781	1596	2177	1993	3131	4285	4584	500.657425	657.078273	506.012526	2285.12929	2472.56473	2513.0858	5001.1874	4365.84536	4833.67715	2570.58199	2.11823183	0.14432736	14.6765788	9.1071E-49	1.2091E-47
TS92_Nanopo	.	CDS	2143957	2145030	-	TS92_02217	oppD	NA	oligopeptide ABC superfamily ATP binding%0Acassette transporter%2C ABC protein	NA	13739	7939	15749	24529	33910	28854	17750	22774	26119	9500.7353	5253.31763	10203.8301	35120.2609	38513.8585	36383.6315	28352.308	23203.6785	27541.626	23785.9163	2.11782272	0.22153124	9.55992806	1.1784E-21	5.9499E-21
TS92_Nanopo	.	CDS	1153790	1154623	-	TS92_01220	TS92_01220	NA	hypothetical protein	NA	5588	6098	6217	12305	15110	13403	13199	19808	17994	3864.19018	4035.10907	4028.01521	17618.1178	17161.4392	16900.5966	21082.936	20181.7188	18974.0808	13760.6893	2.11140692	0.09333245	22.6224322	2.607E-113	1.337E-111
TS92_Nanopo	.	CDS	2416256	2417308	-	TS92_02521	TS92_02521	NA	hemin ABC superfamily ATP binding cassette%0Atransporter%2C permease component	NA	18	19	11	31	57	28	23	50	33	12.447284	12.5724946	7.12693699	44.3853434	64.7387183	35.3067749	36.7382019	50.9433531	34.797414	33.2285024	2.11085935	0.36731725	5.74669265	9.1006E-09	2.3393E-08
TS92_Nanopo	.	CDS	734595	734780	+	TS92_00722	rpsN_1	NA	30S ribosomal protein S14	NA	4447	6132	4990	10186	13856	11730	4032	4778	4714	3075.17067	4057.60722	3233.03778	14584.1648	15737.1874	14791.0168	6440.36653	4868.14682	4970.75787	7973.05065	2.1101344	0.1605739	13.1412042	1.9116E-39	1.9536E-38
TS92_Nanopo	.	CDS	733682	734002	+	TS92_00720	rplX	NA	50S ribosomal protein L24	NA	2386	3240	2678	5722	7333	5944	2182	2652	2460	1649.95665	2143.94119	1735.08521	8192.67532	8328.57932	7495.12393	3485.33724	2702.03545	2593.98904	4258.52482	2.10729189	0.16108801	13.0816186	4.1941E-39	4.2283E-38
TS92_Nanopo	.	CDS	1478795	1479433	+	TS92_01547	TS92_01547	NA	hypothetical protein	NA	4620	4762	5790	9722	13943	11040	9658	14278	16658	3194.8029	3151.06418	3751.36047	13919.8164	15835.9991	13920.957	15426.8502	14547.3839	17565.3128	11257.0608	2.1049888	0.1331903	15.80437	2.9028E-56	4.7695E-55
TS92_Nanopo	.	CDS	2423791	2424732	+	TS92_02529	speB	NA	Agmatinase	NA	577	326	650	1000	1314	1254	1348	3284	3098	399.004605	215.71754	421.137186	1431.78527	1492.39782	1581.23913	2153.17809	3345.95943	3266.73905	1589.68424	2.09286145	0.24757873	8.45331673	2.8314E-17	1.1599E-16
TS92_Nanopo	.	CDS	2555271	2557148	-	TS92_02659	licR_3	NA	putative licABCH operon regulator	NA	1270	1546	1537	2380	4386	3190	1822	3022	3382	878.22504	1023.00404	995.827469	3407.64895	4981.47401	4022.45043	2910.30452	3079.01626	3566.2077	2762.68427	2.08678211	0.16746247	12.4611928	1.2154E-35	1.0694E-34
TS92_Nanopo	.	CDS	1639619	1642327	-	TS92_01707	citB	NA	aconitate hydratase	NA	33989	31986	38040	72564	83676	76856	84722	100205	122790	23503.9298	21165.4639	24646.2439	103896.066	95036.4384	96912.0533	135327.563	102095.574	129478.014	81340.1496	2.08485852	0.14036235	14.8534026	6.6125E-50	9.1045E-49
TS92_Nanopo	.	CDS	721621	722478	-	TS92_00703	glcU	NA	DMT superfamily drug/metabolite transporter	NA	1842	2146	2167	4256	4580	4878	5452	9320	8351	1273.77207	1420.03018	1404.00659	6093.67811	5201.8128	6150.94457	8708.55117	9495.84102	8805.85468	5394.94347	2.08346533	0.11894896	17.5156247	1.0888E-68	2.5906E-67
TS92_Nanopo	.	CDS	735802	736164	+	TS92_00725	rplR	NA	50S ribosomal protein L18	NA	11826	16200	13610	26954	34802	30728	11062	11532	13666	8177.86561	10719.706	8817.96477	38592.3402	39526.9627	38746.6635	17669.4778	11749.575	14410.3473	20934.5448	2.06313846	0.17171671	12.0147798	2.9716E-33	2.395E-32
TS92_Nanopo	.	CDS	2417964	2418863	-	TS92_02523	alkA	NA	DNA-3-methyladenine glycosylase	NA	210	179	189	397	461	427	795	1486	964	145.218314	118.446134	122.453736	568.418753	523.588581	538.428317	1269.86394	1514.03645	1016.50628	646.328944	2.06311501	0.181707	11.3540753	7.0785E-30	4.9885E-29
TS92_Nanopo	.	CDS	1477250	1478770	+	TS92_01546	TS92_01546	NA	hypothetical protein	NA	10447	11806	11948	20694	31969	23420	19391	30710	29141	7224.26535	7812.15115	7741.14938	29629.3644	36309.3348	29531.5953	30973.4988	31289.4075	30728.2255	23470.9991	2.06118599	0.11996678	17.1813064	3.6656E-66	7.7868E-65
TS92_Nanopo	.	CDS	1043498	1043635	+	TS92_01078	TS92_01078	NA	hypothetical protein	NA	1358	1424	1561	2728	4062	2774	4998	9554	8704	939.078429	942.275388	1011.37715	3905.91022	4613.4855	3497.89263	7983.37101	9734.25591	9178.08156	4645.08087	2.04560679	0.14434493	14.1716566	1.3722E-45	1.6794E-44
TS92_Nanopo	.	CDS	645516	646961	-	TS92_00629	TS92_00629	NA	hypothetical protein	NA	256	249	292	619	503	599	158	233	239	177.02804	164.765851	189.187782	886.275083	571.290795	755.312792	252.375474	237.396025	252.017635	387.294386	2.04308406	0.18138887	11.263558	1.9858E-29	1.3831E-28
TS92_Nanopo	.	CDS	2444518	2445249	-	TS92_02553	TS92_02553	NA	ABC superfamily ATP binding cassette%0Atransporter%2C membrane protein	NA	310	266	272	642	602	597	246	341	366	214.369892	176.014925	176.229715	919.206144	683.731726	752.790879	392.939029	347.433668	385.934955	449.850104	2.04299118	0.16534145	12.3561946	4.5101E-35	3.8878E-34
TS92_Nanopo	.	CDS	259337	259765	-	TS92_00250	TS92_00250	NA	putative small heat shock protein	NA	951	1388	972	2336	3449	1700	6242	15509	4776	657.631507	918.453819	629.762069	3344.65039	3917.26034	2143.62562	9970.42854	15801.6093	5036.13483	4713.28404	2.04051214	0.33561017	6.0800068	1.2018E-09	3.267E-09
TS92_Nanopo	.	CDS	626324	626506	-	TS92_00610	TS92_00610	NA	hypothetical protein	NA	3890	4550	4149	7776	11260	8612	6891	10608	15160	2689.99638	3010.78161	2688.15105	11133.5623	12788.7363	10859.3552	11007.0848	10808.1418	15985.7211	8996.83672	2.03867477	0.17362585	11.7417695	7.7841E-32	5.9819E-31
TS92_Nanopo	.	CDS	732734	732949	+	TS92_00717	rpmC	NA	50S ribosomal protein L29	NA	1134	1649	1415	2482	3614	3081	974	1403	1181	784.178894	1091.16019	916.783259	3553.69104	4104.66189	3885.0062	1555.78299	1429.47049	1245.32563	2062.89562	2.03685067	0.15812411	12.8813416	5.733E-38	5.5357E-37
TS92_Nanopo	.	CDS	736700	736879	+	TS92_00727	rpmD	NA	50S ribosomal protein L30	NA	4736	6342	5600	9970	14709	11652	4732	5396	6256	3275.01873	4196.56637	3628.25883	14274.8992	16705.9966	14692.6622	7558.48572	5497.80667	6596.74612	8491.82671	2.02982386	0.15979645	12.7025594	5.7227E-37	5.3352E-36
TS92_Nanopo	.	CDS	642517	642978	-	TS92_00626	TS92_00626	NA	hypothetical protein	NA	252	312	312	509	811	580	1230	1872	1695	174.261977	206.453596	202.145849	728.778703	921.107027	731.354623	1964.69515	1907.31914	1787.32172	958.159753	2.02121009	0.15016887	13.4595808	2.7045E-41	2.9354E-40
TS92_Nanopo	.	CDS	1475790	1477163	+	TS92_01545	recQ	NA	ATP-dependent DNA helicase	NA	823	773	952	1360	2318	1852	1644	2641	3348	569.117487	511.502019	616.804002	1947.22797	2632.70788	2335.29097	2625.98278	2690.82791	3530.35582	1939.97965	2.01379808	0.17349518	11.6072279	3.7869E-31	2.8156E-30
TS92_Nanopo	.	CDS	1935478	1937259	-	TS92_02016	uvrC	NA	glycosyl transferase family protein	NA	2688	2680	2914	5120	6318	6193	11516	14794	19080	1858.79442	1773.38346	1887.9904	7330.74059	7175.77583	7809.10203	18394.658	15073.1193	20119.2321	9046.97734	2.00754252	0.13418659	14.9608281	1.3237E-50	1.8657E-49
TS92_Nanopo	.	CDS	1863982	1864566	-	TS92_01922	TS92_01922	NA	putative cell-division initiation protein	NA	5428	6297	6559	750	1082	549	732	1521	998	3753.54765	4166.78941	4249.59816	1073.83895	1228.89988	692.264979	1169.23321	1549.6968	1052.35816	2104.02524	-2.0020687	0.21852811	-9.161607	5.113E-20	2.376E-19
TS92_Nanopo	.	CDS	827626	828165	+	TS92_00830	atpD	NA	ATP synthase subunit delta	NA	5290	6098	6073	615	815	856	286	478	483	3658.11848	4035.10907	3934.71712	880.547942	925.650095	1079.37855	456.831554	487.018456	509.307605	1774.07543	-2.0038107	0.12581023	-15.927247	4.1001E-57	6.8122E-56
TS92_Nanopo	.	CDS	2596547	2596861	+	TS92_02703	TS92_02703	NA	thioredoxin	NA	37	70	50	9	9	1	6	11	7	25.5860839	46.3197171	32.3951681	12.8860674	10.2219029	1.26095625	9.58387876	11.2075377	7.38126964	17.4269535	-2.0045659	0.49614423	-4.0402886	5.3385E-05	0.0001033
TS92_Nanopo	.	CDS	1705694	1707538	-	TS92_01773	msbA_7	NA	Lipid A export ATP-binding/permease MsbA	NA	3369	3544	3460	410	474	461	188	281	270	2329.71666	2345.10111	2241.74564	587.031961	538.353552	581.30083	300.294868	286.301644	284.706115	1054.95026	-2.0150591	0.11059291	-18.220509	3.5484E-74	9.7711E-73
TS92_Nanopo	.	CDS	1856552	1857079	-	TS92_01915	pyrR	NA	bifunctional protein pyrimidine operon%0Aregulatory protein/uracil phosphoribosyltransfer	NA	2743	2538	2919	282	422	352	317	629	621	1896.82778	1679.4206	1891.22992	403.763446	479.293669	443.856599	506.348261	640.867382	654.824064	955.15908	-2.0319303	0.14764974	-13.761828	4.3245E-43	4.9088E-42
TS92_Nanopo	.	CDS	547663	548664	+	TS92_00525	arcB	NA	putative ornithine carbamoyltransferase	NA	274	358	278	25	57	34	22	74	56	189.475324	236.892268	180.117135	35.7946318	64.7387183	42.8725124	35.1408888	75.3961626	59.0501571	102.1642	-2.0340883	0.28549866	-7.1246861	1.0432E-12	3.4281E-12
TS92_Nanopo	.	CDS	435039	436793	+	TS92_00409	lytS	NA	autolysis histidine kinase LytS	NA	909	916	946	99	137	115	75	120	121	628.587844	606.126584	612.916581	141.746742	155.600077	145.009968	119.798485	122.264047	127.590518	295.51565	-2.0519471	0.14373229	-14.276173	3.0806E-46	3.8174E-45
TS92_Nanopo	.	CDS	1333122	1333790	+	TS92_01385	yrrB	NA	TPR repeat-containing protein YrrB	NA	654	766	624	82	109	65	60	143	84	452.25132	506.870047	404.291698	117.406392	123.798602	81.962156	95.8387876	145.69799	88.5752357	224.076914	-2.0541439	0.22944871	-8.9525191	3.4746E-19	1.5633E-18
TS92_Nanopo	.	CDS	923083	923301	+	TS92_00932	TS92_00932	NA	hypothetical protein	NA	1124	1227	1375	135	143	187	223	409	378	777.263737	811.91847	890.867124	193.291012	162.414679	235.798818	356.200827	416.716628	398.58856	471.451095	-2.0579968	0.16621593	-12.381465	3.2928E-35	2.855E-34
TS92_Nanopo	.	CDS	940643	941224	-	TS92_00954	hisH	NA	imidazole glycerol phosphate synthase subunit%0AhisH	NA	295	234	348	30	39	40	15	30	22	203.997155	154.840197	225.47037	42.9535581	44.2949125	50.4382499	23.9596969	30.5660119	23.198276	88.8576031	-2.0616378	0.24651227	-8.3632261	6.1026E-17	2.4526E-16
TS92_Nanopo	.	CDS	2036216	2037541	-	TS92_02122	mshA	NA	D-inositol-3-phosphate glycosyltransferase	NA	456	469	483	49	62	64	45	82	61	315.331196	310.342105	312.937324	70.1574783	70.4175532	80.7011998	71.8790907	83.5470991	64.3224926	153.292838	-2.07085	0.18402323	-11.253199	2.2334E-29	1.5447E-28
TS92_Nanopo	.	CDS	2201897	2203117	-	TS92_02273	dltB	NA	D-alanine membrane transfer protein	NA	7978	8519	8140	866	1168	1042	422	660	594	5516.91289	5637.10957	5273.93337	1239.92604	1326.57584	1313.91641	674.06614	672.452261	626.353452	2475.694	-2.0770134	0.10173275	-20.416369	1.1963E-92	4.3924E-91
TS92_Nanopo	.	CDS	1950422	1951567	+	TS92_02030	tmcAL	NA	tRNA(Met) cytidine acetate ligase	NA	333	387	378	39	39	56	28	56	64	230.274755	256.081865	244.907471	55.8396256	44.2949125	70.6135498	44.7247676	57.0565555	67.4858938	119.031044	-2.0833012	0.22231007	-9.3711508	7.1746E-21	3.4638E-20
TS92_Nanopo	.	CDS	236621	237445	+	TS92_00228	TS92_00228	NA	tetracenomycin polyketide synthesis%0AO-methyltransferase%2C TcmP	NA	15148	21907	13700	2143	2378	1670	1536	2174	1738	10475.081	14496.0863	8876.27607	3068.31584	2700.8539	2105.79693	2453.47296	2215.01699	1832.6638	5358.17376	-2.0835715	0.21357924	-9.7554963	1.7474E-22	9.1334E-22
TS92_Nanopo	.	CDS	1386198	1387013	+	TS92_01443	yqfL	NA	Putative pyruvate%2C phosphate dikinase%0Aregulatory protein	NA	1182	1210	1276	112	181	162	125	228	195	817.371652	800.669396	826.724691	160.35995	205.573825	204.274912	199.664141	232.30169	205.621083	405.840149	-2.0860124	0.14925863	-13.975825	2.1896E-44	2.5739E-43
TS92_Nanopo	.	CDS	2522007	2522477	-	TS92_02629	TS92_02629	NA	hypothetical protein	NA	218	213	207	25	30	23	18	28	26	150.75044	140.944282	134.115996	35.7946318	34.0730096	29.0019937	28.7516363	28.5282777	27.4161444	67.7084902	-2.0861745	0.24217428	-8.6143522	7.0338E-18	2.9884E-17
TS92_Nanopo	.	CDS	1514622	1514951	+	TS92_01584	TS92_01584	NA	hypothetical protein	NA	1227	1273	1334	141	152	176	190	262	263	848.489862	842.357141	864.303086	201.881723	172.636582	221.928299	303.489494	266.94317	277.324845	444.372689	-2.0927293	0.14391105	-14.541825	6.5815E-48	8.4733E-47
TS92_Nanopo	.	CDS	2204590	2204733	-	TS92_02275	TS92_02275	NA	hypothetical protein	NA	3630	3653	3712	446	401	482	398	922	779	2510.20228	2417.22752	2405.01728	638.576231	455.442562	607.780911	635.730624	939.395431	821.429864	1270.08919	-2.0943975	0.18033345	-11.614027	3.4975E-31	2.62E-30
TS92_Nanopo	.	CDS	1217593	1218222	+	TS92_01279	plsC	NA	1-acyl-sn-glycerol-3-phosphate acyltransferase	NA	2358	2710	2523	286	349	289	113	321	204	1630.59421	1793.23476	1634.66018	409.490588	396.382679	364.416355	180.496383	327.056327	215.111287	772.38253	-2.095395	0.19622225	-10.678682	1.2808E-26	8.019E-26
TS92_Nanopo	.	CDS	1221812	1222969	-	TS92_01283	TS92_01283	NA	putative soluble hydrogenase subunit	NA	4302	3374	4582	435	558	507	166	297	306	2974.90089	2232.61036	2968.69321	622.826593	633.757979	639.304817	265.153979	302.603517	322.66693	1218.05759	-2.0981439	0.15472879	-13.560139	6.9005E-42	7.5728E-41
TS92_Nanopo	.	CDS	2103703	2105388	-	TS92_02182	TS92_02182	NA	Serine protease HtrA-like protein	NA	7508	8247	8123	936	1071	902	362	782	638	5191.90048	5457.12439	5262.91902	1340.15101	1216.40644	1137.38253	578.227352	796.754042	672.750004	2405.95725	-2.0984782	0.14228823	-14.74808	3.1654E-49	4.2405E-48
TS92_Nanopo	.	CDS	2096240	2096605	-	TS92_02174	TS92_02174	NA	hypothetical protein	NA	25	28	21	3	3	2	4	7	7	17.2878945	18.5278868	13.6059706	4.29535581	3.40730096	2.52191249	6.38925621	7.13206943	7.38126964	8.9498792	-2.1014117	0.58554025	-3.5888424	0.00033215	0.00059373
TS92_Nanopo	.	CDS	1360880	1361335	+	TS92_01416	comEB	NA	late competence operon required for DNA binding%0Aand uptake comEB	NA	2149	2322	2345	250	290	284	141	328	321	1486.06741	1536.49119	1519.33339	357.946318	329.372426	358.111574	225.221151	334.188396	338.483936	720.579532	-2.1086173	0.16199636	-13.016449	9.8653E-39	9.8455E-38
TS92_Nanopo	.	CDS	2748188	2748451	-	TS92_02868	veg	NA	Protein Veg	NA	5221	5981	5979	556	949	591	2612	4965	4222	3610.40389	3957.68897	3873.81421	796.072611	1077.84287	745.225142	4172.18189	5058.67496	4451.96006	3082.65162	-2.1137301	0.16372533	-12.910221	3.9417E-38	3.8561E-37
TS92_Nanopo	.	CDS	467087	468196	-	TS92_00439	thiO	NA	Glycine oxidase	NA	7981	4966	8138	685	408	1213	940	690	1666	5518.98744	3286.05307	5272.63757	980.772911	463.392931	1529.53993	1501.47434	703.018273	1756.74217	2334.7354	-2.1206375	0.49762501	-4.2615171	2.0304E-05	4.0801E-05
TS92_Nanopo	.	CDS	561768	562418	+	TS92_00540	TS92_00540	NA	hypothetical protein	NA	2348	2415	2572	236	354	292	267	432	518	1623.67905	1598.03024	1666.40745	337.901324	402.061514	368.199224	426.482605	440.150571	546.213953	823.236215	-2.1300714	0.14299284	-14.89635	3.4808E-50	4.8374E-49
TS92_Nanopo	.	CDS	2123287	2124147	-	TS92_02198	rluA1	NA	RluD subfamily ribosomal large subunit%0Apseudouridine synthase	NA	752	832	860	88	108	94	72	158	162	520.019866	550.542923	557.196892	125.997104	122.662835	118.529887	115.006545	160.980996	170.823669	271.306746	-2.1361122	0.17298976	-12.348201	4.9813E-35	4.2816E-34
TS92_Nanopo	.	CDS	2278156	2278509	-	TS92_02371	TS92_02371	NA	hypothetical protein	NA	48	46	53	8	3	5	6	6	14	33.1927574	30.4386712	34.3388782	11.4542822	3.40730096	6.30478123	9.58387876	6.11320237	14.7625393	16.6218102	-2.1478388	0.47576677	-4.5144784	6.3473E-06	1.3331E-05
TS92_Nanopo	.	CDS	1556794	1558680	+	TS92_01627	norD	NA	nitric oxide reductase activation protein NorD	NA	3856	3788	3890	382	522	465	155	276	298	2666.48485	2506.55841	2520.34408	546.941974	592.870367	586.344655	247.583535	281.207309	314.231193	1140.28515	-2.1485658	0.12440938	-17.270128	7.8968E-67	1.7396E-65

TS92_Nanopo	.	CDS	2249473	2249979	-	TS92_02329	TS92_02329	NA	hypothetical protein	NA	182	210	203	26	28	15	16	17	17	125.855872	138.959151	131.524383	37.226417	31.8014756	18.9143437	25.55701	17.32074	17.9259405	60.565037	-2.1503091	0.27339139	-7.865314	3.6817E-15	1.3602E-14
TS92_Nanopo	.	CDS	1555986	1556783	+	TS92_01626	TS92_01626	NA	ATPase	NA	2131	2087	2124	234	265	247	98	146	134	1473.62013	1380.98928	1376.14674	335.037753	300.978252	311.456193	156.536686	148.754591	141.29859	624.979802	-2.1540439	0.12616462	-17.07328	2.3464E-65	4.8798E-64
TS92_Nanopo	.	CDS	944661	945491	-	TS92_00959	hisZ	NA	ATP phosphoribosyltransferase regulatory%0Asubunit	NA	175	130	160	22	13	18	17	28	22	121.015261	86.0223318	103.664538	31.499276	14.7649708	22.6972124	27.1543232	28.5282777	23.198276	50.9493853	-2.1541137	0.30773164	-6.9999747	2.5601E-12	8.143E-12
TS92_Nanopo	.	CDS	809856	810383	-	TS92_00808	TS92_00808	NA	hypothetical protein	NA	190	215	195	24	19	26	26	46	52	131.387998	142.267703	126.341156	34.3628465	21.5795728	32.7848624	41.5301413	46.8678848	54.8322887	70.2171615	-2.1578247	0.25698185	-8.396798	4.5882E-17	1.8616E-16
TS92_Nanopo	.	CDS	1244563	1245489	+	TS92_01308	TS92_01308	NA	exopolyphosphatase-like protein	NA	1819	1708	1882	182	199	248	102	150	200	1257.8672	1130.2011	1219.35413	260.584919	226.017631	312.717149	162.925939	152.830059	210.893418	548.154616	-2.1621886	0.17316728	-12.486127	8.8876E-36	7.8666E-35
TS92_Nanopo	.	CDS	477978	479597	+	TS92_00450	TS92_00450	NA	hypothetical protein	NA	2000	2464	2203	208	355	217	410	947	884	1383.03156	1630.45404	1427.33111	297.811336	403.197281	273.627506	654.898382	964.867107	932.148909	885.263026	-2.1671776	0.19977163	-10.848275	2.0322E-27	1.3139E-26
TS92_Nanopo	.	CDS	2748551	2749441	-	TS92_02869	ksgA	NA	dimethyladenosine transferase	NA	2150	2410	2134	226	291	264	137	277	230	1486.75893	1594.72169	1382.62578	323.583471	330.508193	332.892449	218.831898	282.226176	242.527431	688.297335	-2.1684851	0.13958396	-15.535345	1.9999E-54	3.114E-53
TS92_Nanopo	.	CDS	1885354	1885629	+	TS92_01945	TS92_01945	NA	hypothetical protein	NA	243	276	242	22	39	26	22	27	36	168.038335	182.632027	156.792614	31.499276	44.2949125	32.7848624	35.1408888	27.5094107	37.9608153	79.6281268	-2.1890083	0.24297683	-9.0091235	2.0771E-19	9.431E-19
TS92_Nanopo	.	CDS	357850	359214	+	TS92_00340	ribZ	NA	Riboflavin transporter RibZ	NA	459	582	561	48	54	78	136	174	236	317.405743	385.115362	363.473787	68.725693	61.3314173	98.3545872	217.234585	177.282869	248.854233	215.308697	-2.2009653	0.21831386	-10.081656	6.6594E-24	3.6541E-23
TS92_Nanopo	.	CDS	1505507	1508200	+	TS92_01577	dinG	NA	DNA polymerase III%2C epsilon%0Asubunit/ATP-dependent helicase DinG	NA	4152	3615	4320	400	493	484	238	354	349	2871.17352	2392.08253	2798.94253	572.714108	559.933125	610.302823	380.160524	360.67894	368.009015	1212.66635	-2.2026165	0.12671354	-17.382645	1.1168E-67	2.5549E-66
TS92_Nanopo	.	CDS	2039690	2040502	-	TS92_02125	tagG_1	NA	teichoic acid ABC superfamily ATP binding%0Acassette transporter%2C membrane protei	NA	188	194	202	23	22	20	14	30	44	130.004967	128.371787	130.876479	32.9310612	24.9868737	25.2191249	22.3623838	30.5660119	46.396552	63.5239157	-2.2029798	0.27753817	-7.9375741	2.0617E-15	7.6934E-15
TS92_Nanopo	.	CDS	2252582	2253070	-	TS92_02333	TS92_02333	NA	hypothetical protein	NA	342	380	408	37	52	38	30	85	47	236.498397	251.449893	264.344572	52.976055	59.0598833	47.9163374	47.9193938	86.6037002	49.5599533	121.814243	-2.2052719	0.2417558	-9.1218988	7.3819E-20	3.4143E-19
TS92_Nanopo	.	CDS	1438730	1440598	+	TS92_01506	tktA_1	NA	transketolase	NA	9789	8921	10299	978	1156	1144	763	877	1055	6769.24797	5903.11709	6672.75673	1400.286	1312.94664	1442.53395	1218.74992	893.546413	1112.46278	2969.51639	-2.2093855	0.14394859	-15.348434	3.6284E-53	5.4226E-52
TS92_Nanopo	.	CDS	1149283	1149738	+	TS92_01214	TS92_01214	NA	hypothetical protein	NA	2208	1871	2102	241	227	224	102	220	209	1526.86684	1238.05987	1361.89287	345.06025	257.819106	282.454199	162.925939	224.150754	220.383622	624.401494	-2.2097887	0.17548961	-12.592134	2.3328E-36	2.1347E-35
TS92_Nanopo	.	CDS	1682298	1682933	+	TS92_01752	TS92_01752	NA	maltose O-acetyltransferase	NA	709	768	864	71	115	79	78	123	100	490.284688	508.193468	559.788506	101.656754	130.613204	99.6155435	124.590424	125.320649	105.446709	249.501105	-2.2121278	0.17479327	-12.65568	1.0407E-36	9.6419E-36
TS92_Nanopo	.	CDS	1722595	1722972	-	TS92_01791	nrmA_2	NA	putative NmrA negative transcriptional%0Aregulator family protein	NA	4	7	8	0	1	0	2	3	1	2.76606312	4.63197171	5.1832269	0	1.13576699	0	3.19462625	3.05660119	1.05446709	2.33585814	-2.2129534	0.9535532	-2.3207446	0.02030063	0.02923684
TS92_Nanopo	.	CDS	2079573	2080574	+	TS92_02153	TS92_02153	NA	putative acetyltransferase	NA	1559	1391	1590	183	171	151	105	183	118	1078.0731	920.43895	1030.16635	262.016705	194.216155	190.404393	167.717878	186.452672	124.427117	461.545924	-2.215122	0.18993845	-11.662315	1.9857E-31	1.5065E-30
TS92_Nanopo	.	CDS	991674	991835	+	TS92_01006	TS92_01006	NA	hypothetical protein	NA	588	630	568	58	68	74	46	102	77	406.611279	416.877454	368.00911	83.0435457	77.2321551	93.3107622	73.4764038	103.92444	81.193966	189.29768	-2.2182545	0.18798448	-11.8002	3.8938E-32	3.0315E-31
TS92_Nanopo	.	CDS	821431	821868	+	TS92_00821	lacB_2	NA	galactose-6-phosphate isomerase LacB subunit	NA	15604	14229	14830	1418	1896	1714	976	1737	2220	10790.4122	9415.47507	9608.40687	2030.27151	2153.41421	2161.27901	1558.97761	1769.77209	2340.91694	4647.65839	-2.219858	0.1623011	-13.677406	1.3855E-42	1.5549E-41
TS92_Nanopo	.	CDS	953972	956152	+	TS92_00969	pcrA	NA	ATP-dependent DNA helicase%2C PcrA	NA	4640	4285	4386	482	493	510	317	672	578	3208.63322	2835.4284	2841.70415	690.120501	559.933125	643.086786	506.348261	684.678665	609.481979	1397.71289	-2.2212561	0.15224726	-14.589794	3.2618E-48	4.2547E-47
TS92_Nanopo	.	CDS	684593	685447	+	TS92_00667	TS92_00667	NA	ROK family protein	NA	333	376	352	36	40	42	32	72	54	230.274755	248.803052	228.061984	51.5442698	45.4306795	52.9601624	51.1140201	73.3584284	56.9412229	115.387619	-2.2216485	0.21246176	-10.456698	1.3653E-25	8.1699E-25
TS92_Nanopo	.	CDS	2811661	2812929	-	TS92_02934	pbuX	NA	NCS2 nucleobase cation symporter%2C PbuX	NA	15454	16224	13686	1602	1740	1687	730	1070	950	10686.6849	10735.587	8867.20543	2293.72	1976.23456	2127.23319	1166.03858	1090.18776	1001.74374	4438.29279	-2.2354983	0.13239248	-16.885387	5.7637E-64	1.1504E-62
TS92_Nanopo	.	CDS	2543450	2544199	+	TS92_02648	TS92_02648	NA	phosphosugar-binding transcriptional regulator	NA	624	617	726	71	79	65	43	60	68	431.505847	408.275221	470.377841	101.656754	89.725592	81.962156	68.6844645	61.1320237	71.7037622	198.335962	-2.2517217	0.17670934	-12.742516	3.4315E-37	3.2295E-36
TS92_Nanopo	.	CDS	895564	896334	-	TS92_00902	TS92_00902	NA	putative metal-dependent membrane protease	NA	1505	1471	1598	171	147	172	63	151	112	1040.73125	973.37577	1035.34957	244.835281	166.957747	216.884474	100.630727	153.848926	118.100314	450.07934	-2.266715	0.1867483	-12.137808	6.6586E-34	5.5161E-33
TS92_Nanopo	.	CDS	1806615	1807601	-	TS92_01868	plsX	NA	glycerol-3-phosphate acyltransferase PlsX	NA	4295	4964	4762	428	531	560	210	394	364	2970.06027	3284.72965	3085.31581	612.804096	603.09227	706.135498	335.435757	401.433622	383.826021	1375.87033	-2.2731906	0.12877385	-17.652579	9.7214E-70	2.3698E-68
TS92_Nanopo	.	CDS	2391254	2391700	+	TS92_02493	TS92_02493	NA	MarR family transcriptional regulator	NA	337	442	436	37	47	45	57	156	84	233.040818	292.475928	282.485866	52.976055	53.3810484	56.7430311	91.0468482	158.943262	88.5752357	145.158677	-2.2765172	0.2555191	-8.9093818	5.1318E-19	2.3054E-18
TS92_Nanopo	.	CDS	1621068	1621685	+	TS92_01693	TS92_01693	NA	hypothetical protein	NA	2030	1982	2236	198	225	244	118	296	242	1403.77703	1311.5097	1448.71192	283.493484	255.547572	307.673324	188.482949	301.58465	255.181036	639.551297	-2.2846543	0.17443907	-13.097148	3.4186E-39	3.4582E-38
TS92_Nanopo	.	CDS	800723	801520	-	TS92_00799	panK	NA	pantothenate kinase	NA	1104	1238	1238	115	144	125	96	152	142	763.433421	819.197283	802.104363	164.655306	163.550446	157.619531	153.34206	154.867799	149.734327	369.833837	-2.2871995	0.13828798	-16.539395	1.9093E-61	3.7605E-60
TS92_Nanopo	.	CDS	1878026	1879642	-	TS92_01937	bshC	NA	Putative cysteine ligase BshC	NA	3830	4143	4773	401	440	512	688	1156	1473	2648.50544	2741.46554	3092.44275	574.145894	499.737474	645.609598	1098.95143	1177.81032	1553.23003	1559.09983	-2.2887174	0.17194087	-13.311073	1.996E-40	2.0975E-39
TS92_Nanopo	.	CDS	2082463	2084061	+	TS92_02156	rocB	NA	Protein RocB	NA	2724	2416	2790	264	272	294	75	74	92	1883.68898	1598.69195	1807.65038	377.991312	308.928621	370.721136	119.798485	75.3961626	97.0109724	737.764223	-2.3113482	0.16736868	-13.809921	2.2207E-43	2.5305E-42
TS92_Nanopo	.	CDS	1361351	1363663	+	TS92_01417	comEC	NA	competence protein ComEC	NA	632	558	497	64	59	52	72	107	71	437.037973	369.234316	322.007971	91.6342573	67.0102523	65.5697248	115.006545	109.018776	74.8671635	183.487442	-2.3131466	0.22824863	-10.134329	3.8904E-24	2.1586E-23
TS92_Nanopo	.	CDS	2754247	2754495	-	TS92_02875	TS92_02875	NA	putative GIY-YIG nuclease	NA	224	246	266	30	16	28	23	56	38	154.899535	162.78072	172.342295	42.9535581	18.1722718	35.3067749	36.7382019	57.0565555	40.0697495	80.0355179	-2.3291401	0.28213603	-8.2553797	1.5142E-16	5.9409E-16
TS92_Nanopo	.	CDS	159734	160087	+	TS92_00152	TS92_00152	NA	hypothetical protein	NA	12	19	20	2	2	0	6	11	6	8.29818936	12.5724946	12.9580673	2.86375054	2.27153397	0	9.58387876	11.2075377	6.32680255	7.34245275	-2.3378644	0.70228791	-3.3289259	0.00087182	0.00149268
TS92_Nanopo	.	CDS	97854	99002	+	TS92_00094	xylR_1	NA	xylose operon repressor	NA	196	235	219	20	26	20	27	50	25	135.537093	155.501907	141.890836	28.6357054	29.5299417	25.2191249	43.1274544	50.9433531	26.3616773	70.7496771	-2.3395407	0.27536938	-8.4960089	1.9622E-17	8.0826E-17
TS92_Nanopo	.	CDS	2726465	2727256	-	TS92_02847	folP	NA	dihydropteroate synthase chain A synthetase	NA	4186	3842	4056	417	366	445	580	1110	756	2894.68505	2542.29076	2627.89604	597.054458	415.690717	561.12553	926.441614	1130.94244	797.177121	1388.14486	-2.3426221	0.18203524	-12.869058	6.7217E-38	6.4694E-37
TS92_Nanopo	.	CDS	1803835	1804458	-	TS92_01864	TS92_01864	NA	hypothetical protein	NA	184	173	154	19	14	18	7	15	19	127.238903	114.475872	99.7771179	27.2039202	15.9007378	22.6972124	11.1811919	15.2830059	20.0348747	50.4214263	-2.3543364	0.30062691	-7.8314224	4.8238E-15	1.7602E-14
TS92_Nanopo	.	CDS	2373795	2374397	-	TS92_02473	pabA	NA	para-aminobenzoate synthase%2C glutamine%0Aamidotransferase%2C component II	NA	292	291	373	30	36	30	28	50	39	201.922608	192.557681	241.667954	42.9535581	40.8876115	37.8286874	44.7247676	50.9433531	41.1242166	99.4011597	-2.3645147	0.22934239	-10.309977	6.3517E-25	3.7039E-24
TS92_Nanopo	.	CDS	709120	710160	-	TS92_00692	hldD	NA	ADP-L-glycero-D-manno-heptose-6-epimerase	NA	1956	2018	2123	168	284	164	201	534	314	1352.60487	1335.33127	1375.49884	240.539926	322.557824	206.796824	321.059938	544.075011	331.102667	669.951908	-2.3716986	0.22821532	-10.392372	2.6859E-25	1.5849E-24
TS92_Nanopo	.	CDS	2333356	2334411	-	TS92_02436	TS92_02436	NA	glycosyl transferase family protein	NA	2765	2670	2955	272	308	263	219	420	303	1912.04113	1766.76635	1914.55444	389.445594	349.816232	331.631493	349.811575	427.924166	319.503529	862.388279	-2.3754883	0.14854964	-15.991209	1.4715E-57	2.4724E-56
TS92_Nanopo	.	CDS	1223588	1224352	+	TS92_01285	TS92_01285	NA	glycerophosphoryl diester phosphodiesterase	NA	423	432	481	44	49	40	46	114	114	292.511175	285.858826	311.641518	62.9985519	55.6525824	50.4382499	73.4764038	116.150845	120.209248	152.104156	-2.3762283	0.21831102	-10.884601	1.365E-27	8.8632E-27
TS92_Nanopo	.	CDS	939946	940650	-	TS92_00953	hisA	NA	phosphoribosylformimino-5-aminoimidazole%0Acarboxamide ribotide isomerase	NA	485	339	540	46	49	39	23	30	59	335.385153	224.319773	349.867816	65.8621225	55.6525824	49.1772936	36.7382019	30.5660119	62.2135584	134.420279	-2.3806453	0.27754926	-8.5773794	9.7059E-18	4.077E-17
TS92_Nanopo	.	CDS	227355	228446	+	TS92_00218	TS92_00218	NA	cysteine desulfurase	NA	86	82	104	9	11	6	11	29	16	59.4703571	54.26024	67.3819497	12.8860674	12.4934369	7.56573748	17.5704444	29.5471448	16.8714735	30.8940946	-2.3862457	0.37824971	-6.3086519	2.8148E-10	7.9649E-10
TS92_Nanopo	.	CDS	1744652	1745563	-	TS92_01810	truB	NA	putative tRNA pseudouridine synthase B	NA	2675	2521	2928	250	275	282	278	575	534	1849.80471	1668.17153	1897.06105	357.946318	312.335922	355.589662	444.053049	585.84856	563.085427	892.655136	-2.3903537	0.15002066	-15.933497	3.7102E-57	6.1989E-56
TS92_Nanopo	.	CDS	2637801	2638223	+	TS92_02750	yeiL	NA	Regulatory protein YeiL	NA	129	120	182	14	10	17	22	42	54	89.2055356	79.4052293	117.918412	20.0449938	11.3576699	21.4362562	35.1408888	42.7924166	56.9412229	52.693625	-2.3954095	0.33333696	-7.1861504	6.6644E-13	2.2071E-12
TS92_Nanopo	.	CDS	1343838	1344761	+	TS92_01394	TS92_01394	NA	peptidase U32 family protein	NA	2956	2862	2734	305	299	235	220	395	262	2044.12065	1893.81472	1771.36779	436.694508	339.594329	296.324718	351.408888	402.452499	276.270378	868.005385	-2.3967637	0.18735483	-12.792644	1.8024E-37	1.7125E-36
TS92_Nanopo	.	CDS	1802948	1803667	-	TS92_01863	rnc	NA	putative ribonuclease III	NA	490	505	554	45	70	38	79	162	104	338.842732	334.163673	358.933683	64.4303372	79.5036891	47.9163374	126.187737	165.056464	109.664577	180.522668	-2.3968625	0.22344086	-10.727055	7.5979E-27	4.7873E-26
TS92_Nanopo	.	CDS	1943817	1944743	+	TS92_02023	rnhC	NA	ribonuclease HIII	NA	742	731	949	72	104	62	46	80	79	513.104709	483.710189	614.860291	103.08854	118.119767	78.1792873	73.4764038	81.5093649	83.3029002	238.816828	-2.4078948	0.19614952	-12.275813	1.2216E-34	1.035E-33
TS92_Nanopo	.	CDS	751376	751519	+	TS92_00749	TS92_00749	NA	hypothetical protein	NA	132	158	131	11	11	18	10	28	33	91.2800829	104.550219	84.8753405	15.749638	12.4934369	22.6972124	15.9731313	28.5282777	34.797414	45.660528	-2.4124847	0.33149101	-7.2776775	3.3962E-13	1.1438E-12
TS92_Nanopo	.	CDS	2606844	2608148	+	TS92_02717	moxC_2	NA	Putative monooxygenase MoxC	NA	563	752	604	60	68	59	48	78	78	389.323384	497.606104	391.333631	85.9071163	77.2321551	74.3964185	76.6710301	79.4716308	82.2484331	194.909989	-2.413657	0.1923424	-12.548751	4.0383E-36	3.6393E-35
TS92_Nanopo	.	CDS	1952074	1952628	-	TS92_02032	TS92_02032	NA	site-specific DNA-methyltransferase%0A(adenine-specific)	NA	1491	1591	1736	117	205	154	181	332	312	1031.05003	1052.781	1124.76024	167.518877	232.832232	194.187262	289.113676	338.263864	328.993732	528.833434	-2.4143664	0.15570321	-15.506208	3.1494E-54	4.853E-53
TS92_Nanopo	.	CDS	719040	719207	-	TS92_00699	TS92_00699	NA	hypothetical protein	NA	1064	1198	1134	102	132	97	74	207	158	735.77279	792.728873	734.722414	146.042098	149.921242	122.312756	118.201171	210.905482	166.6058	353.023625	-2.4158119	0.19981416	-12.090294	1.1886E-33	9.765E-33
TS92_Nanopo	.	CDS	468189	468773	-	TS92_00440	tenI	NA	Thiazole tautomerase	NA	5772	3528	5381	394	215	672	446	336	899	3991.42908	2334.51374	3486.368	564.123397	244.189902	847.362598	712.401655	342.339333	947.965915	1496.74374	-2.4162036	0.51662456	-4.6769043	2.9124E-06	6.2777E-06
TS92_Nanopo	.	CDS	1546056	1546730	-	TS92_01616	arlR_1	NA	response regulator receiver	NA	1970	1973	2153	182	225	190	84	170	131	1362.28609	1305.55431	1394.93594	260.584919	255.547572	239.581687	134.174303	173.2074	138.135189	584.889712	-2.4177658	0.14042809	-17.21711	1.9761E-66	4.2898E-65
TS92_Nanopo	.	CDS	269462	271264	-	TS92_00262	TS92_00262	NA	O-acetyltransferase	NA	1861	1535	1767	149	207	147	183	278	185	1286.91087	1015.72523	1144.84524	213.336005	235.103766	185.360568	292.308302	283.245043	195.076412	539.10127	-2.4240434	0.19085926	-12.700685	5.8614E-37	5.4475E-36
TS92_Nanopo	.	CDS	918516	919553	-	TS92_00927	TS92_00927	NA	acyl-coenzyme A:6-aminopenicillanic acid%0Aacyl-transferase	NA	1107	1072	1312	94	102	139	121	218	298	765.507968	709.353382	850.049212	134.587815	115.848233	175.272918	193.274888	222.113019	314.231193	386.693181	-2.4262882	0.21749866	-11.155417	6.7376E-29	4.5334E-28
TS92_Nanopo	.	CDS	1876502	1877437	-	TS92_01935	mraW	NA	S-adenosyl-methyltransferase MraW	NA	11980	10788	15326	1034	1424	1218	2458	5694	5078	8284.35904	7138.53012	9929.76694	1480.46597	1617.33219	1535.84471	3926.19567	5801.42905	5354.58389	5007.61195	-2.4347045	0.18099777	-13.451572	3.014E-41	3.2595E-40
TS92_Nanopo	.	CDS	1429986	1430816	+	TS92_01494	TS92_01494	NA	lipoate-protein ligase A family protein	NA	1730	1845	2036	163	195	181	155	376	309	1196.3223	1220.8554	1319.13125	233.380999	221.474563	228.233081	247.583535	383.094015	325.830331	597.32283	-2.4377155	0.16915722	-14.410946	4.4162E-47	5.6127E-46
TS92_Nanopo	.	CDS	1385536	1386183	+	TS92_01442	ccpN	NA	Transcriptional repressor CcpN	NA	2145	2110	2170	178	212	218	226	458	338	1483.30135	1396.20862	1405.9503	254.857778	240.782601	274.888462	360.992767	466.641114	356.409877	693.336984	-2.464489	0.15110752	-16.309505	8.4474E-60	1.5319E-58
TS92_Nanopo	.	CDS	350048	350536	+	TS92_00331	TS92_00331	NA	hypothetical protein	NA	327	272	326	28	36	22	43	85	50	226.12566	179.985187	211.216496	40.0899876	40.8876115	27.7410374	68.6844645	86.6037002	52.7233546	103.784167	-2.4706012	0.25762465	-9.5899253	8.8149E-22	4.4966E-21
TS92_Nanopo	.	CDS	1674096	1674689	-	TS92_01739	TS92_01739	NA	hypothetical protein	NA	2737	2558	2676	194	269	283	204	468	620	1892.67869	1692.65481	1733.7894	277.766343	305.52132	356.850618	325.851878	476.829785	653.769596	857.301381	-2.4732077	0.22388559	-11.046748	2.273E-28	1.5055E-27
TS92_Nanopo	.	CDS	1590202	1590720	+	TS92_01661	TS92_01661	NA	hypothetical protein	NA	99	134	113	12	10	9	7	13	13	68.4600622	88.6691728	73.21308	17.1814233	11.3576699	11.3486062	11.1811919	13.2452718	13.7080722	34.2627278	-2.4911215	0.34757929	-7.1670597	7.6626E-13	2.5321E-12
TS92_Nanopo	.	CDS	2595459	2596238	-	TS92_02701	TS92_02701	NA	hypothetical protein	NA	205	226	201	22	22	13	16	32	15	141.760735	149.546515	130.228576	31.499276	24.9868737	16.3924312	25.55701	32.603746	15.8170064	63.1546855	-2.499249	0.2960026	-8.4433347	3.0841E-17	1.2616E-16
TS92_Nanopo	.	CDS	1474853	1475809	+	TS92_01544	TS92_01544	NA	hypothetical protein	NA	322	339	456	28	36	38	16	26	28	222.668081	224.319773	295.443933	40.0899876	40.8876115	47.9163374	25.55701	26.4905436	29.5250786	105.877595	-2.5004211	0.23637456	-10.578216	3.7605E-26	2.3155E-25
TS92_Nanopo	.	CDS	177819	179636	+	TS92_00171	yidK	NA	putative symporter YidK	NA	1298	1556	1240	109	123	144	121	161	163	897.587482	1029.62114	803.40017	156.064595	139.699339	181.577	193.274888	164.037597	171.878136	415.237894	-2.5027659	0.17133928	-14.607076	2.5315E-48	3.3166E-47
TS92_Nanopo	.	CDS	607951	608502	-	TS92_00590	TS92_00590	NA	TetR family transcriptional regulator	NA	592	661	610	52	71	48	55	110	100	409.377342	437.390472	395.221051	74.4528341	80.6394561	60.5258998	87.852222	112.075375	105.446709	195.886818	-2.5065562	0.18637488	-13.449002	3.1206E-41	3.3625E-40
TS92_Nanopo	.	CDS	1709327	1709617	-	TS92_01775	TS92_01775	NA	hypothetical protein	NA	462	537	512	45	49	44	9	20	25	319.48029	355.338401	331.726522	64.4303372	55.6525824	55.4820748	14.3758181	20.3773412	26.3616773	138.136116	-2.5072861	0.20259751	-12.375701	3.5379E-35	3.0587E-34
TS92_Nanopo	.	CDS	1250554	1251423	+	TS92_01312	accD	NA	acetyl-coenzyme A carboxylase carboxyl%0Atransferase subunit beta	NA	6863	7208	6578	596	692	585	226	372	418	4745.8728	4769.60744	4261.90832	853.344022	785.950755	737.659404	360.992767	379.018547	440.767244	1926.12459	-2.5271152	0.12951516	-19.512119	8.6617E-85	2.8622E-83
TS92_Nanopo	.	CDS	552653	553840	+	TS92_00531	TS92_00531	NA	UTP-glucose-1-phosphate uridylyltransferase	NA	12327	11976	14005	1093	1228	1144	594	997	818	8524.31502	7924.64189	9073.8866	1564.9413	1394.72186	1442.53395	948.803997	1015.81046	862.55408	3639.13435	-2.5284005	0.12057903	-20.968824	1.2635E-97	5.0779E-96
TS92_Nanopo	.	CDS	770743	772113	+	TS92_00769	TS92_00769	NA	hypothetical protein	NA	4656	5050	4931	465	452	396	288	425	389	3219.69747	3341.63673	3194.81148	665.780151	513.366678	499.338674	460.026181	433.018501	410.187698	1415.31817	-2.531017	0.14187951	-17.8392	3.5068E-71	8.9138E-70
TS92_Nanopo	.	CDS	2289261	2289422	-	TS92_02389	TS92_02389	NA	hypothetical protein	NA	84	106	88	7	10	7	6	14	8	58.0873255	70.1412859	57.0154959	10.0224969	11.3576699	8.82669373	9.58387876	14.2641389	8.43573673	27.5260802	-2.5400577	0.3824295	-6.6418981	3.0967E-11	9.2932E-11
TS92_Nanopo	.	CDS	824330	825466	+	TS92_00825	mnaA	NA	UDP-N-acetylglucosamine 2-epimerase	NA	22974	20812	24090	1787	2218	2106	1078	1946	1990	15886.8835	13771.5136	15607.992	2558.60028	2519.13118	2655.57386	1721.90355	1982.7153	2098.38951	6533.63365	-2.5413745	0.12208441	-20.816537	3.0656E-96	1.2156E-94
TS92_Nanopo	.	CDS	655039	656058	+	TS92_00636	metN_1	NA	DL-methionine transporter ATP-binding subunit	NA	407	646	464	46	47	39	14	26	19	281.446922	427.464818	300.62716	65.8621225	53.3810484	49.1772936	22.3623838	26.4905436	20.0348747	138.538574	-2.5608753	0.24588804	-10.414802	2.1224E-25	1.2549E-24
TS92_Nanopo	.	CDS	601945	602754	-	TS92_00584	TS92_00584	NA	secretory antigen SsaA-like protein	NA	3696	3290	3082	227	417	250	113	204	174	2555.84232	2177.0267	1996.83816	325.015257	473.614834	315.239062	180.496383	207.848881	183.477274	935.04432	-2.5737301	0.1873236	-13.739486	5.8892E-43	6.6595E-42
TS92_Nanopo	.	CDS	1031340	1032392	+	TS92_01066	TS92_01066	NA	toxin regulatory protein	NA	482	857	459	44	58	52	15	43	53	333.310606	567.08568	297.387644	62.9985519	65.8744853	65.5697248	23.9596969	43.8112837	55.8867558	168.431603	-2.5763452	0.29660198	-8.6862038	3.7475E-18	1.6059E-17
TS92_Nanopo	.	CDS	502055	502411	-	TS92_00477	TS92_00477	NA	hypothetical protein	NA	140	209	184	13	17	14	12	66	27	96.8122092	138.297441	119.214219	18.6132085	19.3080388	17.6533875	19.1677575	67.2452261	28.4706115	58.3091221	-2.5853831	0.38805903	-6.6623451	2.6949E-11	8.1368E-11
TS92_Nanopo	.	CDS	136358	137203	+	TS92_00133	glcT_1	NA	transcriptional antiterminator	NA	805	820	801	81	71	56	87	155	119	556.670203	542.6024	518.970594	115.974607	80.6394561	70.6135498	138.966242	157.924395	125.481584	256.427003	-2.5877471	0.194789	-13.284873	2.8334E-40	2.9671E-39
TS92_Nanopo	.	CDS	1435860	1436252	+	TS92_01502	nusB	NA	putative N utilization substance protein B	NA	1708	1873	1970	133	190	158	145	346	258	1181.10895	1239.38329	1276.36962	190.427441	215.795728	199.231087	231.610403	352.528003	272.052509	573.167449	-2.5927973	0.17080539	-15.179833	4.81E-52	7.0123E-51
TS92_Nanopo	.	CDS	717474	718634	+	TS92_00697	TS92_00697	NA	N-succinyldiaminopimelate aminotransferase	NA	598	631	636	50	57	51	23	86	44	413.526436	417.539164	412.066539	71.5892636	64.7387183	64.3087686	36.7382019	87.6225673	46.396552	179.391801	-2.6012185	0.24871888	-10.458468	1.34E-25	8.0349E-25
TS92_Nanopo	.	CDS	1250080	1250535	+	TS92_01311	nanR	NA	HTH-type transcriptional repressor NanR	NA	3015	3326	2928	221	342	240	166	260	222	2084.92008	2200.84827	1897.06105	316.424545	388.43231	302.629499	265.159379	264.905436	234.091694	883.829651	-2.6036507	0.14736564	-17.667963	7.4022E-70	1.8194E-68
TS92_Nanopo	.	CDS	2119095	2119868	-	TS92_02195	fabI	NA	enoyl-acyl-carrier-protein reductase%2C FabI	NA	6390	6777	6915	540	629	560	352	548	496	4418.78583	4484.41033	4480.25175	773.164046	714.397435	706.135498	562.254221	558.33915	523.015677	1913.4171	-2.6041802	0.10486127	-24.834529	3.8E-136	2.756E-134
TS92_Nanopo	.	CDS	1720030	1720908	-	TS92_01788	rhaS_3	NA	HTH-type transcriptional activator RhaS	NA	165	195	164	12	22	10	19	33	22	114.100104	129.033498	106.256152	17.1814233	24.9868737	12.6095625	30.3489494	33.62613	23.198276	54.5930501	-2.6084761	0.30591651	-8.5267583	1.5051E-17	6.269E-17
TS92_Nanopo	.	CDS	1858215	1858694	-	TS92_01917	lspA	NA	lipoprotein signal peptidase	NA	1161	1176	1258	104	100	102	42	72	76	802.84982	778.171248	815.062431	148.905668	113.576699	128.617537	67.0871513	73.3584284	80.1394989	334.196498	-2.6093925	0.1589673	-16.41465	1.5025E-60	2.8103E-59
TS92_Nanopo	.	CDS	1126418	1126849	-	TS92_01189	yjaB	NA	Peptidyl-lysine N-acetyltransferase YjaB	NA	118	130	127	6	10	15	16	26	16	81.5988262	86.0223318	82.2837271	8.59071163	11.3576699	18.9143437	25.55701	26.4905436	16.8714735	39.7429637	-2.6109569	0.34294134	-7.6134212	2.6693E-14	9.496E-14
TS92_Nanopo	.	CDS	855727	855894	+	TS92_00862	mazE	NA	Antitoxin MazE	NA	2078	2487	2371	171	202	214	90	176	154	1436.96979	1645.67338	1536.17887	244.835281	229.424931	269.844457	143.758181	179.320603	162.387932	649.821512	-2.624155	0.14415858	-18.203252	4.8633E-74	1.303E-72
TS92_Nanopo	.	CDS	176459	177766	+	TS92_00170	moxC_1	NA	Putative monooxygenase MoxC	NA	1350	1715	1257	92	152	123	73	156	114	933.546303	1134.83307	814.414527	131.724245	172.636582	155.097618	116.603858	158.943262	120.209248	415.334301	-2.6250008	0.19690603	-13.331236	1.5235E-40	1.6182E-39
TS92_Nanopo	.	CDS	1465012	1465515	-	TS92_01533	TS92_01533	NA	hypothetical protein	NA	378	390	427	34	32	33	34	62	34	261.392965	258.066995	276.654736	48.6806992	36.3445436	41.6115661	54.3086463	63.1697578	35.8518811	119.564642	-2.6326341	0.23541096	-11.183141	4.9313E-29	3.3407E-28
TS92_Nanopo	.	CDS	363773	365236	-	TS92_00346	sasA_1	NA	Adaptive-response sensory-kinase SasA	NA	1136	1080	1248	86	95	108	44	84	80	785.561926	714.647064	808.583397	123.133533	107.897864	136.183275	70.2817776	85.5848332	84.3573673	324.025671	-2.6417357	0.1642255	-16.086026	3.1971E-58	5.5281E-57
TS92_Nanopo	.	CDS	622190	623134	+	TS92_00606	corA	NA	magnesium and cobalt transport protein	NA	2388	2248	2490	213	190	187	118	187	210	1651.33968	1487.52463	1613.27937	304.970263	215.795728	235.798818	188.482949	190.528141	221.438089	678.795297	-2.642064	0.1608733	-16.42326	1.3037E-60	2.4539E-59
TS92_Nanopo	.	CDS	343964	344449	+	TS92_00325	TS92_00325	NA	GNAT family acetyltransferase	NA	608	628	794	42	62	62	46	96	67	420.441594	415.554034	514.43527	60.1349814	70.4175532	78.1792873	73.4764038	97.8112379	70.6492951	200.122184	-2.6672295	0.20662306	-12.908673	4.0217E-38	3.9215E-37
TS92_Nanopo	.	CDS	2253667	2254410	-	TS92_02335	TS92_02335	NA	hypothetical protein	NA	190	251	223	16	18	18	7	35	16	131.387998	166.089271	144.48245	22.9085643	20.4438058	22.6972124	11.1811919	35.6603472	16.8714735	63.5247016	-2.6910386	0.32212891	-8.3539183	6.6035E-17	2.6396E-16
TS92_Nanopo	.	CDS	1487635	1488105	+	TS92_01557	TS92_01557	NA	heptaprenyl diphosphate synthase subunit 1	NA	964	1086	1023	73	110	63	48	102	76	666.621212	718.617326	662.80514	104.520325	124.934369	79.4402435	76.6710301	103.92444	80.1394989	290.85262	-2.7057065	0.19308534	-14.013008	1.2979E-44	1.5502E-43
TS92_Nanopo	.	CDS	2596272	2596475	-	TS92_02702	TS92_02702	NA	hypothetical protein	NA	17	36	22	3	2	0	0	0	0	11.7557683	23.8215688	14.253874	4.29535581	2.27153397	0	0	0	0	6.26645565	-2.7064334	0.74910678	-3.6128806	0.00030281	0.00054382
TS92_Nanopo	.	CDS	1248831	1250060	+	TS92_01310	maeB_2	NA	malate dehydrogenase%0A(oxaloacetate-decarboxylating) (NADP(+))	NA	10639	10648	9828	804	901	780	462	762	760	7357.03638	7045.89068	6367.59425	1151.15536	1023.32606	983.545872	737.958665	776.376701	801.394989	2916.031	-2.7100449	0.12083361	-22.427906	2.103E-111	1.025E-109
TS92_Nanopo	.	CDS	1553770	1554594	+	TS92_01623	menH_1	NA	2-succinyl-6-hydroxy-2%2C4-cyclohexadiene-1-carboxylate synthase	NA	366	367	506	25	32	40	19	34	36	253.094775	242.84766	327.839102	35.7946318	36.3445436	50.4382499	30.3489494	34.6414801	37.9608153	116.590023	-2.7176738	0.2427286	-11.196348	4.2489E-29	2.9049E-28
TS92_Nanopo	.	CDS	1578933	1579802	+	TS92_01648	glcT_2	NA	transcriptional antiterminator	NA	72	68	88	6	8	3	7	8	9	49.7891362	44.9962966	57.0154959	8.59071163	9.0861359	3.78286874	11.1811919	8.15093649	9.49020382	22.4536641	-2.7190425	0.43040323	-6.3174304	2.6595E-10	7.5398E-10
TS92_Nanopo	.	CDS	1629830	1630666	-	TS92_01699	glcT_3	NA	transcriptional antiterminator	NA	1860	2744	2088	164	205	147	136	419	201	1286.21935	1815.73291	1352.82222	234.812784	232.832232	185.360568	217.234585	426.905299	211.947885	662.651982	-2.7295606	0.26466303	-10.313343	6.133E-25	3.5834E-24
TS92_Nanopo	.	CDS	1773243	1773995	-	TS92_01835	uppS	NA	di-trans%2C poly-cis-decaprenylcistransferase	NA	2924	3183	3232	202	295	232	242	514	436	2021.99214	2106.22371	2094.02367	289.220625	335.051261	292.541849	386.549777	523.697657	459.747652	945.449817	-2.7485394	0.14707553	-18.687945	6.2058E-78	1.8456E-76
TS92_Nanopo	.	CDS	146705	147643	+	TS92_00140	TS92_00140	NA	hypothetical protein	NA	81	87	79	5	10	3	9	18	6	56.0127782	57.5687913	51.1843657	7.15892636	11.3576699	3.78286874	14.3758181	18.3396071	6.32680255	25.1230698	-2.7488513	0.44118029	-6.2306757	4.6443E-10	1.2957E-09
TS92_Nanopo	.	CDS	2410864	2411559	-	TS92_02515	TS92_02515	NA	ABC transporter ATP-binding protein	NA	557	813	590	60	44	43	26	42	33	385.174289	537.970429	382.262984	85.9071163	49.9737474	54.2211186	41.5301413	42.7924166	34.797414	179.403295	-2.7594636	0.24623458	-11.206645	3.7826E-29	2.598E-28
TS92_Nanopo	.	CDS	1431112	1431702	-	TS92_01496	TS92_01496	NA	hypothetical protein	NA	566	594	746	51	48	45	33	63	45	391.397931	393.055885	483.335909	73.0210488	54.5168154	56.7430311	52.7113332	64.1886249	47.4510191	179.6024	-2.7674395	0.21088882	-13.122742	2.4395E-39	2.4846E-38
TS92_Nanopo	.	CDS	998575	998892	+	TS92_01013	yfhH	NA	putative protein YfhH	NA	514	522	568	28	48	46	36	48	51	355.439111	345.412748	368.00911	40.0899876	54.5168154	58.0039873	57.5032726	48.905619	53.7778216	153.517608	-2.7773016	0.20090947	-13.823647	1.8353E-43	2.1156E-42
TS92_Nanopo	.	CDS	1488863	1489825	+	TS92_01559	gerCC	NA	iron (Fe3+) ABC superfamily ATP binding%0Acassette transporter%2C membrane protein	NA	2460	2472	2702	195	198	184	112	167	184	1701.12882	1635.74772	1750.63489	279.198128	224.881864	232.015949	178.89907	170.150799	194.021945	707.408798	-2.7824173	0.1392728	-19.978182	8.5272E-89	2.9835E-87
TS92_Nanopo	.	CDS	2469968	2470672	+	TS92_02577	TS92_02577	NA	hypothetical protein	NA	9	16	14	1	1	0	0	0	0	6.22364202	10.5873639	9.07064708	1.43178527	1.13576699	0	0	0	0	3.16102281	-2.793665	0.90618987	-3.0828694	0.00205015	0.00334457
TS92_Nanopo	.	CDS	1995597	1995809	+	TS92_02081	TS92_02081	NA	hypothetical protein	NA	1975	1762	2249	118	197	134	162	346	258	1365.74367	1165.93345	1457.13466	168.950662	223.746097	168.968137	258.764727	352.528003	272.052509	603.75799	-2.8042372	0.18606058	-15.071635	2.4888E-51	3.5585E-50
TS92_Nanopo	.	CDS	560691	561752	+	TS92_00539	TS92_00539	NA	chromosome partitioning ATPase	NA	9162	9451	10088	626	800	708	864	1533	1928	6335.66758	6253.82352	6536.04913	896.29758	908.61359	892.757023	1380.07854	1561.92321	2033.01255	2977.5803	-2.8114938	0.15442823	-18.205828	4.6398E-74	1.2544E-72
TS92_Nanopo	.	CDS	1707535	1709259	-	TS92_01774	msbA_8	NA	Lipid A export ATP-binding/permease MsbA	NA	2078	2119	2167	125	171	176	64	98	94	1436.96979	1402.16401	1404.00659	178.973159	194.216155	221.928299	102.22804	99.848972	99.1199066	571.050546	-2.8218566	0.1387052	-20.344274	5.2179E-92	1.8924E-90
TS92_Nanopo	.	CDS	1503330	1504541	+	TS92_01575	papS	NA	polynucleotide adenylyltransferase	NA	2372	2193	2364	165	167	171	115	274	238	1640.27543	1451.13057	1531.64355	236.24457	189.673087	215.623518	183.69101	279.169575	250.963168	664.268275	-2.8346665	0.17328293	-16.358602	3.777E-60	6.9769E-59
TS92_Nanopo	.	CDS	351414	352391	-	TS92_00333	add	NA	Adenosine deaminase	NA	611	682	681	43	57	44	52	94	80	422.516142	451.286387	441.22219	61.5667667	64.7387183	55.4820748	83.0602826	95.7735038	84.3573673	195.555937	-2.8346978	0.18297239	-15.49249	3.8989E-54	5.9464E-53
TS92_Nanopo	.	rRNA	40069	40548	+	TS92_00038	TS92_00038	NA		NA	118	130	122	8	10	8	12	14	18	81.5988262	86.0223318	79.0442103	11.4542822	11.3576699	10.08765	19.1677575	14.2641389	18.9804076	36.8863678	-2.8369497	0.34955097	-8.1159827	4.8187E-16	1.8515E-15
TS92_Nanopo	.	rRNA	40069	40548	+		orfX	NA		NA	118	130	122	8	10	8	12	14	18	81.5988262	86.0223318	79.0442103	11.4542822	11.3576699	10.08765	19.1677575	14.2641389	18.9804076	36.8863678	-2.8369497	0.34955097	-8.1159827	4.8187E-16	1.8515E-15
TS92_Nanopo	.	CDS	120955	122022	+	TS92_00114	TS92_00114	NA	major facilitator superfamily multidrug :cation%0Asymporter	NA	1340	1375	1682	92	118	110	300	380	453	926.631145	909.851586	1089.77346	131.724245	134.020505	138.705187	479.193938	387.169483	477.673592	519.415904	-2.8381159	0.16425766	-17.278438	6.8374E-67	1.5175E-65
TS92_Nanopo	.	CDS	1495701	1496027	+	TS92_01566	TS92_01566	NA	hypothetical protein	NA	148	165	186	9	16	10	9	17	13	102.344335	109.18219	120.510026	12.8860674	18.1722718	12.6095625	14.3758181	17.32074	13.7080722	46.7898981	-2.8593207	0.31810836	-8.9885114	2.506E-19	1.1344E-18
TS92_Nanopo	.	CDS	1347692	1348333	+	TS92_01398	TS92_01398	NA	hypothetical protein	NA	351	406	399	29	35	16	23	82	35	242.722039	268.654359	258.513444	41.5217729	39.7518446	20.1752999	36.7382019	83.5470991	36.9063482	114.281156	-2.8624652	0.32391668	-8.8370417	9.8276E-19	4.324E-18
TS92_Nanopo	.	CDS	2420213	2420770	+	TS92_02525	TS92_02525	NA	hypothetical protein	NA	292	325	317	14	25	27	26	46	42	201.922608	215.055829	205.385366	20.0449938	28.3941747	34.0458187	41.5301413	46.8678848	44.2876178	93.0593816	-2.8693087	0.24428717	-11.745638	7.4359E-32	5.7291E-31
TS92_Nanopo	.	CDS	2812931	2813506	-	TS92_02935	xprT	NA	putative xanthine phosphoribosyltransferase	NA	3528	3968	3159	242	286	227	135	240	167	2439.66767	2625.66625	2046.72672	346.492036	324.829358	286.237068	215.637272	244.528095	176.096004	967.320053	-2.876848	0.16915723	-17.006947	7.2941E-65	1.4858E-63
TS92_Nanopo	.	CDS	2824688	2825485	+	TS92_02945	TS92_02945	NA	hypothetical protein	NA	380	380	360	23	34	22	18	38	33	262.775996	251.449893	233.245211	32.9310612	38.6160776	27.7410374	28.7516363	38.7169483	34.797414	105.447253	-2.8771796	0.23364132	-12.314515	7.5669E-35	6.4666E-34
TS92_Nanopo	.	CDS	885450	886229	+	TS92_00894	tatC	NA	Sec-independent periplasmic protein translocase	NA	2272	2257	2286	127	207	154	84	130	119	1571.12385	1493.48002	1481.10709	181.836729	235.103766	194.187262	134.174303	132.452718	125.481584	616.549703	-2.8784835	0.14431808	-19.945411	1.6429E-88	5.6815E-87

TS92_Nanopo	.	CDS	2021294	2022421	-	TS92_02107	purK	NA	phosphoribosylaminoimidazole carboxylase ATPase%0Asubunit	NA	2261	3340	2544	166	238	169	30	51	45	1563.51718	2210.11222	1648.26616	237.676355	270.312543	213.101606	47.9193938	51.9622201	47.4510191	698.924299	-2.890024	0.18514681	-15.609364	6.286E-55	9.9971E-54
TS92_Nanopo	.	CDS	1833942	1834832	-	TS92_01895	TS92_01895	NA	hypothetical protein	NA	304	256	342	19	23	20	23	47	44	210.220797	169.397823	221.58295	27.2039202	26.1226407	25.2191249	36.7382019	47.8867519	46.396552	90.0854179	-2.8983945	0.25962997	-11.163559	6.148E-29	4.1461E-28
TS92_Nanopo	.	CDS	1554873	1555688	+	TS92_01624	TS92_01624	NA	Glyoxalase/bleomycin resistance%0Aprotein/dioxygenase	NA	6230	5894	6904	406	382	498	363	1016	854	4308.14331	3900.12018	4473.12482	581.30482	433.862989	627.956211	579.824665	1035.16893	900.514896	1871.11342	-2.9198569	0.2168734	-13.463417	2.5676E-41	2.7971E-40
TS92_Nanopo	.	CDS	686090	686836	+	TS92_00669	TS92_00669	NA	acetyltransferase (GNAT) family protein	NA	5852	6608	6554	436	310	516	172	244	275	4046.75034	4372.5813	4246.35864	624.258378	352.087766	650.653423	274.737858	248.603563	289.97845	1678.44552	-2.9354777	0.20976459	-13.994153	1.6923E-44	2.0052E-43
TS92_Nanopo	.	CDS	1218377	1219492	+	TS92_01280	TS92_01280	NA	hypothetical protein	NA	3881	3907	3974	245	290	256	100	277	193	2683.77274	2585.30193	2574.76796	350.787391	329.372426	322.804799	159.731313	282.226176	203.512149	1054.69743	-2.9480951	0.18072573	-16.312537	8.0384E-60	1.4666E-58
TS92_Nanopo	.	CDS	2253119	2253655	-	TS92_02334	TS92_02334	NA	hypothetical protein	NA	318	408	390	24	34	15	14	48	26	219.902018	269.97778	252.682312	34.3628465	38.6160776	18.9143437	22.3623838	48.905619	27.4161444	103.682169	-2.9605487	0.29515916	-10.030347	1.1212E-23	6.1072E-23
TS92_Nanopo	.	CDS	769511	770527	+	TS92_00768	TS92_00768	NA	N-acetylmuramoyl-L-alanine amidase	NA	12910	11298	11040	735	908	708	322	521	329	8927.46872	7476.00234	7152.85313	1052.36217	1031.27642	892.757023	514.334827	530.829739	346.919673	3102.75601	-2.9643582	0.18048669	-16.424248	1.2826E-60	2.4452E-59
TS92_Nanopo	.	CDS	2203114	2204574	-	TS92_02274	dltA	NA	D-alanine-activating enzyme/D-alanine-D-alanyl%2C%0AdltA protein	NA	15564	17017	17542	1036	1178	1118	909	1932	1576	10762.7516	11260.3232	11365.5208	1483.32954	1337.93351	1409.74908	1451.95763	1968.45116	1661.84014	4744.65074	-2.969012	0.13667722	-21.7228	1.249E-104	5.612E-103
TS92_Nanopo	.	CDS	1579795	1580127	+	TS92_01649	TS92_01649	NA	PTS system lactose/cellobiose-specific IIA%0Acomponent	NA	70	82	62	3	5	5	4	8	6	48.4061046	54.26024	40.1700085	4.29535581	5.67883494	6.30478123	6.38925251	8.15093649	6.32680255	19.9980352	-2.9893721	0.46685353	-6.4032333	1.5212E-10	4.3753E-10
TS92_Nanopo	.	CDS	855895	856266	+	TS92_00863	mazF	NA	Toxin MazF	NA	3698	4448	4109	250	274	266	138	281	236	2557.22535	2943.28717	2662.23492	357.946318	311.200155	335.414362	220.429212	286.301644	248.854233	1102.54371	-3.0119244	0.1428535	-21.084008	1.1153E-98	4.6067E-97
TS92_Nanopo	.	CDS	2522570	2523250	-	TS92_02630	rpiA	NA	ribose 5-phosphate isomerase	NA	251	222	222	20	12	11	11	20	14	173.570461	146.899674	143.834547	28.6357054	13.6292038	13.8705187	17.5704444	20.3773412	14.7625393	63.6833816	-3.0250636	0.30717267	-9.8480884	6.986E-23	3.7035E-22
TS92_Nanopo	.	CDS	2604545	2605120	+	TS92_02714	TS92_02714	NA	hypothetical protein	NA	318	251	338	13	29	15	19	52	43	219.902018	166.089271	218.991337	18.6132085	32.9372426	18.9143437	30.3489494	52.9810872	45.3420849	89.3466158	-3.0282229	0.29443822	-10.284748	8.2558E-25	4.7768E-24
TS92_Nanopo	.	CDS	1343197	1343838	+	TS92_01393	TS92_01393	NA	caffeoyl-CoA O-methyltransferase	NA	2473	2480	2356	162	176	112	168	267	232	1710.11852	1641.04141	1526.46032	231.949214	199.89499	141.2271	268.348605	272.037505	244.636365	692.857115	-3.0725645	0.17623669	-17.434307	4.5302E-68	1.0526E-66
TS92_Nanopo	.	CDS	2427408	2427926	-	TS92_02533	TS92_02533	NA	acetyltransferase	NA	760	1030	807	39	60	61	25	56	43	525.551993	681.561552	522.858014	55.8396256	68.1460192	76.918331	39.9328282	57.0565555	45.3420849	230.356334	-3.0746375	0.21390974	-14.373528	7.5866E-47	9.6011E-46
TS92_Nanopo	.	CDS	2294299	2295642	-	TS92_02396	aroA	NA	3-phosphoshikimate 1-carboxyvinyltransferase	NA	2201	2414	2146	133	170	116	86	145	124	1522.02623	1597.36853	1390.40062	190.427441	193.080388	146.270925	137.368929	147.735724	130.753919	606.159189	-3.0748369	0.1568425	-19.604616	1.4122E-85	4.7724E-84
TS92_Nanopo	.	CDS	1326992	1327726	+	TS92_01380	TS92_01380	NA	glutamyl endopeptidase	NA	1631	838	1240	76	84	61	33	33	19	1127.86224	554.513185	803.40017	108.815681	95.4044269	76.918331	52.7113332	33.622613	20.0348747	319.25365	-3.0893394	0.30014237	-10.292913	7.5846E-25	4.4056E-24
TS92_Nanopo	.	CDS	215629	216792	+	TS92_00209	xylR_2	NA	xylose operon repressor	NA	250	376	310	25	18	11	10	22	10	172.878945	248.803052	200.850043	35.7946318	20.4438058	13.8705187	15.9731313	22.4150754	10.5446709	82.397097	-3.1092377	0.32362031	-9.6076717	7.4208E-22	3.7985E-21
TS92_Nanopo	.	CDS	998905	1000452	+	TS92_01014	TS92_01014	NA	ABC-type polysaccharide/polyol phosphate%0Atransport system ATPase component-like r	NA	3640	3688	4020	211	216	240	118	253	257	2517.11744	2440.38738	2604.57152	302.106692	245.325669	302.629499	188.482949	257.773367	270.998042	1014.37695	-3.1389262	0.16087631	-19.511426	8.7798E-85	2.8694E-83
TS92_Nanopo	.	CDS	1443624	1443881	-	TS92_01510	TS92_01510	NA	hypothetical protein	NA	683	775	660	44	41	38	38	61	39	472.305278	512.825439	427.61622	62.9985519	46.5664465	47.9163374	60.6978988	62.1508908	41.1242166	192.689031	-3.1463592	0.21634822	-14.543032	6.4664E-48	8.3613E-47
TS92_Nanopo	.	CDS	774358	775782	+	TS92_00772	TS92_00772	NA	putative Na+/H+ antiporter	NA	6010	6955	6221	360	359	369	169	408	327	4156.00984	4602.19475	4030.60682	515.442698	407.740349	465.292855	269.945918	415.697761	344.810739	1689.74908	-3.1854678	0.171527	-18.571233	5.4927E-77	1.586E-75
TS92_Nanopo	.	CDS	1807594	1808163	-	TS92_01869	fapR	NA	fatty acid biosynthesis transcriptional%0Aregulator	NA	2268	2538	2349	106	135	162	60	134	107	1568.35779	1679.4206	1521.925	151.769239	153.328543	204.274912	95.8387876	136.528186	112.827979	624.919004	-3.2077118	0.17256753	-18.588154	4.0074E-77	1.1684E-75
TS92_Nanopo	.	CDS	2245888	2246388	-	TS92_02325	speG	NA	Spermidine N(1)-acetyltransferase	NA	330	454	436	29	19	17	5	7	7	228.200207	300.416451	282.485866	41.5217729	21.5795728	21.4362562	7.98656563	7.13206943	7.38126964	102.015559	-3.2443373	0.28077944	-11.554754	6.9847E-31	5.1417E-30
TS92_Nanopo	.	CDS	2754488	2755216	-	TS92_02876	TS92_02876	NA	O-methyltransferase	NA	354	330	356	19	19	18	17	32	40	244.796586	218.364381	230.653597	27.2039202	21.5795728	22.6972124	27.1543232	32.603746	42.1786836	96.3591136	-3.2462938	0.25833599	-12.56617	3.2405E-36	2.9382E-35
TS92_Nanopo	.	CDS	1488146	1488850	+	TS92_01558	TS92_01558	NA	putative 2-heptaprenyl-1%2C4-naphthoquinone%0Amethyltransferase	NA	1185	1194	1336	62	54	78	40	87	54	819.446199	790.082032	865.598893	88.7706868	61.3314173	98.3545872	63.8925251	88.6414344	56.9412229	325.895444	-3.2949282	0.2093371	-15.73982	8.0673E-56	1.3039E-54
TS92_Nanopo	.	CDS	1566413	1566715	+	TS92_01636	TS92_01636	NA	hypothetical protein	NA	1292	1657	1862	70	130	52	156	265	269	893.438388	1096.45388	1206.39606	100.224969	147.649708	65.5697248	249.180848	269.999771	283.651647	479.173888	-3.3034031	0.24153678	-13.676605	1.4008E-42	1.5662E-41
TS92_Nanopo	.	CDS	856363	857367	+	TS92_00864	rsbU	NA	putative sigma factor sigB regulation protein	NA	5616	5802	5796	279	357	272	232	389	310	3883.55262	3839.24284	3755.24789	399.468091	405.468815	342.980099	370.576645	396.339287	326.884798	1524.4179	-3.3103559	0.1304237	-25.381553	4.031E-142	3.155E-140
TS92_Nanopo	.	CDS	2822825	2823646	+	TS92_02943	TS92_02943	NA	hypothetical protein	NA	525	498	558	29	27	25	8	32	34	363.045784	329.531702	361.530077	41.5217729	30.6657087	31.5239062	12.778505	32.603746	35.8518811	137.672565	-3.3171715	0.25034188	-13.250565	4.4782E-40	4.673E-39
TS92_Nanopo	.	CDS	2034973	2035734	-	TS92_02120	menH_2	NA	2-succinyl-6-hydroxy-2%2C%0A4-cyclohexadiene-1-carboxylate synthase	NA	1150	1078	1222	60	68	48	108	223	200	795.243147	713.323644	791.73791	85.9071163	77.2321551	60.5258998	172.509818	227.207355	210.893418	348.286718	-3.341735	0.18620622	-17.94642	5.1185E-72	1.3123E-70
TS92_Nanopo	.	CDS	2022408	2022890	-	TS92_02108	purE	NA	putative phosphoribosylaminoimidazole%0Acarboxylase catalytic subunit	NA	858	1314	973	38	68	48	12	30	10	593.320539	869.487261	630.409972	54.4078403	77.2321551	60.5258998	19.1677575	30.5660119	10.5446709	260.629123	-3.3949379	0.26189437	-12.963005	1.9832E-38	1.953E-37
TS92_Nanopo	.	CDS	1588639	1589217	-	TS92_01659	TS92_01659	NA	hypothetical protein	NA	385	432	393	16	29	14	25	49	34	266.233575	285.858826	254.626022	22.9085643	32.9372426	17.6533875	39.9328282	49.924486	35.8518811	111.769646	-3.3961461	0.25983027	-13.070633	4.846E-39	4.869E-38
TS92_Nanopo	.	CDS	2519553	2520206	-	TS92_02627	TS92_02627	NA	hypothetical protein	NA	281	291	318	11	18	14	13	31	26	194.315934	192.557681	206.033269	15.749638	20.4438058	17.6533875	20.7650706	31.5848789	27.4161444	80.7244233	-3.3991802	0.27906142	-12.18076	3.936E-34	3.2789E-33
TS92_Nanopo	.	CDS	1242808	1244103	+	TS92_01306	TS92_01306	NA	transcriptional regulator	NA	1584	1628	1748	84	93	62	75	212	152	1095.361	1077.26428	1132.53508	120.269963	105.62633	78.1792873	119.798485	215.999817	160.278998	456.145915	-3.4108752	0.21980139	-15.517987	2.6215E-54	4.0605E-53
TS92_Nanopo	.	CDS	937438	938496	-	TS92_00950	TS92_00950	NA	nitric-oxide synthase%2C oxygenase subunit	NA	932	989	916	40	56	42	39	75	48	644.492707	654.431432	593.47948	57.2714108	63.6029513	52.9601624	62.2952119	76.4150296	50.6144204	250.61809	-3.415948	0.1972891	-17.314429	3.6614E-67	8.1872E-66
TS92_Nanopo	.	CDS	953276	953959	+	TS92_00968	pcrB	NA	putative glycerol-1-phosphate prenyltransferase	NA	1620	1439	1592	60	70	85	85	212	159	1120.25556	952.201042	1031.46215	85.9071163	79.5036891	107.181281	135.771616	215.999817	167.660267	432.882505	-3.4807524	0.20250819	-17.188205	3.2545E-66	6.9633E-65
TS92_Nanopo	.	CDS	1348659	1349384	+	TS92_01399	TS92_01399	NA	allophanate hydrolase subunit 1	NA	3848	3578	3913	154	182	188	42	104	72	2660.95272	2367.59925	2535.24586	220.494932	206.709592	237.059774	67.0871513	105.962174	75.9216306	941.892565	-3.4927546	0.16005682	-21.821966	1.436E-105	6.568E-104
TS92_Nanopo	.	CDS	318718	320538	+	TS92_00303	cysJ	NA	sulfite reductase (NADPH) flavoprotein%0Aalpha-component	NA	2294	2308	2132	119	97	88	30	36	26	1586.3372	1527.22724	1381.32997	170.382447	110.169398	110.96415	47.9193938	36.6792142	27.4161444	555.380573	-3.5008521	0.2036563	-17.190001	3.1552E-66	6.7998E-65
TS92_Nanopo	.	CDS	517346	517675	+	TS92_00491	sarV	NA	staphylococcal accessory regulator family%0Aprotein	NA	931	1055	1044	36	69	30	58	88	42	643.801191	698.104308	676.411111	51.5442698	78.3679221	37.8286874	92.6441614	89.6603014	44.2876178	268.072174	-3.5237183	0.28006873	-12.58162	2.6651E-36	2.4238E-35
TS92_Nanopo	.	CDS	1504528	1505496	+	TS92_01576	birA	NA	bifunctional biotin-(acetyl-CoA carboxylase)%0Aligase / biotin operon repressor	NA	1699	1558	1875	59	90	81	35	120	100	1174.88531	1030.94456	1214.81881	84.475331	102.219029	102.137456	55.9059594	122.264047	105.446709	443.677468	-3.5265299	0.22528568	-15.653591	3.1399E-55	5.0205E-54
TS92_Nanopo	.	CDS	1502207	1503328	+	TS92_01574	TS92_01574	NA	lipopolysaccharide biosynthesis-related pr-like%0Aprotein	NA	1583	1554	1734	74	82	60	88	181	170	1094.66948	1028.29772	1123.46443	105.95211	93.132893	75.6573748	140.563555	184.414938	179.259405	447.26799	-3.5445255	0.17613309	-20.124132	4.5365E-90	1.6061E-88
TS92_Nanopo	.	CDS	359377	360300	-	TS92_00341	fhuD_1	NA	iron (Fe+3) ABC superfamily ATP binding%0Acassette transporter%2C binding protein	NA	1684	2267	1791	68	106	79	21	53	45	1164.51257	1500.09712	1160.39492	97.3613984	120.391301	99.6155435	33.5435757	53.9999543	47.4510191	475.263046	-3.5613679	0.19556991	-18.210204	4.2835E-74	1.1687E-72
TS92_Nanopo	.	CDS	997779	998582	+	TS92_01012	recX	NA	recombination regulator RecX	NA	1636	1772	1982	60	80	92	63	141	105	1131.31982	1172.55055	1284.14447	85.9071163	90.861359	116.007975	100.630727	143.660256	110.719045	470.64459	-3.5894051	0.18476291	-19.427087	4.5549E-84	1.4411E-82
TS92_Nanopo	.	CDS	2346647	2347612	-	TS92_02451	sstB	NA	iron (Fe+3) ABC superfamily ATP binding%0Acassette transporter%2C membrane protein	NA	604	770	562	25	24	32	4	9	12	417.675531	509.516888	364.12169	35.7946318	27.2584077	40.3505999	6.38925251	9.16980356	12.6536051	158.103379	-3.6115196	0.25404934	-14.215819	7.3091E-46	9.0196E-45
TS92_Nanopo	.	CDS	2081290	2082447	+	TS92_02155	TS92_02155	NA	aromatic amino acid transferase	NA	1585	1524	1787	75	57	70	14	26	38	1096.05251	1008.44641	1157.80331	107.383895	64.7387183	88.2669373	22.3623838	26.4905436	40.0697495	401.290496	-3.6283157	0.20845314	-17.405906	7.442E-68	1.7157E-66
TS92_Nanopo	.	CDS	2634512	2634724	+	TS92_02744	TS92_02744	NA	hypothetical protein	NA	132	136	172	9	3	5	2	5	7	91.2800829	89.9925933	111.439378	12.8860674	3.40730096	6.30478123	3.19462625	5.09433531	7.38126964	36.7756039	-3.6389985	0.41671368	-8.7326111	2.4886E-18	1.0789E-17
TS92_Nanopo	.	CDS	2035788	2036069	-	TS92_02121	TS92_02121	NA	hypothetical protein	NA	1000	944	1042	33	44	46	85	206	217	691.51578	624.654471	675.115304	47.2489139	49.9737474	58.0039873	135.771616	209.886615	228.819359	302.332199	-3.6400148	0.22363086	-16.27689	1.44E-59	2.5956E-58
TS92_Nanopo	.	CDS	1884575	1884811	+	TS92_01944	TS92_01944	NA	hypothetical protein	NA	58	68	73	3	3	1	1	7	0	40.1079152	44.9962966	47.2969455	4.29535581	3.40730096	1.26095625	1.59731313	7.13206943	0	16.6771281	-3.6401106	0.58431203	-6.2297376	4.6722E-10	1.3023E-09
TS92_Nanopo	.	CDS	2209400	2210731	-	TS92_02282	TS92_02282	NA	bifunctional 5-nucleotidase/2%2C3-cyclic%0Aphosphodiesterase	NA	5736	5220	6174	208	223	274	240	470	414	3966.53451	3454.12748	4000.15536	297.811336	253.276038	345.502012	383.35515	478.867519	436.549376	1512.90875	-3.6535298	0.15870941	-23.020248	2.923E-117	1.738E-115
TS92_Nanopo	.	CDS	2732077	2733357	-	TS92_02852	tilS	NA	tRNA(Ile)-lysidine synthetase	NA	1856	1869	2118	67	96	72	92	242	130	1283.45329	1236.73645	1372.25932	95.9296132	109.033631	90.7888498	146.952808	246.565829	137.080722	524.311168	-3.6779694	0.2233426	-16.467836	6.2465E-61	1.2063E-59
TS92_Nanopo	.	CDS	1952710	1953099	+	TS92_02033	TS92_02033	NA	hypothetical protein	NA	182	218	205	11	5	7	10	24	23	125.855872	144.252833	132.820189	15.749638	5.67883494	8.82669373	15.9731313	24.4528095	24.2527431	55.3180828	-3.6826312	0.35560229	-10.356039	3.929E-25	2.3138E-24
TS92_Nanopo	.	CDS	1437587	1437802	+	TS92_01504	xseB	NA	putative exodeoxyribonuclease VII small subunit	NA	732	750	840	26	42	22	19	50	46	506.189551	496.282683	544.238825	37.226417	47.7022135	27.7410374	30.3489494	50.9433531	48.5054862	198.797613	-3.7326139	0.23123047	-16.142397	1.2845E-58	2.2341E-57
TS92_Nanopo	.	CDS	365241	365900	-	TS92_00347	walR_2	NA	transcriptional regulator WalR	NA	1365	1324	1518	43	60	60	34	91	64	943.91904	876.104364	983.517305	61.5667667	68.1460192	75.6573748	54.3086463	92.7169026	67.4858938	358.158035	-3.7396713	0.19834684	-18.854201	2.7141E-79	8.3213E-78
TS92_Nanopo	.	CDS	2093503	2093718	+	TS92_02167	TS92_02167	NA	hypothetical protein	NA	226	250	210	10	7	7	13	72	30	156.282566	165.427561	136.059706	14.3178527	7.95036891	8.82669373	20.7650706	73.3584284	31.6340127	68.2913623	-3.7419772	0.42401334	-8.8251403	1.0932E-18	4.7954E-18
TS92_Nanopo	.	CDS	23137	23463	+	TS92_00021	azlD	NA	Branched-chain amino acid transport protein%2C%0AAzlD	NA	104	128	100	2	8	2	1	2	2	71.9176411	84.6989113	64.7903363	2.86357054	9.0861359	2.52191249	1.59731313	2.03773412	2.10893418	26.8469432	-3.7438535	0.47794828	-7.833177	4.7569E-15	1.738E-14
TS92_Nanopo	.	CDS	1681748	1682194	+	TS92_01751	mepB	NA	MATE family multidrug efflux pump protein	NA	326	333	344	8	18	11	9	28	18	225.434144	220.349511	222.878757	11.4542822	20.4438058	13.8705187	14.3758181	28.5282777	18.9804076	86.2572803	-3.7802437	0.30031381	-12.587645	2.4693E-36	2.2527E-35
TS92_Nanopo	.	CDS	1436265	1437590	+	TS92_01503	xseA	NA	putative exodeoxyribonuclease VII large subunit	NA	3365	3316	3524	112	130	130	160	320	268	2326.9506	2194.23117	2283.21145	160.35995	147.649708	163.924312	255.5701	326.03746	282.59718	904.503548	-3.8350794	0.1456561	-26.329687	8.77E-153	8.694E-151
TS92_Nanopo	.	CDS	923363	924079	-	TS92_00933	agrA	NA	autoinducer sensor protein response regulator%0Aprotein	NA	4234	3618	3710	130	164	123	51	116	72	2927.87781	2394.06766	2403.72148	186.132085	186.265786	155.097618	81.4629695	118.188579	75.9216306	947.637291	-3.8464571	0.1851318	-20.776858	7.0098E-96	2.7074E-94
TS92_Nanopo	.	CDS	2411571	2412398	-	TS92_02516	TS92_02516	NA	amino acid ABC superfamily ATP binding cassette%0Atransporter%2C binding protein	NA	543	796	514	22	22	20	11	25	23	375.493068	526.721355	333.022329	31.499276	24.9868737	25.2191249	17.5704444	25.4716765	24.2527431	153.804099	-3.869984	0.27862798	-13.889431	7.3417E-44	8.5624E-43
TS92_Nanopo	.	CDS	2495195	2496100	+	TS92_02603	cysM	NA	Cystathionine beta-synthase%2C CysM	NA	9570	12016	10310	352	358	384	196	599	340	6617.80601	7951.1103	6679.88367	503.988415	406.604582	484.207199	313.073373	610.30137	358.518811	2658.38819	-3.8871576	0.2283939	-17.019533	5.8838E-65	1.2068E-63
TS92_Nanopo	.	CDS	1501873	1502187	+	TS92_01573	mazG_1	NA	nucleoside triphosphate pyrophosphohydrolase	NA	298	402	401	7	15	14	22	50	36	206.071702	266.007518	259.809249	10.0224969	17.0365048	17.6533875	35.1408888	50.9433531	37.9608153	100.071768	-3.9360483	0.30527494	-12.893454	4.8999E-38	4.7467E-37
TS92_Nanopo	.	CDS	1023440	1023724	+	TS92_01059	TS92_01059	NA	hypothetical protein	NA	409	461	490	14	18	13	2	18	14	282.829954	305.048423	317.472648	20.0449938	20.4438058	16.3924312	3.19462625	18.3396071	14.7625393	110.94767	-3.9381469	0.28350516	-13.890918	7.1908E-44	8.4194E-43
TS92_Nanopo	.	CDS	2823762	2824664	+	TS92_02944	coaA	NA	Pantothenate kinase	NA	963	1131	1132	26	42	40	31	30	31	665.929696	748.394287	733.426607	37.226417	47.7022135	50.4382499	49.5167069	30.5660119	32.6884798	266.209852	-3.9483247	0.21031976	-18.772961	1.2568E-78	3.7755E-77
TS92_Nanopo	.	CDS	2150248	2150451	+	TS92_02223	TS92_02223	NA	hypothetical protein	NA	674	839	688	18	32	23	16	40	33	466.081636	555.174895	445.757514	25.7721349	36.3445436	29.0019937	25.55701	40.7546825	34.797414	184.360203	-3.9534964	0.24202445	-16.335112	5.5531E-60	1.0194E-58
TS92_Nanopo	.	CDS	887792	888745	+	TS92_00897	scrR	NA	sucrose operon repressor	NA	719	709	713	27	17	23	25	57	35	497.199846	469.152563	461.955098	38.6582023	19.3080388	29.0019937	39.9328282	58.0754225	36.9063482	183.354482	-4.011532	0.25223245	-15.904108	5.9343E-57	9.8047E-56
TS92_Nanopo	.	CDS	1437795	1438673	+	TS92_01505	ispA	NA	putative geranyltranstransferase	NA	3875	3708	4158	102	138	134	96	160	180	2679.62365	2453.62159	2693.98218	146.042098	156.735844	168.968137	153.34206	163.01873	189.804076	978.348707	-4.0338401	0.146114	-27.607486	9.047E-168	9.965E-166
TS92_Nanopo	.	CDS	988067	988270	+	TS92_01001	TS92_01001	NA	hypothetical protein	NA	738	960	946	16	34	22	12	43	34	510.338646	635.241835	612.916581	22.9085643	38.6160776	27.7410374	19.1677575	43.8112837	35.8518811	216.288185	-4.2281458	0.26159041	-16.163229	9.1633E-59	1.603E-57
TS92_Nanopo	.	CDS	2534063	2534893	-	TS92_02639	glcT_4	NA	transcriptional antiterminator	NA	609	648	679	16	20	16	10	25	27	421.13311	428.788238	439.926383	22.9085643	22.7153397	20.1752999	15.9731313	25.4716765	28.4706115	158.395817	-4.2461405	0.25086781	-16.925809	2.9033E-64	5.8738E-63
TS92_Nanopo	.	CDS	1408780	1409334	+	TS92_01466	TS92_01466	NA	5-formyltetrahydrofolate cyclo-ligase	NA	782	886	1016	14	33	20	31	74	36	540.76534	586.275277	658.269817	20.0449938	37.4803106	25.2191249	49.5167069	75.3961626	37.9608153	225.658727	-4.3437203	0.27855615	-15.593697	8.0345E-55	1.271E-53
TS92_Nanopo	.	CDS	633911	634546	-	TS92_00619	TS92_00619	NA	hypothetical protein	NA	249	269	268	6	6	6	10	12	12	172.187429	178.000056	173.638101	8.59071163	6.81460192	7.56573748	15.9731313	12.2264047	12.6536051	65.2944198	-4.4062283	0.368321	-11.963011	5.5512E-33	4.4142E-32
TS92_Nanopo	.	CDS	22430	23140	+	TS92_00020	azlC	NA	branched-chain amino acid permease	NA	840	866	773	19	16	18	5	18	14	580.873255	573.041072	500.8293	27.2039202	18.1722718	22.6972124	7.98656563	18.3396071	14.7625393	195.989527	-4.5693078	0.25660163	-17.80701	6.2353E-71	1.5715E-69
TS92_Nanopo	.	CDS	2412409	2413152	-	TS92_02517	glnQ_2	NA	glutamate ABC superfamily ATP binding cassette%0Atransporter%2C ABC protein	NA	609	908	558	8	25	10	10	22	24	421.13311	600.832902	361.530077	11.4542822	28.3941747	12.6095625	15.9731313	22.4150754	25.3072102	166.627725	-4.5914137	0.33215542	-13.823088	1.8496E-43	2.1238E-42
TS92_Nanopo	.	CDS	2347602	2348573	-	TS92_02452	sstA	NA	iron (Fe+3) ABC superfamily ATP binding%0Acassette transporter%2C membrane protein	NA	1150	1314	1027	20	26	26	8	32	21	795.243147	869.487261	665.396754	28.6357054	29.5299417	32.7848624	12.778505	32.603746	22.1438089	276.511526	-4.6254043	0.25060142	-18.457215	4.5627E-76	1.3048E-74
TS92_Nanopo	.	CDS	349907	350020	+	TS92_00330	TS92_00330	NA	hypothetical protein	NA	134	90	117	2	2	0	6	11	12	92.6631145	59.553922	75.8046935	2.86357054	2.27153397	0	9.58387876	11.2075377	12.6536051	29.6224284	-4.9547114	0.60558401	-8.1817078	2.7985E-16	1.0894E-15
TS92_Nanopo	.	CDS	232311	233591	+	TS92_00222	citM_1	NA	CitMHS family citrate-magnesium (Mg2+):proton%0A(H+) citrate-calcium (Ca2+):proton (	NA	1050	839	1031	13	13	15	15	17	10	726.091569	555.174895	667.988367	18.6132085	14.7649708	18.9143437	23.9596969	17.32074	10.5446709	228.152496	-5.154287	0.28560787	-18.046726	8.3708E-73	2.2031E-71
TS92_Nanopo	.	CDS	1928446	1928661	-	TS92_02007	TS92_02007	NA	hypothetical protein	NA	559	686	559	1	15	6	14	42	19	386.557321	453.933228	362.17798	1.43178527	17.0365048	7.56573748	22.3623838	42.7924166	20.0348747	145.988026	-5.2892877	0.38819249	-13.625425	2.8276E-42	3.1261E-41
TS92_Nanopo	.	CDS	317885	318616	+	TS92_00302	cysH	NA	Phosphoadenosine phosphosulfate reductase	NA	2175	2446	1987	12	35	25	11	25	23	1504.04682	1618.54326	1287.38398	17.1814233	39.7518446	31.5239062	17.5704444	25.4716765	24.2527431	507.3029	-5.55255	0.24591839	-22.578832	6.997E-113	3.527E-111

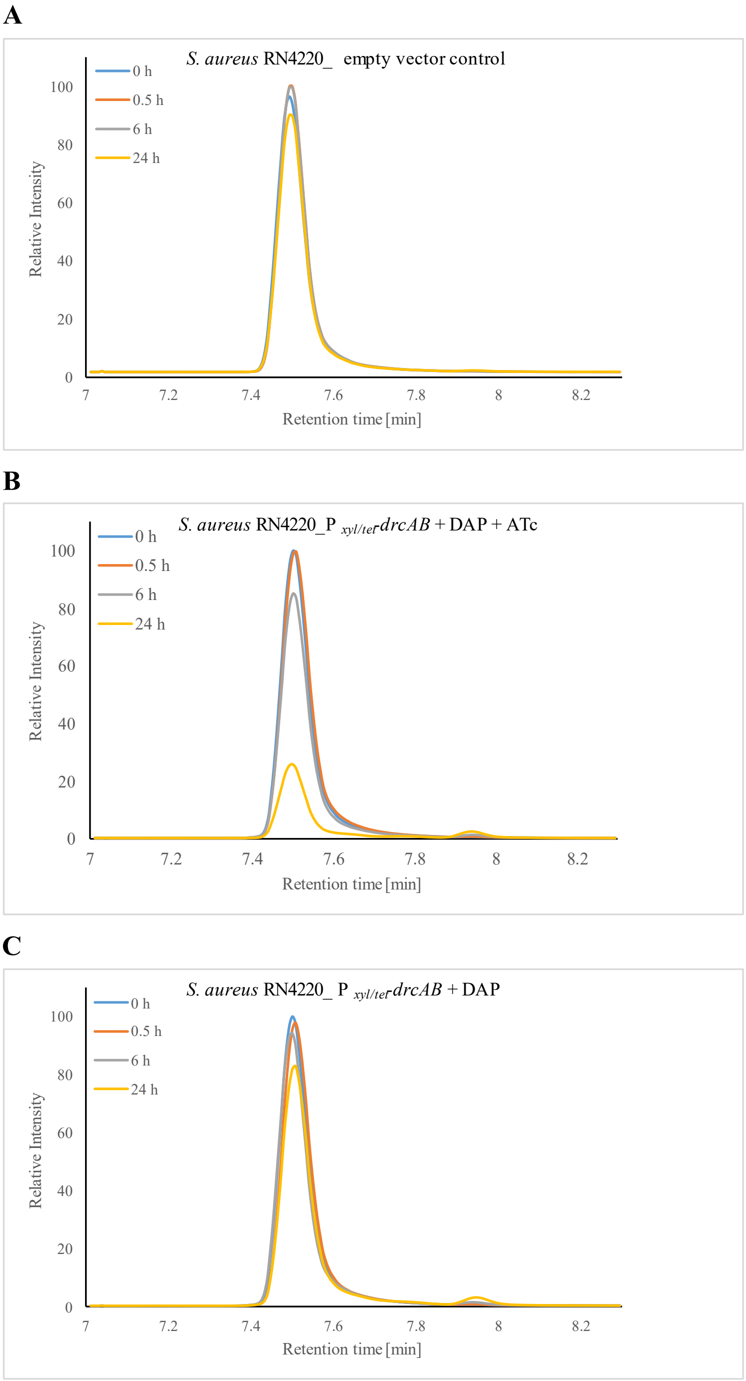

Figure S1. Representative normalized multiple-reaction-monitoring (MRM) chromatograms obtained from LC-MS/MS measurements of DAP in supernatant. (A) *S. aureus* carrying the empty vector as control, **(B)** *drcAB*-expressing *S. aureus* RN4220, and **(C)** non-expressing *S. aureus* RN4220. MRM transitions from 810.9 → 341.1 (m/z) were used for DAP detection. *drcAB* expression was regulated by the ATc-inducible $P_{xyl/tet}$ promoter. Supernatants were collected after 0, 0.5, 6, and 24 hours of incubation. Corresponding chromatograms from the same technical replicates are displayed as overlaid chromatograms.

Table S4. Detection of the *drc* locus (*drcRS/drcAB*) in various bacterial strains and species.

Species, Strain	Accession	Nucleotide identity (%) with $drcRS/drcAB_{TS92}$	*drc* association with MGE
Mammaliicoccus sciuri strain TS92	PP236779	100 %	IS
Mammaliicoccus sciuri strain SNUC 5594	PZGV01000123.1	99.98 %	IS
Mammaliicoccus lentus strain 7048	CP118848.1	99.34 %	IS
Mammaliicoccus lentus strain C9 RVCC933	NZ_JAAQRX010000032	99.28 %	IS
Bacillus thermotolerans strain MTCC 8252	NZ_JWIR02000084	98.65 %	IS
Siminovitchia sp. 179-K 8D1	NZ_JBFEGN010000115.1	97.75 %	IS
Lederbergia galactosidilytica strain G25-74 G25-74	NZ_LDJR01000055.1	97.73 %	IS
Oceanobacillus sp. FSL K6-2867	CP150144.1	97.71 %	IS
Siminovitchia fortis strain XLM16	CP126113.1	97.69 %	IS
Siminovitchia fordii DSM 16014	NZ_KB894686.1	97.64 %	IS

**Species, Strain**	**Accession**	**Nucleotide identity (%) with *drcRS/drcAB*$_{TS92}$**	***drc* association with MGE**
Lederbergia galactosidilytica strain DSM 15595	NZ_JAGGKH010000030.1	97.62 %	IS
Bacillus sp. IITD106 26	JAIAZY010000026.1	97.62 %	IS
Oceanobacillus jeddahense strain QA-1986 374	CP101914.1	97.58 %	IS
Lederbergia ruris strain 179-F 6C2 HS	NZ_JBFEGE010000002.1	97.58 %	IS
Piscibacillus halophilus strain DSM 21633	NZ_FOES01000052.1	97.57 %	IS
Oceanobacillus oncorhynchi strain QA-1986 526	CP101913.1	97.56 %	IS
Shouchella clausii strain 088AE	CP031128.1	97.56 %	IS
Shouchella clausii strain DSM 8716	CP019985.1	97.56 %	IS
Shouchella clausii strain AO1125	CP173278.1	97.56 %	IS
Shouchella clausii strain BC112	NZ_PZQP01000210.1	97.55 %	IS
Siminovitchia sp. FSL H7-0308	CP150282.1	97.53 %	IS
Oceanobacillus jeddahense strain 45476	NZ_JBHTNK010000016.1	97.53 %	IS

Species, Strain	Accession	Nucleotide identity (%) with ***drcRS/drcAB*$_{TS92}$**	***drc* association with MGE**
Shouchella clausii strain TA 149	NZ_JAMAYQ010000015.1	97.53 %	IS
Gracilibacillus alcaliphilus strain DSM 102988	NZ_JAFBFA010000025.1	97.44 %	IS
Enterococcus faecalis CVM N59689F	PTUJ01000031.1	97.40 %	IS
Enterococcus faecalis strain FSIS 12032216	AAXCZN010000013.1	97.23 %	IS, plasmid (uncertain)
Siminovitchia fordii strain J1TS3	NZ_BOQT01000003.1	97.23 %	IS
Enterococcus faecalis strain FSIS 11808951	AAXEJZ010000069.1	97.21 %	IS
Oceanobacillus alkalisoli strain APA_J-2 (6-2)	NZ_JAKGBW010000006	97.18 %	IS
Vagococcus fluvialis strain UFMG-H7	NZ_JAAVMB010000012.1	96.99 %	IS
Oceanobacillus sp. HCA-5259	NZ_JBBKBV010000015.1	96.79 %	IS
Oceanobacillus sp. FSL K6-0127	CP150287.1	96.68 %	IS
Oceanobacillus kimchii X50	NZ_CM001792.1	96.66 %	IS
Oceanobacillus luteolus strain AMY 52	NZ_JAMBON010000049.1	96.63 %	IS

Species, Strain	Accession	Nucleotide identity (%) with *drcRS/drcAB*$_{TS92}$	*drc* association with MGE
Virgibacillus sp. YIM 98842	NZ_WIXN01000011.1	96.62 %	IS
Oceanobacillus polygoni strain DSM 107338	NZ_JAGGMB010000019.1	96.57 %	IS
Pseudogracilibacillus sp. ICA-222130	NZ_JBBKBG010000025.1	96.46 %	IS
Ornithinibacillus sp. 4-3	NZ_CP162599.1	96.38 %	IS
Bacillus andreraoultii isolate MGYG-HGUT-01501	NZ_CABKUO010000012.1	96.37 %	IS
Sporosarcina sp. FSL W8-0480	CP150166.1	96.29 %	IS
Siminovitchia sediminis strain CGMCC 1.12295	NZ_JBHUEO010000061.1	96.26 %	IS
Shouchella clausii strain FSL M8-0189	CP155469.1	96.22 %	IS
Shouchella clausii strain FSL M8-0080	CP155470.1	96.20 %	IS
Novibacillus thermophilus strain SG-1	NZ_CP019699.1	85.17 %	core genome

drcRS/drcAB loci were identified by performing BLAST queries of the NCBI database (https://blast.ncbi.nlm.nih.gov/Blast.cgi), using the *drcB*$_{TS92}$ nucleotide and/or DrcB$_{TS92}$ protein sequences as inputs. MGE – mobile genetic element; IS – insertion sequence.

Supplementary References

1 Schoenfelder, S. M. *et al.* Antibiotic resistance profiles of coagulase-negative staphylococci in livestock environments. *Vet Microbiol* **200**, 79-87, doi:10.1016/j.vetmic.2016.04.019 (2017).

2 Monk, I. R. & Foster, T. J. Genetic manipulation of Staphylococci-breaking through the barrier. *Front Cell Infect Microbiol* **2**, 49, doi:10.3389/fcimb.2012.00049 (2012).

3 Spizizen, J. Transformation of Biochemically Deficient Strains of *Bacillus Subtilis* by Deoxyribonucleate. *Proc Natl Acad Sci U S A* **44**, 1072-1078, doi:10.1073/pnas.44.10.1072 (1958).

4 Nair, D. *et al.* Whole-genome sequencing of *Staphylococcus aureus* strain RN4220, a key laboratory strain used in virulence research, identifies mutations that affect not only virulence factors but also the fitness of the strain. *J Bacteriol* **193**, 2332-2335, doi:10.1128/JB.00027-11 (2011).

5 Boles, B. R., Thoendel, M., Roth, A. J. & Horswill, A. R. Identification of genes involved in polysaccharide-independent *Staphylococcus aureus* biofilm formation. *PLoS One* **5**, e10146, doi:10.1371/journal.pone.0010146 (2010).

6 Diep, B. A. *et al.* Complete genome sequence of USA300, an epidemic clone of community-acquired meticillin-resistant *Staphylococcus aureus*. *Lancet* **367**, 731-739, doi:10.1016/S0140-6736(06)68231-7 (2006).

7 Helle, L. *et al.* Vectors for improved Tet repressor-dependent gradual gene induction or silencing in *Staphylococcus aureus*. *Microbiology* **157**, 3314-3323, doi:10.1099/mic.0.052548-0 (2011).
